# An in situ exploration of how Fe/N/C oxygen reduction catalysts evolve during synthesis under pyrolytic conditions

**DOI:** 10.1038/s41467-024-50629-x

**Published:** 2024-07-24

**Authors:** Shuhu Yin, Hongyuan Yi, Mengli Liu, Jian Yang, Shuangli Yang, Bin-Wei Zhang, Long Chen, Xiaoyang Cheng, Huan Huang, Rui Huang, Yanxia Jiang, Honggang Liao, Shigang Sun

**Affiliations:** 1 State Key Laboratory of Physical Chemistry of Solid Surfaces, Engineering Research Center of Electrochemical Technologies of Ministry of Education, College of Chemistry and Chemical Engineering, and Discipline of Intelligent Instrument and Equipment, Xiamen University, Xiamen, 361005 P. R. China; 2 Center of Advanced Electrochemical Energy, Institute of Advanced Interdisciplinary Studies, School of Chemistry and Chemical Engineering, Chongqing University, Chongqing, 400044 PR China; 3 Beijing Synchrotron Radiation Facility, Institute of High Energy Physics, Chinese Academy of Sciences, 100049 Beijing, P. R. China

**Keywords:** Fuel cells, Electrocatalysis, Catalytic mechanisms, Electrocatalysis

## Abstract

In pursuing cheap and effective oxygen reduction catalysts, the Fe/N/C system emerges as a promising candidate. Nevertheless, the structural transformations of starting materials into Fe- and N-doped carbon catalysts remains poorly characterized under pyrolytic conditions. Here, we explore the evolution of Fe species and track the formation of Fe–N_4_ site development by employing diverse in-situ diagnostic techniques. In-situ heating microscopy reveals the initial formation of FeO_x_ nanoparticles and subsequent internal migration within the carbon matrix, which stops once FeO_x_ is fully reduced. The migration and decomposition of nanoparticles then leads to carbon layer reconstruction. Experimental and theoretical analysis reveals size-dependent behavior of FeO_x_ where nanoparticles below 7 nm readily release Fe atoms to form Fe–N_4_ while nanoparticles with sizes >10 nm tend to coalesce and impede Fe–N_4_ site formation. The work visualizes the pyrolysis process of Fe/N/C materials, providing theoretical guidance for the rational design of catalysts.

## Abstract (web summary)

Fe-N-C material is promising catalyst for oxygen reduction reaction in proton exchange membrane fuel cell. Here the authors visualize the formation of Fe-N4 sites using in situ heating microscopy, providing theoretical guidance for rational catalyst design of Fe-N-C materials.

## Introduction

The commercialization of fuel cell electric vehicle based on the proton exchange membrane fuel cells (PEMFCs) in Japan in 2014 was an important milestone. However, the extensive use of platinum in these vehicles is a major impediment to their widespread and sustainable adoption^1–4^. Platinum is primarily utilized to catalyze the oxygen reduction reaction (ORR) of cathode, and the research efforts of the global scientific community are concentrated on the development of oxygen reduction catalysts that do not contain Platinum group elements^5^. A single transition metal atom (M = Mn, Fe or Co) embedded in nitrogen doped carbon matrix (named as M/N/C) is the primary catalytic element of the platinum free group, used for the oxygen reduction reaction in PEMFCs^6,7^. We recently reported a Fe/N/C catalyst with a performance of 710 mW cm^−2^ and 120.8 mA cm^−2^ at 0.8 V in H_2_-air PEMFCs^8^, which is more than most of the reported M/N/C catalysts. However, it is not enough to power automotive fuel cells. To further enhance performance, it is necessary to increase the turnover frequency (TOF) and site densities (SD) of active sites in M/N/C materials^9^. However, due to the lack of understanding of the formation of active sites during the synthesis of M/N/C catalysts, these two aspects are impeded. The production of M/N/C catalysts typically involves the amalgamation of M, N, and C sources into a unified compound or, alternatively, the utilization of a separate compound followed by pyrolysis within a temperature range of 900 to 1100 ^o^C^7,10–12^. The enhancement of catalytic activity predominantly relies on empirical approaches, including the selection of appropriate precursors, adjustment of metal content, and optimization of pyrolysis conditions. The dynamics of competition between metal-based active sites and by-products during pyrolysis remain elusive^13^. To effectively synthesize upgraded M/N/C catalysts, it is necessary to understand the “black box” synthesis process of the current input precursor and output product.

The complexity of the structure of M/N/C catalysts is largely due to the pyrolysis process, which was proved to be necessary for the production of highly active M/N/C catalysts for ORR in acidic media in the 1980s^14^. The products of high-temperature pyrolysis are usually conglomerates of various components, including N-doped carbon defects and edges^15^, MN_x_ groups^16^, and inorganic particles such as metals, metal oxides, carbides, and nitrides^17,18^. According to the research in the past two decades, the Fe^II^**–**N_4_ sites has a high TOF for ORR, which may be the main contributor to the ORR activity of the pyrolytic Fe/N/C catalyst^19^. It has been demonstrated that the effectiveness of Fe/N/C catalysts can be augmented through an increase in the concentration of Fe^II^**–**N_4_ sites. In the realm of PEMFCs, the preponderance of Fe/N/C catalysts that deliver superior performance typically comprises less than 3 wt% Fe^8,9,20–23^. Notwithstanding reports of catalysts with Fe contents surpassing 3 wt%, these have yet to exhibit exceptional performance in PEMFCs applications^24–26^. What is the process for forming the Fe^II^**–**N_4_ sites during pyrolysis when utilizing the trial-and-error synthesis of Fe/N/C catalysts? Wu et al.^27^ recently identified a fascinating occurrence wherein ultra small FeO_x_ particles undergo a transformation to atomic dispersed Fe**–**N_4_ sites during pyrolysis, which was uncovered through ex situ aberration correction of HAADF-STEM. Jia et al.^28^ revealed by variable temperature X-ray absorption spectroscopy (XAS) that the Fe-doped precursor was converted to Fe oxide below 300 ^o^C, and then converted to tetrahedral Fe^II^**–**O_4_ below 600 ^o^C through a crystal-to-melt process. At temperatures above 600 °C, Fe^II^**–**O_4_ releases a single Fe atom which diffuses into N doped carbon defects, thus forming Fe^II^**–**N_4_. These findings, however, cannot explain the origin of iron oxides, let alone directly observe the thermal activation process. They only indicate that Fe oxides can be transformed into Fe**–**N_4_ sites at high temperatures. Recently, Wu et al. reported a successful preparation of Fe/N/C catalyst (Fe-AC) with *E*_1/2_ up to 0.915 V using commercial Fe_2_O_3_ nanoparticles with a size of ~5 nm^23^. Jia et al.^21^ and our previous work^8^ have reported that a novel non-contact vapor deposition technique has been adopted to directly gasify Fe precursors and deposit them on carbon carriers, achieving ultra-high PEMFCs performance. What are the advantages of using 5 nm Fe_2_O_3_ nanoparticles as the iron source, and why is the non-contact vapor deposition method capable of achieving high performance?

Here, we directly observed the dynamic evolution process from Fe salt to Fe–N_4_ sites during pyrolysis through a variety of in situ characterizations, visualized the “black box” process of pyrolysis, and further clarified the structural transformation process of Fe/N/C materials during thermal activation, which successfully explained the reasons behind these excellent strategies.

## Results

### The dynamic evolution process of iron oxide nanoparticles

**Fig. 1:**
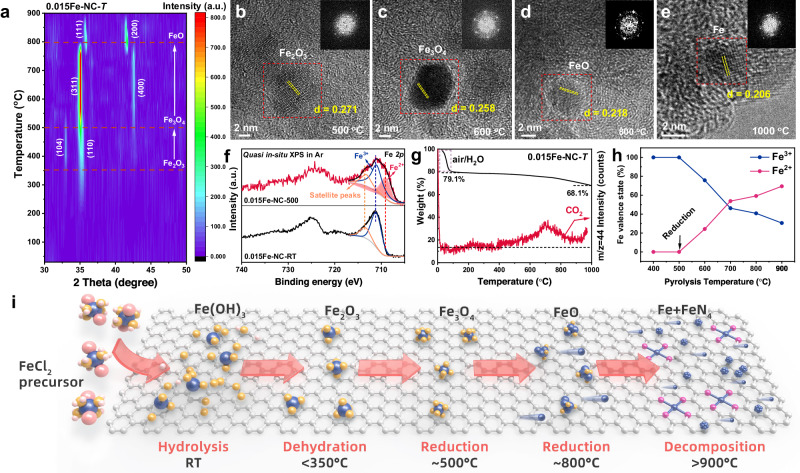
Phase transformation process during pyrolysis. **a** Contour maps of in situ heating XRD. **b–e** Series of HRTEM images acquired at different temperatures during in situ heating TEM. **f** Quasi in situ XPS Fe *2p* spectra before and after 500 ^o^C treatment. **g** TGA-MS thermogram; (**h**) the variation trend of Fe oxidation status as a function of the pyrolysis temperature. **i** Schematic diagram of phase transformation process during pyrolysis.

The phase transition of iron species was monitored during pyrolysis process of the precursor by in situ heating XRD and TEM experiments. FeCl_2_·4H_2_O (with a Fe loading of 1.5 wt%) and nitrogen-doped carbon substrate (NC) were pulverized and combined, forming a sample referred to as 0.015Fe-NC-*T*, which was then placed in a ceramic sample tank to detect the structural evolution during heating in real time. Figure 1a displays the contour line diagram of the XRD spectrum in the chosen range of angles (original data and the characteristic peak was identified in Supplementary Fig. 1). The data indicates that the precursor is amorphous in its initial state at room temperature, and it progresses through two phase transitions as it is heated. At temperatures of 350 °C and above, a hexagonal Fe_2_O_3_ structure (R-3c) is activated. As the thermal activation temperature rises to 500 °C, the hexagonal Fe_2_O_3_ structure begins to transform into a cubic Fe_3_O_4_ structure (Fd-3m). This transformation is confirmed when the (104)_R_ peak of 33.1^o^ in the hexagonal structure disappears at 550 °C and the (110)_R_ peak of 35.6^o^ shifts towards the (311)_R_ peak of 35.4^o^ in the low angle cubic structure. When the thermal activation temperature increased to 800 °C, the cubic Fe_3_O_4_ phase transformed into the cubic FeO phase (Fm-3m). At 850 °C, the (111)_R_ peak at 35.8^o^ and the (200)_R_ peak at 41.6^o^ moved towards a lower angle, suggesting a substantial lattice expansion. We monitored the progression from nanoparticles to single atoms in a vacuum atmosphere, within a temperature range of 500 °C to 1000 °C. Figure 1b–e demonstrates the iron oxide nanoparticles (FeO_x_) and their corresponding Fast Fourier Transform (FFT) pattern at varying temperatures (Supplementary Figs. 2–4), consistent with XRD results, and eventually restoring FeO to Fe (Supplementary Fig. 5). The formation of FeO_x_ is a result of the dehydration of Fe(OH)_3_ at 350 ^o^C. It has been proved that Fe(OH)_3_ is formed by the oxidative hydrolysis of FeCl_2_·4H_2_O at room temperature (Supplementary Figs. 6-11, details in Supplementary Note 1). *Quasi* in situ heating XPS was further employed to explore the reduction process of FeO_x_ (Supplementary Figs. 12–13). After heat treatment at 500 ^o^C for 10 min in Ar atmosphere, 33.73% Fe^3+^ was reduced to Fe^2+^ (Fig. 1f, Supplementary Table 1). This is due to the carbothermal reaction at 500 ^o^C, which is evidenced by the appearance of CO_2_ (m/z = 44) as observed through online TGA-MS characterization (Fig. 1g). The carbothermic reaction causes the valence state of Fe to decline from +3 to +2, which is corroborated by the XPS Fe *2p* fine spectrum results (Fig. 1h, Supplementary Fig. 14 and Table 2). The above experiments reveal that the 0.015Fe-NC-*T* precursor undergoes a carbothermic reaction during pyrolysis, resulting in a gradual reduction of FeO_x_ from Fe_2_O_3_ to Fe_3_O_4_, then FeO, and finally to Fe (Fig. 1i).

**Fig. 2:**
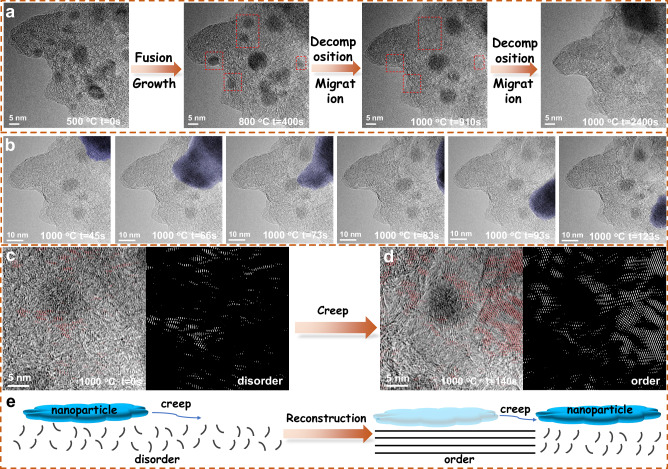
The dynamic process of nanoparticles. **a** Time-series TEM images during pyrolysis from Supplementary Movie 1. **b** Time-series TEM images of nanoparticle creeping process. HRTEM images of carbon structure and the corresponding inverse FFT patterns in the same region before (**c**) and after (**d**) nanoparticles creep. **e** Schematic diagram of carbon layer reconstruction by nanoparticle creep.

The formation and decomposition processes of FeO_x_ were further analyzed. Figure 2a displays representative movie images at different pyrolysis temperatures. The Supplementary Movie 1 shows the creation of several nanoparticles at 500 °C. Fusion growth behavior was observed at 800 °C, where larger nanoparticles expanded and eventually formed low surface energy spheres resembling Ostwald ripening, while smaller particles were eliminated. At 1000 °C, the larger nanoparticles began to disintegrate and move, ultimately resulting in the production of only a small number of clusters or large nanoparticles, with the majority of iron atoms being dispersed evenly. Throughout the pyrolysis process, nanoparticles larger than 10 nm exhibited creep, migration, and the production of carbon layers (Fig. 2b). The migration of nanoparticles was primarily driven by the size-dependent reduction in their melting point^29^ and the gas production from carbothermal reactions occurring on their surfaces^30,31^. These gases create a high local pressure on one side of the nanoparticle, which drives the nanoparticle to migrate. The inverse FFT frequencies of the HRTEM images were used to recover the spatial distribution of ordered carbon structure in the area before and after the creep of the nanoparticles (Figs. 2c, d, Supplementary Figs. 16–19). It is evident that the carbon layer disrupted by the nanoparticle creep was reconstructed, resulting in a significant increase in order. In essence, the migration of nanoparticles results in the rebuilding of disordered carbon layers, leading to the formation of an ordered carbon structure (Fig. 2e).

**Fig. 3:**
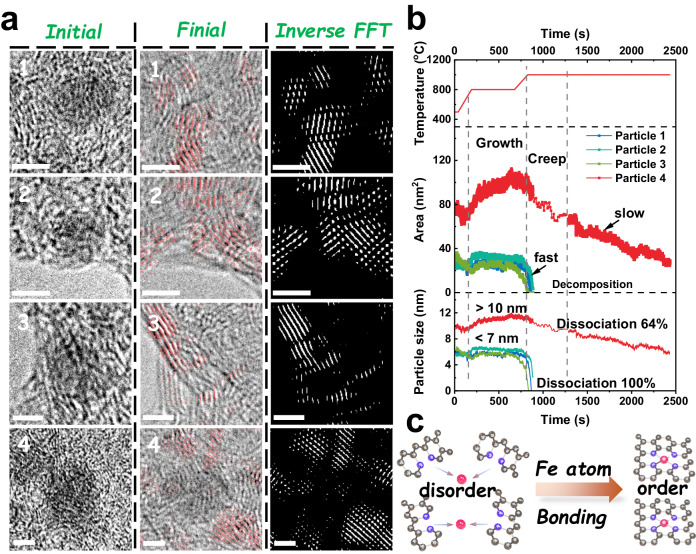
The evolution process of four typical nanoparticles. **a** Adjacent carbon structure of four typical nanoparticles in the initial and final HRTEM images and the corresponding inverse FFT patterns during pyrolysis. The scale bar is 5 nm. **b** The projection-area and particle size variation as a function of time of nanoparticle 1, 2, 3 and 4. **c** Schematic diagram of disordered carbon segments to ordered FeN_4_ sites.

Similarly, a single Fe atom can also migrate on the carbon layer under thermal and gas driving. As a result, we followed the structural development of four different regions—including the surrounding carbon layers and nanoparticles—during pyrolysis (Fig. 3a, Supplementary Figs. 20–23). Significant improvements have been made to the ordered carbon structure following the breakdown of the nanoparticles. Through extensive data analysis, we have gained a profound understanding of the projection area and size of nanoparticles during pyrolysis (Supplementary Movies 2, 3, and Note 2). The two distinct growth-decomposition paths depicted in Fig. 3b are as follows: smaller nanoparticles (Particles 1, 2, and 3; less than 7 nm) undergo slow growth followed by quick complete decomposition, whereas larger nanoparticles (Particle 4, more than 10 nm) undergo rapid growth followed by slow partial decomposition, maintaining 36% of the nanoparticles. The progressive disintegration of the nanoparticles, which resulted in their decomposition, was caused by the surface carbothermic reaction. The Fe atoms generated by the surface carbothermic reaction become more active and activated, which promote their migration through the carbon layer and significantly boost the production of Fe-N_x_ sites. Meanwhile, the amalgamation of these Fe atoms with carbon atoms on the surface results in the formation of carbon-rich and carbon-poor areas, prompting carbon migration toward the surface and the generation of an ordered carbon layer (Supplementary Fig. 24)^32,33^. The development of Fe-N_x_ sites and the rebuilding and bending of the carbon layer during pyrolysis may be connected to the movement of iron atoms on the carbon surface, even if the migration of individual Fe atoms was not directly detected (Fig. 3c). Furthermore, Fe single atoms embedded in the ordered carbon layer were visible in the aberration corrected high-angle annular dark field scanning transmission electron microscopy (AC-HAADF-STEM) image of 0.015Fe-NC-900, and electron energy-loss spectra (EELS) simultaneously detected Fe and N, suggesting the formation of Fe-N_x_ sites (Supplementary Fig. 25). Nevertheless, converting nanoparticles larger than 10 nm completely into monodisperse Fe-N_x_ sites proves challenging as they retain the crystallinity of Fe derivatives even after ceasing their movement (Supplementary Fig. 26).

### The transformation mechanism from FeO_x_ nanoparticles to Fe–N_x_ sites

**Fig. 4:**
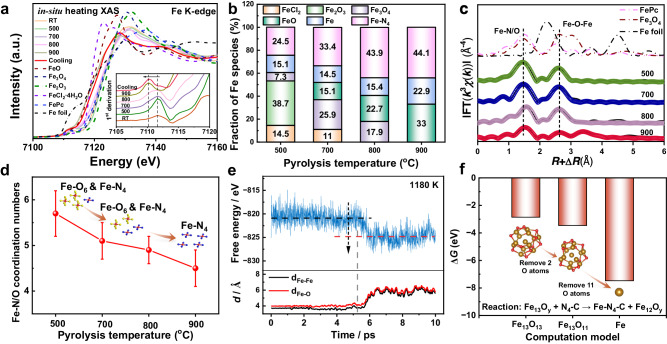
In situ heating XAS experiment of 0.015Fe-NC-*T* samples. **a** Fe K-edge XANES during pyrolysis. **b** Fraction of Fe species at different pyrolysis temperature. **c** Fe K-edge EXAFS fitting in *R* space. **d** The Fe-N/O coordination numbers as a function of the pyrolysis temperature. **e** The system free energy and bond length variation as a function of time. **f** The Δ*G* of different reactions. Error bars represent the standard EXAFS-fitting error.

We performed in situ heating X-ray absorption spectra (XAS) experiments to further investigate the microstructural changes of Fe atoms during the precursor thermal activation. In comparison to the as-received FeCl_2_·4H_2_O, the X-ray absorption near-edge structure (XANES) spectrum of 0.015Fe-NC-RT was more similar to FeOOH standard (Supplementary Figs. 27–29). Then following treatment at 500 ^o^C, the Fe species was transformed into Fe_3_O_4_ standard (Supplementary Fig. 30). The edge energy gradually shifted negatively as the thermal activation temperature gradually rose to 900 ^o^C (Fig. 4a, Supplementary Fig. 31), supporting the XPS result that the oxidation state of Fe was decreased. The correlation between the composition of Fe species and pyrolysis temperature was established using linear combination fitting (LCF), as shown in Supplementary Fig. 32, Tables 4–5 (details were in Supplementary Note 3). As the pyrolysis temperature increased, the transformation of FeO_x_ into Fe–N_x_ sites was observed, with a maximum conversion rate of 44.1% (Fig. 4b). The coordination of Fe during the precursor’s thermal activation was determined through EXAFS-fitting (Fig. 4c). Analysis revealed that as the thermal activation temperature increased, the coordination number of the Fe-N/O bond decreased from 5.7 to 4.5 (Fig. 4d, Supplementary Table 6). The decrease in the average coordination number was attributed to the increase in the proportion of Fe–N_4_ sites, resulting in a lower overall coordination number. The conversion of FeO_x_ to Fe–N_4_ sites was further elucidated using Fe_13_O_13_ as the calculation model. Fe_13_O_13_ clusters began to release a Fe atom into the N_4_-C defect at 5.7 ps under 1180 K (Supplementary Fig. 33, Movie 4 and Data 1). This caused a decrease in system free energy and the elongation of Fe-Fe and Fe-O bonds (Fig. 4e). The carbothermal reaction facilitated the conversion process by removing oxygen atoms from Fe_13_O_13_, as evidenced by the increase in negative Δ*G* with decreasing oxygen atoms. This revealed that at high temperatures, FeO_x_ may really convert a single Fe atom to a Fe–N_4_ site, and the carbothermal reaction will help in this process. Once the nanoparticles come into close proximity, the sintering of iron species will occur, impeding the conversion of Fe atoms to Fe–N_4_ sites^34^. The ORR performance was significantly enhanced when Fe_2_O_3_ or Fe_3_O_4_ and NC support were directly pyrolyzed, as demonstrated in Supplementary Figs. 34–35, indicating that FeO_x_ can indeed be converted into highly active Fe–N_4_ sites experimentally. Additionally, the pyrolysis temperature influences the reaction kinetic energy barrier and determines the conversion reaction rate, rather than the occurrence of the conversion reaction itself. The nanoparticle size affects the release difficulty of a single Fe atom, which is associated with whether the nanoparticles are transformed into Fe-N_x_ sites or undergo further sintering (Supplementary Figs. 36–38).

### Active site conversion rate and catalytic performance

**Fig. 5:**
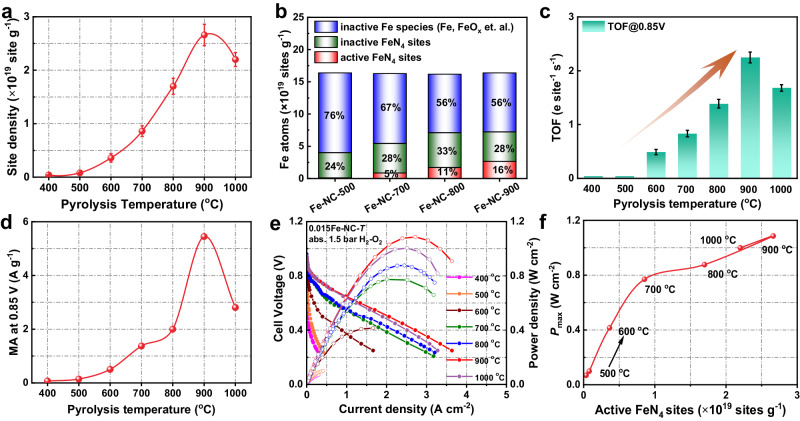
The correlation between structure and catalytic performance of 0.015Fe-NC-*T* samples. **a** The site density as a function of the pyrolysis temperature; (**b**) the Fe atoms number of different Fe species; (**c**) TOF@0.85 V as a function of the pyrolysis temperature; (**d**) the MA at 0.85 V as a function of the pyrolysis temperature; (**e**) H_2_-O_2_ fuel cell *i*-*V* polarization (solid symbols and lines) and powder density (hollow symbols and lines) plots record under absolute 1.5 bar of O_2_ pressure; (**f**) the relationship between *P*_max_ and site density. The cell voltage and power density are not iR corrected. Error bars represent the standard deviation for three separate measurements.

The active Fe–N_4_ site densities of materials subjected to varying thermal activation temperatures were quantitatively assessed via nitrite poisoning experiment^35^. Initially, the Fe_2_O_3_-NC material underwent the nitrite poisoning experiment to eliminate the interference from FeO_x_ species (Supplementary Fig. 39). As depicted in Fig. 5a, an exponential relationship emerged between site density (SD) and pyrolysis temperature, indicating a strong correlation (Supplementary Figs. 40-41). Notably, active Fe–N_4_ sites were scarce at temperatures below 500 °C. A marked increase in active Fe–N_4_ site density was observed at 600 °C, suggesting the onset of active Fe–N_4_ formation. The density of highly active Fe–N_4_ sites continued to climb with increasing pyrolysis temperatures, reaching a peak of 2.66 × 10^19^ sites g^–1^ at 900 °C (Fig. 5a, Supplementary Table 7). At 1000 ^o^C, the SD value decreased due to the loss of N. Combining the ICP-MS, LCF fitting and nitrite poisoning results, active Fe–N_4_ sites, inactive Fe–N_4_ sites and inactive Fe species are distinguished (Supplementary Tables 7–9, the detailed quantitative process is below Supplementary Table 9.). Remarkably, only 44% of the total Fe atoms were converted into Fe–N_4_ sites (Fig. 5b), while the majority were converted into inactive Fe species (such as Fe, FeO_x_, Fe_x_C, etc.). More concerning was the finding that a mere 16% (obtained by nitrite poisoning) of Fe atoms constituted active Fe–N_4_ sites involved in the ORR process. The ORR performance of the 0.015Fe-NC-*T* was evaluated through a rotating disk electrode (RDE) in an O_2_-saturated 0.1 M H_2_SO_4_ electrolyte. Upon elevating the pyrolysis to 600 °C, a notable increase in ORR activity was observed (Supplementary Fig. 42). Meanwhile, the turnover frequency at 0.85 V (TOF@0.85 V) for 0.015Fe-NC-600 exhibited a substantial rise, as demonstrated in Fig. 5c. It is evident that highly active Fe–N_4_ sites commenced to form at 600 °C. The ORR activity and TOF@0.85 V continued to ascend with temperature increments, reaching a half-wave potential (*E*_1/2_) of 0.84 V (versus the reversible hydrogen electrode) at 900 ^o^C (Supplementary Fig. 42). The mass activity (MA) at 0.85 V escalated in tandem with the proliferation of highly active Fe–N_4_ sites (Fig. 5d). To ensure the veracity of these findings, real-world application tests were imperative. A pronounced surge in peak power density (*P*_max_) was observed at 600 °C—the onset temperature for active Fe–N_4_ site formation—during a steady-state H_2_-O_2_ PEMFCs operation at 1.5 bar absolute pressure (Fig. 5e, f). This indicated a correlation between the quantity of highly active Fe–N_4_ sites and PEMFC performance. The *P*_max_ rose to 1.09 W cm^-2^ concomitant with the increase in highly active Fe–N_4_ sites. Impressively, the fuel cell exhibited robust performance even though merely 16% of the Fe atoms constituted highly active Fe–N_4_ sites, further underscoring the promise of Fe/N/C as an excellent platinum group metal-free (PGM-free) catalyst. The pyrolysis process managed to convert only 44% of the FeO_x_ into Fe–N_4_ sites, leaving the remainder as either FeO_x_ or metallic Fe clusters. Using AC-HAADF-STEM, we identified a significant presence of Fe species clusters (Supplementary Fig. 43).

Consequently, a limited number of Fe–N_4_ sites are actively utilized by the pyrolyzed Fe/N/C compound. There are two potential reasons: firstly, more than half of the Fe atoms fail to evolve into Fe–N_4_ sites; secondly, those Fe active centers that do form may lose its ability to function because of a dense layer of carbon around it. Based on our experimental results, there are the following four suggestions for the rational design of Fe/N/C catalysts:

1. The FeO_x_ nanoparticle sizes must be reduced to below 7 nm during pyrolysis process. With this, more Fe–N_4_ sites can be created;
2. Substances that decompose into acidic gases during the pyrolysis process, such as NH_4_Cl, NH_4_Br, and others, can be introduced. NH_4_Cl has been shown to remove nanoparticles at high temperatures, contributing to the formation of atomically dispersed Fe sites^23,36^. Besides, FeO_x_ can be produced and their development inhibited or even stopped by reacting with acidic gasses;
3. It is also possible to prevent FeO_x_ formation at its source, such as non-contact vapor deposition strategy^8,21^;
4. Investigating the efficient utilization of the synthesized Fe active centers is paramount; for instance, leveraging the porous structure of catalysts is a valuable approach to enhance site efficacy.

Overall, these strategies can be combined to improve the conversion and utilization of Fe active centers during pyrolysis process in high-performance Fe/N/C catalyst design.

## Discussion

In summary, the complex structural evolution of Fe/N/C catalysts during thermal activation has been thoroughly analyzed by utilizing a series of in situ characterizations. Firstly, the complete transformation process of the FeO_x_ nanoparticles, including expansion, decomposition, diffusion and migration, was directly observed by us. This observation demonstrated that the formation and phase transition of FeO_x_ during thermal activation are caused by the oxidative hydrolysis of precursors. Through extensive data analysis, we gained a profound understanding of the evolutionary behavior of nanoparticles during pyrolysis. Specifically, nanoparticles smaller than 7 nm exhibited gradual growth followed by rapid decomposition, whereas those larger than 10 nm demonstrated swift growth with a slower decomposition phase. Furthermore, DFT calculations and molecular dynamics simulations revealed that the Fe atom gradually detaches from the FeO_x_ as a result of the carbothermic reaction, creating the Fe–N_4_ site. Simultaneously, the diffusion of iron atoms can also lead to the reorganization of the carbon layer, forming an organized structure. Nonetheless, the transition of Fe–N_4_ sites is influenced by the size of FeO_x_ nanoparticles. Based on the findings of our investigation, it is recommended that either the creation of FeO_x_ should be prevented or the nanoparticle size of FeO_x_ should be lowered to less than 7 nm during the thermal activation process in order to produce more Fe–N_4_ sites. This work reveals the formation process of active center in Fe/N/C catalysts from the source, and provides strong evidences for rational design of Fe/N/C catalysts.

## Methods

### Synthesis of the Zn-based zeolitic imidazolate framework (ZIF8)

In our previous work^8^, Zn(NO_3_)_2_·6H_2_O (10 mmol, 2.975 g) and 2-mIm (80 mmol, 6.568 g) were each dissolved in 100 mL of methanol using ultrasound for 5 min. The Zn(NO_3_)_2_ solution was then quickly added to the 2-mIm solution. The mixture was vigorously stirred for 16 h at room temperature. The resulting white ZIF-8 precipitate was centrifuged, washed multiple times with methanol, and dried under vacuum at 60 ⁰C overnight.

### Synthesis of NC

1.0 g ZIF-8 and 0.3 g 1,10-phenanthroline were dispersed in a 2:1 ethanol and deionized water solution. The mixture was stirred magnetically for 12 h at room temperature, then evaporated in an 80 °C oil bath. The dried powders were ground thoroughly and pyrolyzed under Ar at 1000 °C (5 °C min^–1^) for 1 h, then cooled naturally to room temperature. The resulting black products were named NC.

### Synthesis of *x*Fe-NC-*T*

An appropriate amount of FeCl_2_·4H_2_O and 100 mg NC (with Fe feed ratio converted to *x*) were thoroughly ground in an agate mortar. The *x*Fe-NC-*T* was synthesized by heating the mixture in an Ar atmosphere at a specific temperature (*T*) for 1 h (5 °C min^–1^).

### Synthesis of 0.015FeO_x_-NC-900

An appropriate amount of FeO_x_ and 100 mg NC (with Fe feed ratio converted to 0.015) were thoroughly ground in an agate mortar. The 0.015FeO_x_-NC-900 was obtained by heating the mixture in an Ar atmosphere at 900 °C for 1 h (5 °C min^−1^).

### In situ heating TEM

For in situ analysis, the as-prepared 0.015Fe-NC-*T* was firstly dispersed into deionized water. The suspension was sonicated for 20 min at room temperature, and then deposited directly onto a homemade heating chip purchased from CHIP-NOVA company. The in situ heating TEM experiments were performed on TECNAI F20 at a unit acceleration voltage of 200 kV in a vacuum atmosphere and a heating control system. The TEM specimen was heated from 20 to 1000 ^o^C. To observe the temperature range of the variation of 0.015Fe-NC-*T*, the temperature of 500 ^o^C, 800 ^o^C and 1000 ^o^C was maintained for 10 min–30 at each increment and the image was taken when the sample was stable.

### In situ heating XRD

X-ray power diffraction (XRD) patterns were taken on a Rigaku Ultima IV diffractometer (Rigaku, Japan) with Cu Kα X-ray source. The sample was fully ground and filled to a ceramic sample groove, and then transferred into the quasi in situ reaction cell for treatment under various conditions that were comparable with the real reaction conditions. The range of 30°–50° was the position of the main peak of Fe species, so it was selected to analyze the transformation process of Fe species during heat treatment. The scanning rate was 1° min^−1^ and the step length is 0.02° min^–1^. During the test, Ar was continuously injected, the flow rate was 15 mL min^–1^, the heating rate was 10 °C min^–1^, and the constant temperature time of each temperature was 5 min.

### Quasi in situ heating XPS

X-ray photoelectron spectroscopy (XPS) measurements were conducted on an Omicron Sphera II Hemispherical electron energy analyzer with monochromatic Al Kα radiation (1486.6 eV) operated at 15 kV and 300 W. The base pressure of the systems was 5.0 × 10^–9^ mbar. The sample was placed into the sample tank, compressed, secured on the stainless steel sample frame, and then transferred to the quasi in situ reaction pool. It was heated to 500 ^o^C at a rate of 10 ^o^C min^–1^ in an Ar atmosphere for 10 min. Subsequently, the product is to be transferred to the vacuum chamber for XPS test.

### In situ heating XAS

The in situ heating XAS measurement was performed at beamline 1W1B of the Beijing Synchrotron Radiation Facility (BSRF). The power precursors were fitted into a ceramic groove custom-made with an 8 mm internal diameter and 1.5 mm depth. The Fe K-edge spectra were recorded in fluorescence mode and an iron foil spectrum was measured simultaneously with each sample spectrum for energy calibration. Transmission data was taken from 6950 eV to 7940 eV. Spectra were taken at room temperature, then the quartz tube was heated at a ramp rate of approximately 20 °C min^–1^ under flowing nitrogen to approximately the following temperature set points during the increasing temperature portion of the temperature profile: room temperature, 500 ^o^C, 700 ^o^C, 800 ^o^C and 900 ^o^C. A XAS scans was collected at each temperature set point. The hold time at each temperature was approximately 10 min. The XAS data were processed and fitted using the Ifeffit−based Athena and Artemis programs. Scans were calibrated, aligned, and normalized with background removed using the IFEFFIT suite.

### Electrochemical measurements

All electrochemical measurements were conducted using a CHI 760e electrochemical workstation with a three-electrode setup. The working electrode was the catalyst-modified glassy carbon electrode, with a graphite rod as the counter electrode and a saturated calomel electrode (SCE) as the reference. To prepare a uniform catalyst ink, 6 mg of catalyst was sonicated for 30 min in 1 mL of a mixture containing 600 μL isopropanol, 380 μL ultrapure water, and 20 μL of 5 wt% Nafion solution. For the commercial 20 wt% Pt/C sample, 1 mg of catalyst was dispersed in 1 mL of 0.05 wt% Nafion solution. A precise volume of the catalyst ink was then applied onto the polished glassy carbon rotating disk electrode (RDE, diameter 5 mm, area 0.196 cm²) or rotating ring-disk electrode (RRDE, diameter 5.61 mm, area 0.2475 cm²) to achieve the desired catalyst loading. The catalyst loading was 0.6 mg_total_ cm⁻² for M-N-C and 0.012 mg_Pt_ cm⁻² for Pt/C.

ORR polarization curves were performed at 30 °C in 0.1 M H_2_SO_4_ solution. RRDE measurements utilized linear sweep voltammetry (LSV) in the range of 0.1 to 1.1 V (*vs*. RHE) at 900 rpm with a scan rate of 10 mV s⁻¹ with IR_S_ compensations, while the ring electrode was held at 1.2 V (*vs*. RHE). The resistance is automatically compensated by 80 %, and the resistance in the impedance spectrum mode is measured by the pine system. The electrolyte was measured by pH instruments to ensure a constant test environment (pH = 0.7 for 0.1 M H_2_SO_4_). All potentials were converted to reversible hydrogen electrode (RHE) potentials using the equation:

1 $${E}_{{RHE}}={E}_{{SCE}}+0.2415+0.059\times {pH}-{{IR}}_{S}$$

The electron transfer number (*n*) and H_2_O_2_ percentage were calculated using the following equations:

2 $$n=\frac{4\times {I}_{D}}{{I}_{D}+{I}_{R}/N}$$

3 $${{{{\rm{H}}}}}_{2}{{{{\rm{O}}}}}_{2}\left(\%\right)=\frac{200\times {I}_{R}/N}{{I}_{D}+{I}_{R}/N}$$

where *I*_D_ is the disk current, *I*_R_ is the ring current, and N (0.37) is the current collection efficiency of the Pt ring.

The kinetic current densities (*j*_k_) involved during the ORR process were determined by analyzing Koutecky-Levich (K-L) Eq. (4):

4 $$\frac{1}{j}=\frac{1}{{j}_{k}}+\frac{1}{{j}_{L}}$$

where *j* is the measured current density, *j*_L_ and *j*_k_ are the limiting and kinetic current densities.

### Quantification of active Fe–N_4_ sites

The SD was determined using the nitrite reduction method by Kucernak^35^. Briefly, nitrite forms stable poisoned adducts with Fe metal centers, which can be completely stripped between 0.35 to −0.35 V (*vs*. RHE). The excess coulometric charge (*Q*_strip_) from the stripping peak is proportional to SD:

5 $${{{\rm{SD}}}}\left({{{\rm{sites}}}}\, {{{{\rm{g}}}}}^{-1}\right)=\frac{{Q}_{{{{\rm{strip}}}}}\left({{{\rm{C}}}}\,{{{{\rm{g}}}}}^{-1}\right)\times {N}_{A}({sites}\, {{mol}}^{-1})}{{n}_{{{{\rm{strip}}}}}{{{\rm{F}}}}({{{\rm{C}}}}\,{{{{\rm{mol}}}}}^{-1})}$$

6 $${{{\rm{TOF}}}}\left({{{{\rm{s}}}}}^{-1}\right)=\frac{{n}_{{{{\rm{strip}}}}}\,{\Delta j}_{{{{\rm{k}}}}}\, ({{{\rm{mA}}}}\, {{{{\rm{cm}}}}}^{-2})}{{Q}_{{{{\rm{strip}}}}}\left({{{\rm{C}}}}\, {{{{\rm{g}}}}}^{-1}\right){L}_{{{{\rm{C}}}}}({{{\rm{mg}}}}\,{{{{\rm{cm}}}}}^{-2})}$$

where *n*_strip_ (=5) is the number of electrons transferred per nitrite stripped. *N*_A_ is Avogadro constant (6.02 × 10^23^ sites mol^−1^). F is Faraday’s constant (96485 C mol^−1^). *L*_C_ was the catalyst loading (0.242 mg cm^−2^). The catalysts were tested without further treatment.

### PEMFC tests

Membrane electrode assemblies (MEAs) were made using the hot-pressing method. The cathode ink was prepared by ultrasonically mixing the required amounts of Fe-NC catalysts, deionized water (0.2 mL), isopropanol (0.8 mL), and a 5 wt% Nafion solution in an ice bath for 1 h. This ink was applied to a gas diffusion layer (GDL, PTFE-pretreated Toray 060 carbon paper) with a loading of 3.5 mg cm^–2^. The Nafion content in the cathode layer was about 50 wt%. The anode catalyst was 40 wt.% Pt/C with a loading of 0.4 mg_Pt_ cm^-2^. The MEA was assembled by hot-pressing the cathode, anode, Nafion membrane (NRE 211), and a gasket at 135 °C and 3 MPa for 2 min. The active area of the MEA was and 2.1 × 2.1 cm^2^. Polarization curves were obtained at 80 °C using a Model 850e fuel cell test system (Scribner Associates, Inc.) in conjunction with an absolute pressure of 1.5 bar. The H_2_ and O_2_ (air) flow rates were 0.3 L min^–1^ and 0.4 L min^−1^ at 100% RH during measurements.

### Computational methods

All spin-polarized DFT computations were carried out with the VASP^37^. The PBE functional was applied in combination with the Van der Waals interaction^38,39^. The kinetic cut-off energy was 400 eV. The Fe_13_O_13_ cluster structure was firstly relaxed in the NVT ensemble at the temperature of 1170 K for 5 ps using the Nose-Hoover thermostat^40,41^. Accordingly, the structure of the Fe_13_O_13_ cluster on the N_4_-C was constructed, and the vacuum region was 12 Å at least between the periodic images. The whole structure was optimized by using a 2 × 2 × 1 k-point mesh in the Monkhorst-Pack scheme. All atoms were allowed to relax during the geometry optimization. The energy convergence criterion was 10^-5 ^eV, and the final force was less than 0.01 eV Å^−1^ at each atom. The nudged elastic band method was used to study the minimum energy path of the Fe atom transfer and the FeN_4_-C formation. The transition state was further searched by the dimer method^42,43^; the force convergence was 0.05 eV Å^−1^, and a single gamma point was applied. The molecular dynamics simulation was performed to study the reaction between the Fe_13_O_13_ cluster and the N_4_-C site leading to the FeN_4_-C formation at 1180 K. A time step of 0.5 fs was used, and the structure was preequilibrated for 0.5 ps before sampling. A single gamma point was applied in molecular dynamics simulations.

## Supplementary information

Supplementary Information

Peer Review File

Description of Additional Supplementary Files

Supplementary Movie 1

Supplementary Movie 2

Supplementary Movie 3

Supplementary Movie 4

Supplementary Data 1

## Source data

Source data

## Data Availability

The data that support the findings of this study are available within the article and its Supplementary Information files. All other relevant data supporting the findings of this study are available from the corresponding authors upon request. Source data are provided with this paper.
