# Supplementary material for: An in situ exploration of how Fe/N/C oxygen reduction catalysts evolve during synthesis under pyrolytic conditions
Source: Nat Commun. 2024 Jul 24;15:6229. doi: 10.1038/s41467-024-50629-x (PMC11266712; doi:10.1038/s41467-024-50629-x)
Supplement: Supplementary file 1 — Supplementary Information [file 41467_2024_50629_MOESM1_ESM.pdf]

Supplementary Information

An *in-situ* exploration of how Fe/N/C oxygen reduction catalysts evolve during synthesis under pyrolytic conditions

Shuhu Yin,[1] Hongyuan Yi,[1] Mengli Liu,[1] Jian Yang,[2] Shuangli Yang,[1] Bin-Wei Zhang,[2] Long Chen,[1] Xiaoyang Cheng,[1] Huan Huang,[2] Rui Huang,[3] Yanxia Jiang,[1,] Honggang Liao[1,*] and Shigang Sun[1]*

[1] State Key Laboratory of Physical Chemistry of Solid Surfaces, Engineering Research Center of Electrochemical Technologies of Ministry of Education, College of Chemistry and Chemical Engineering, and Discipline of Intelligent Instrument and Equipment, Xiamen University, Xiamen 361005, P. R. China

[2] Center of Advanced Electrochemical Energy, Institute of Advanced Interdisciplinary Studies, School of Chemistry and Chemical Engineering, Chongqing University, Chongqing 400044, PR China

[3] Beijing Synchrotron Radiation Facility, Institute of High Energy Physics, Chinese Academy of Sciences, Beijing 100049, P. R. China

*Corresponding authors:

Prof. Yanxia Jiang, E-mail: yxjiang@xmu.edu.cn

Prof. Honggang Liao, E-mail: hgliao@xmu.edu.cn

Supplementary Figures and Tables

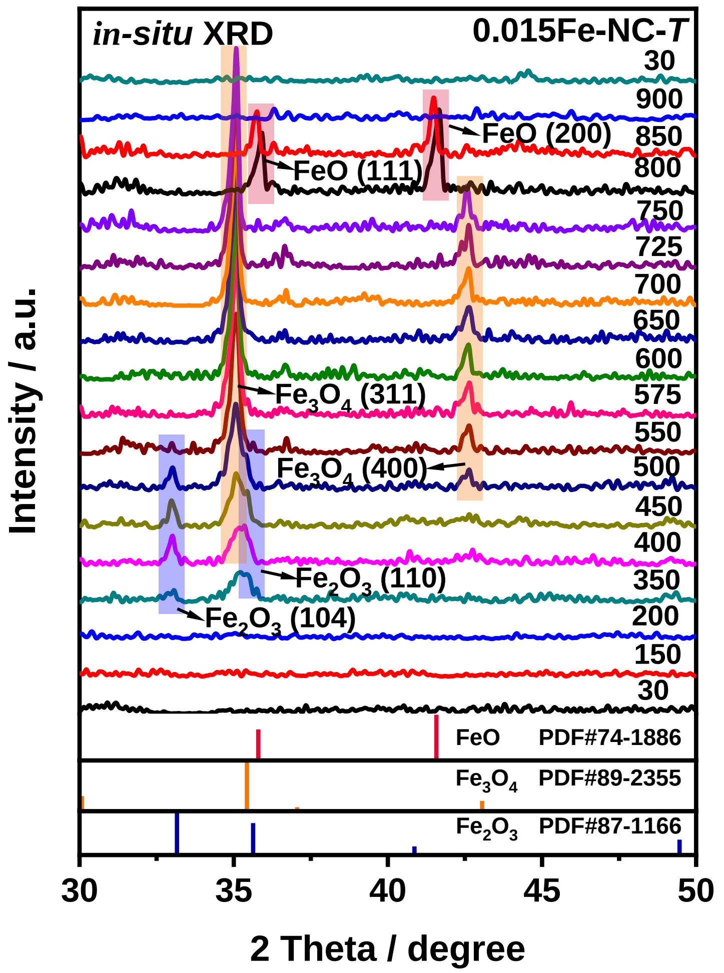

Supplementary Fig. 1 The XRD patterns of 0.015Fe-NC-*T* samples during *in-situ* heating XRD experiments.

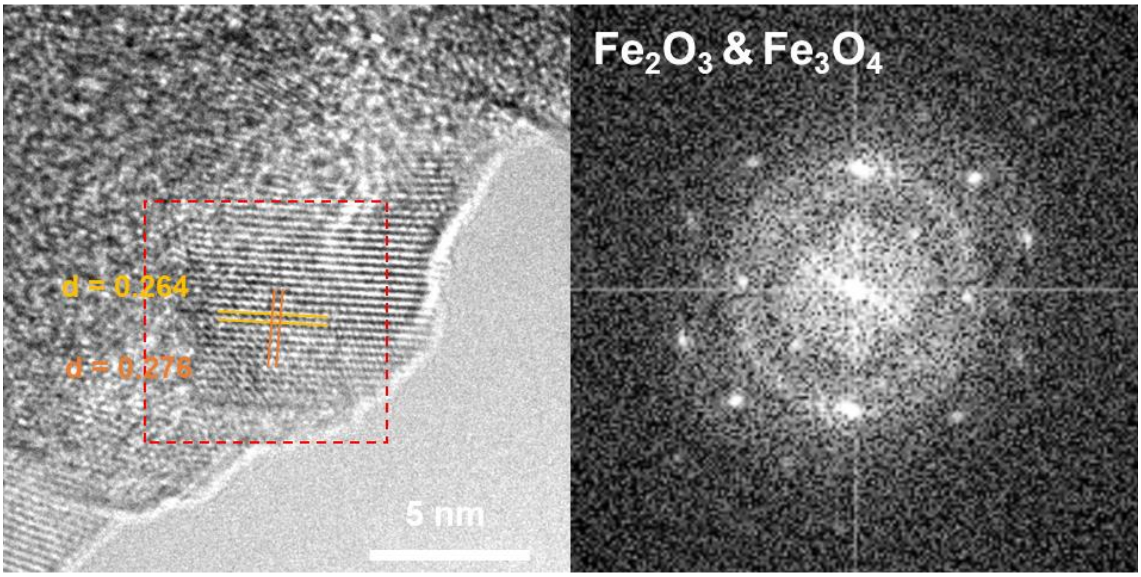

Supplementary Fig. 2 The corresponding fast Fourier transform (FFT) pattern of the nanoparticles under 500 °C during the *in-situ* heating TEM experiment.

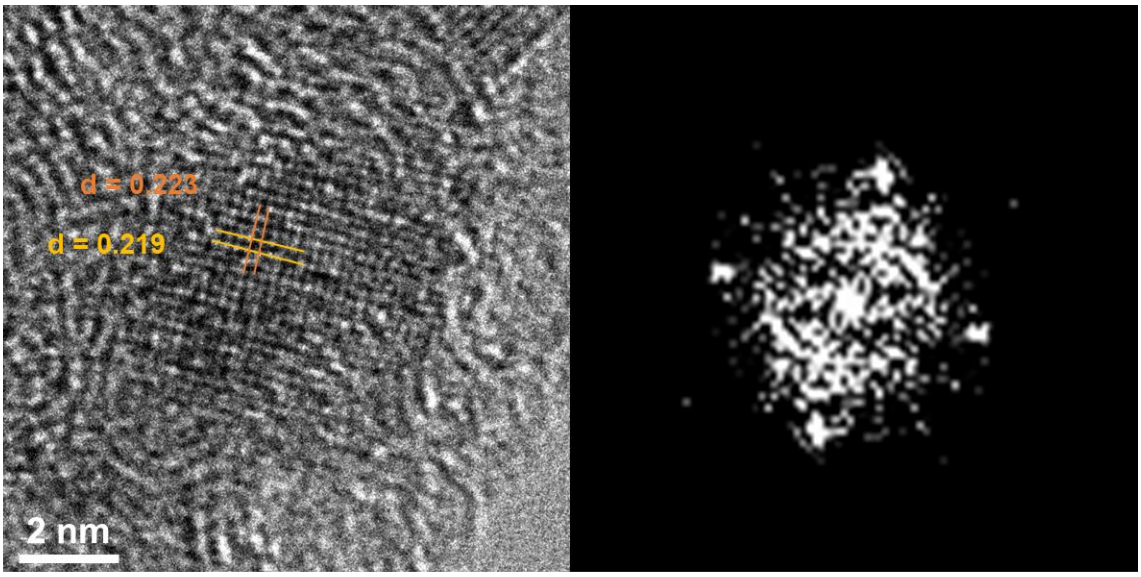

Supplementary Fig. 3 The corresponding fast Fourier transform (FFT) pattern of the nanoparticles under 800 °C during the *in-situ* heating TEM experiment.

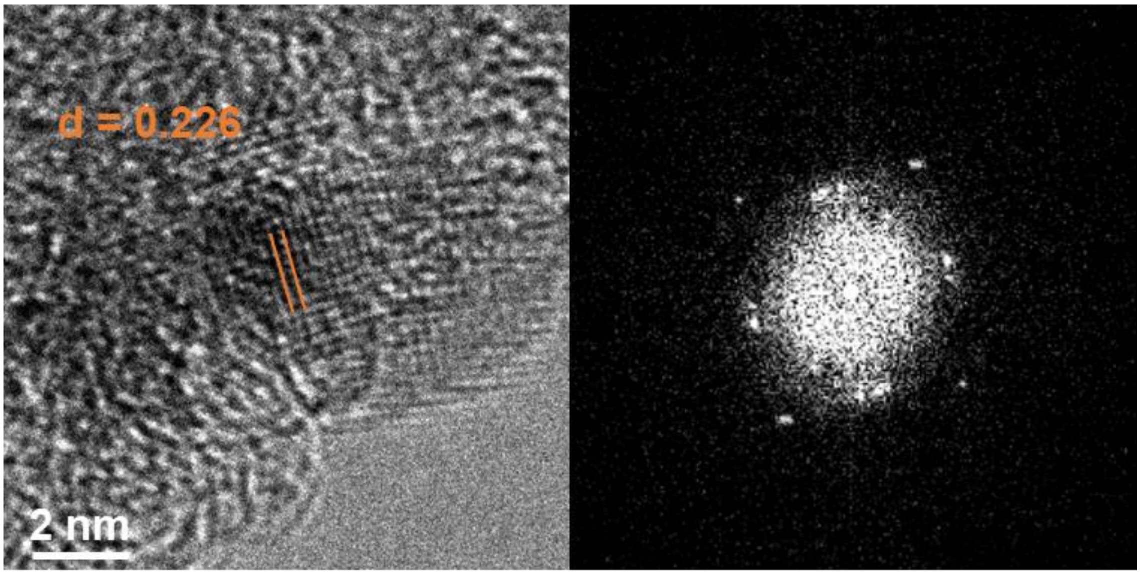

Supplementary Fig. 4 The corresponding fast Fourier transform (FFT) pattern of the nanoparticles under 1000 °C during the *in-situ* heating TEM experiment.

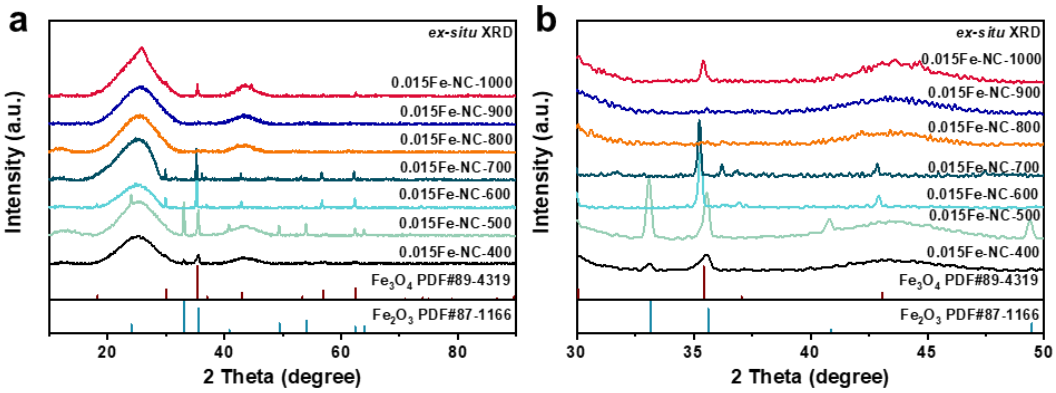

Supplementary Fig. 5 (a) *Ex-situ* XRD patterns of 0.015Fe-NC-*T* samples following thermal activation at varied temperatures. (b) Detailed enlargement of the selected region.

Upon elevating the temperature to 1000 °C, the characteristic peak of Fe_3O_4 emerges, signifying the oxidation of metallic Fe to Fe_3O_4 upon exposure to air.

Supplementary Notes

Note 1. Oxidative hydrolysis of $FeCl_2·4H_2O$ on the precursor at room temperature

To comprehend the rationale behind the formation of iron oxides during pyrolysis. We performed different characterization tests on 0.015Fe-NC-RT precursor. TEM was used to investigate the physical form of $FeCl_2·4H_2O$ present in the precursor. Analysis of the fresh 0.015Fe-NC-RT precursor TEM revealed a minimal degree of layered structure (**Supplementary Fig. 6**), which was confirmed by the distinct diffraction points present in the Fourier transform processing; thus, it can be deduced that the structure is crystalline rather than carbon substrate. Following the precursor's aging at room temperature for one month, a significant increase in the crystallinity of layered FeOOH was observed (**Supplementary Fig. 7**), and the selected area electron diffraction (SAED) revealed diffraction spots with a layer spacing of 0.35 nm. Besides, in FT-EXAFS of the precursor, two scattering peaks of 1.19 Å and 1.78 Å were observed (**Supplementary Figs. 28** and **29**), which were basically similar to the two Fe-O scattering peaks of FeOOH, while the Fe-Cl scattering peak was about 2.1 Å, which further indicated that $FeCl_2·4H_2O$ was oxidized and hydrolyzed to FeOOH at room temperature. We then fitted the near-edge absorption to determine the proportions of the various components by linear combination fitting (LCF). The analysis of the LCF results showed that the main component at room temperature was FeOOH (40.9%), with a minor amount of $FeCl_2·4H_2O$ and $FeCl_3$ (**Supplementary Fig. 32**, **Table 4**). It is generally accepted that $FeCl_2·4H_2O$ tends to easily deliquesce and be quickly oxidized to $FeCl_3$ when exposed to the atmosphere. This leads to the compound absorbing humidity from the air and undergoing hydrolysis. Initially, amorphous $Fe(OH)_3$ and a small quantity of layered FeOOH can be produced, however, as the hydrolysis intensifies, the crystallinity of FeOOH will become more evident. Therefore, the oxidative hydrolysis of Fe^{2+} in the fresh precursor is thought to result in the formation of Fe $(OH)_3$ or FeOOH. Additionally, $Fe(OH)_3$ or FeOOH is known to lose water and transform into iron oxide when heated above 350 °C, which is the impetus for the production of iron oxide during thermal activation. Validation experiments revealed that iron oxides were produced when $FeCl_3$ and FeAc underwent thermal activation (**Supplementary Fig. 8** and **9**). This is due to the hydrolysis of $FeCl_3$, and FeAc is decomposed into Fe_2O_3 by heating. In contrast, we

employ phenanthroline to form a strong complex with Fe^{2+} and create $[Fe(Phen)_3]^{2+}$ chelation, which prevents the oxidative hydrolysis of Fe^{2+}. Alternatively, we can use the iron phthalocyanine molecule as the iron source, and no iron oxide formation is observed during the thermal activation process (**Supplementary Fig. 10** and **11**). These results confirm that the formation of iron oxides is derived from the hydrolysis reaction of Fe^{2+} or Fe^{3+} at room temperature.

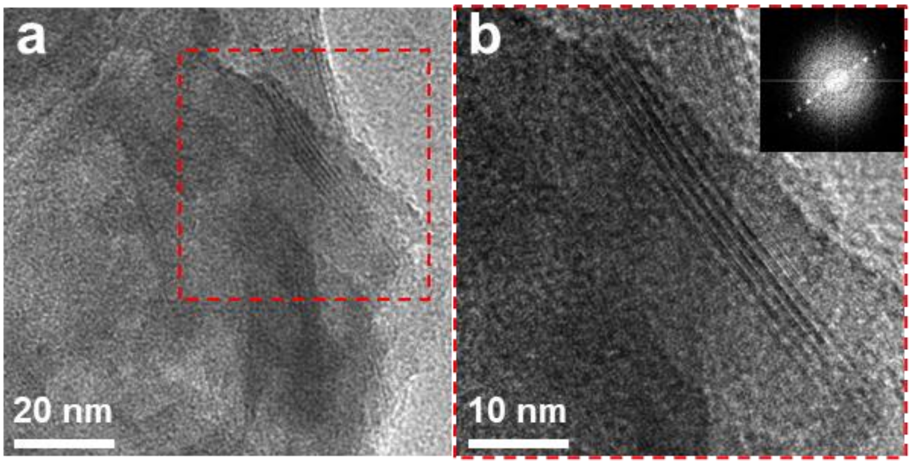

Supplementary Fig. 6 HR-TEM images of the fresh 0.015Fe-NC-RT precursor. The illustration is the corresponding fast Fourier transform (FFT) pattern.

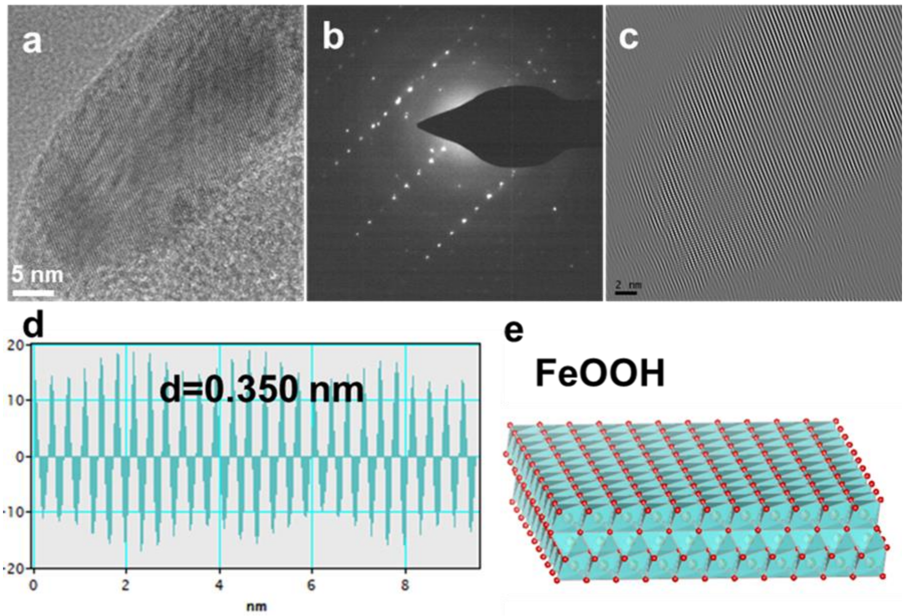

Supplementary Fig. 7 (a) HR-TEM images; (b) Selected area electron diffraction (SAED); (c) anti Fourier transform mode; (d) the layer spacing of the 0.015Fe-NC-RT precursor aging for a month; (e) the cif file of standard FeOOH.

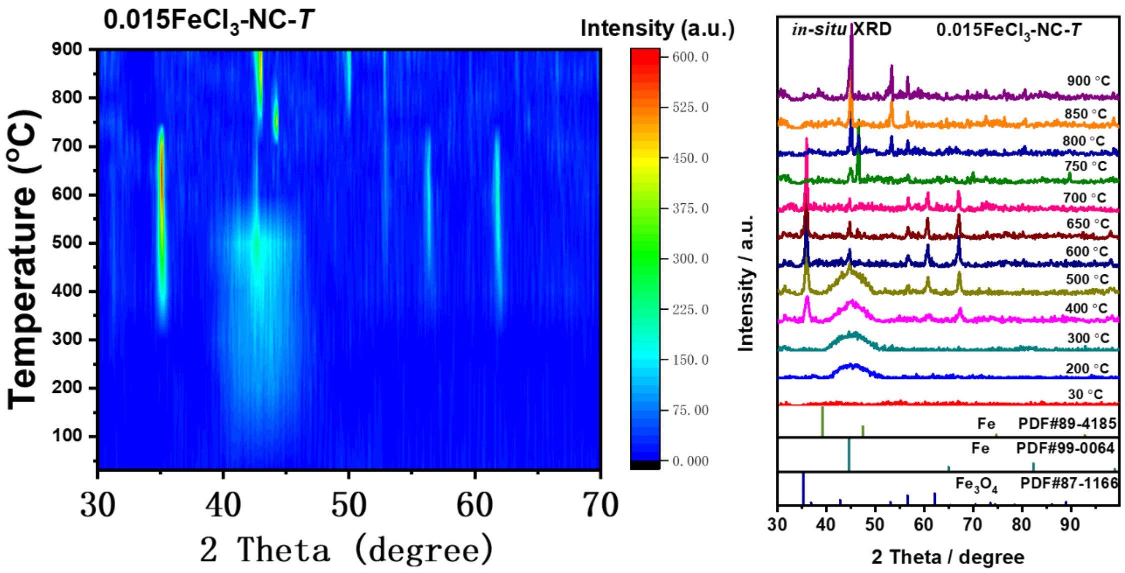

Supplementary Fig. 8 The in-situ heating XRD experiment of 0.015FeCl$_3$-NC-*T*.

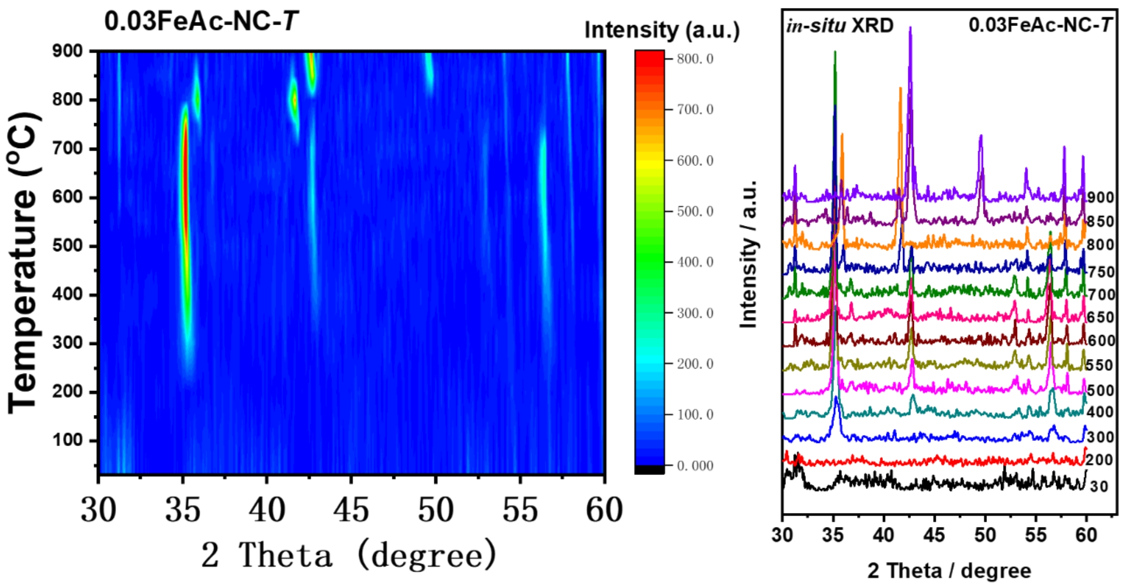

Supplementary Fig. 9 The in-situ heating XRD experiment of 0.03FeAc-NC-*T*.

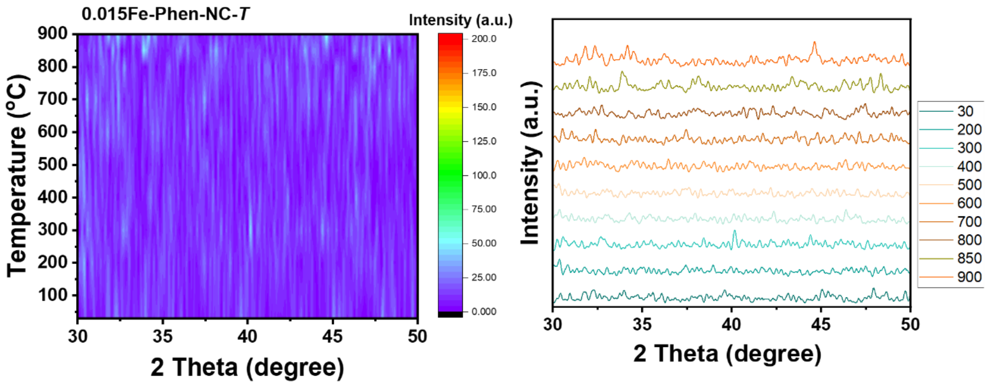

Supplementary Fig. 10 The in-situ heating XRD experiment of 0.015Fe-Phen-NC-*T*.

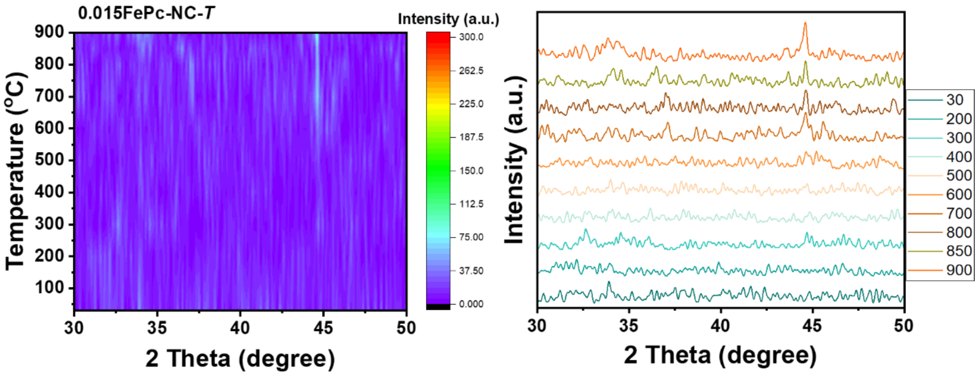

Supplementary Fig. 11 The in-situ heating XRD experiment of 0.015FePc-NC-*T*.

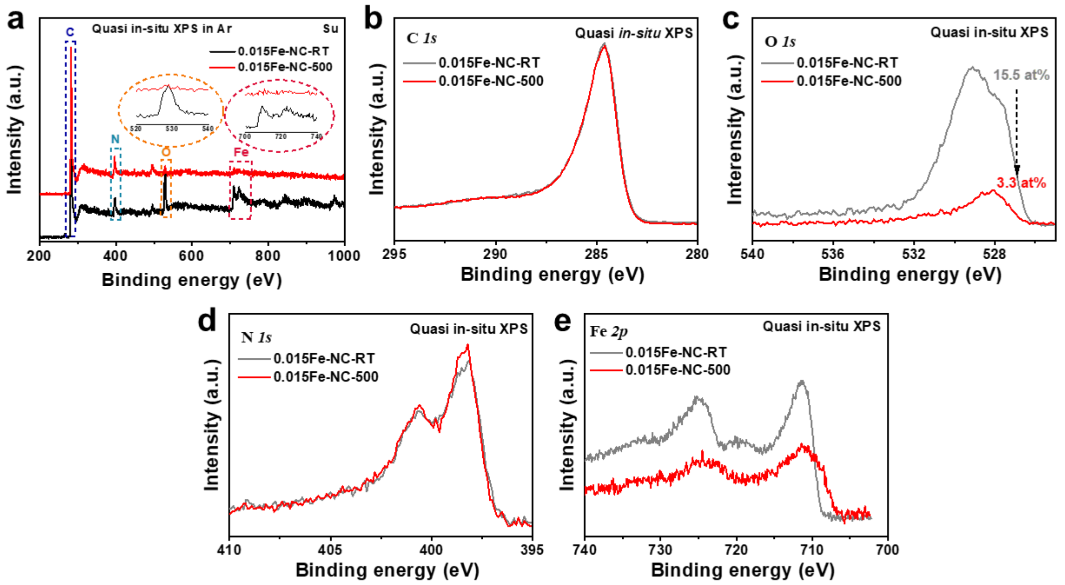

Supplementary Fig. 12 (a) Survey spectra; (b) C *1s* XPS spectra; (c) O *1s* XPS spectra; (d) N *1s* XPS spectra; (e) Fe *2p* XPS spectra of 0.015Fe-NC-RT and 0.015Fe-NC-500.

We further designed a quasi in-situ heating XPS experiment, which was heated to 500 ºC in an Ar atmosphere for ten minutes prior to being shifted to a vacuum chamber for testing. The spectral intensity of each element is normalized by C *1s* peak intensity. There is almost no change in the C *1s* spectrum. The changes in the O *1s* spectrum come from the removal of air and water adsorbed in the sample and the transition from $Fe(OH)_3$ to FeO_x. The slight change in the N *1s* spectrum may be due to the formation of a small amount of Fe-N_x.

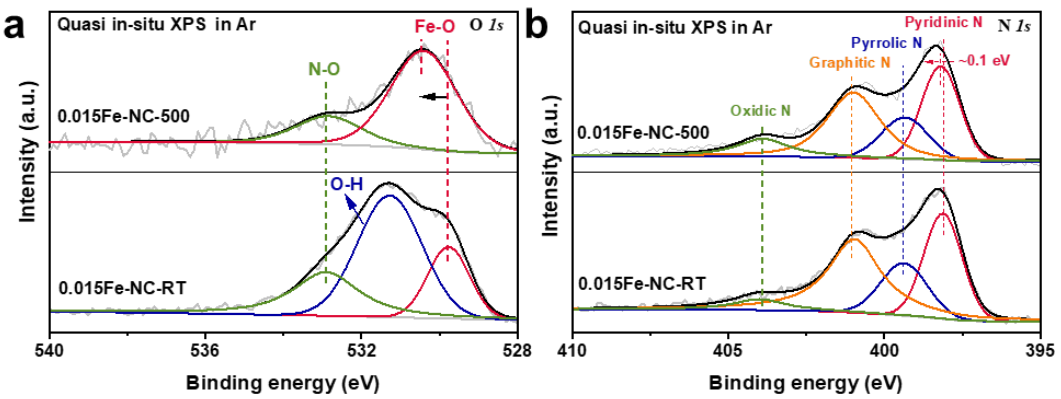

Supplementary Fig. 13 (a) XPS O *1s* fitting; (b) XPS N *1s* fitting.

The XPS O *1s* fine spectrum in **Supplementary Fig. 13a** indicates that, at room temperature, the oxygen element in the precursor is largely present as O-H bonds. This is likely due to the precursor adsorbing more water vapor in the air, while Fe-O bonds might be attributed to the Fe^{2+} oxidation hydrolysis product $Fe(OH)_3$ or FeOOH, which is in line with the earlier TEM results. After 10 minutes of thermal activation at 500 °C, it is evident that the water vapor evaporates, while the binding energy of the Fe-O bond increases, likely due to the dehydration of FeOOH into Fe_2O_3, and the oxygen element content decreases drastically throughout the thermal activation process. After 10 minutes of treatment at 500 °C, a positive shift of ~0.1 eV in the pyridine nitrogen was observed (**Supplementary Fig. 13b**), which may be due to the formation of Fe-N bonds in small quantities.

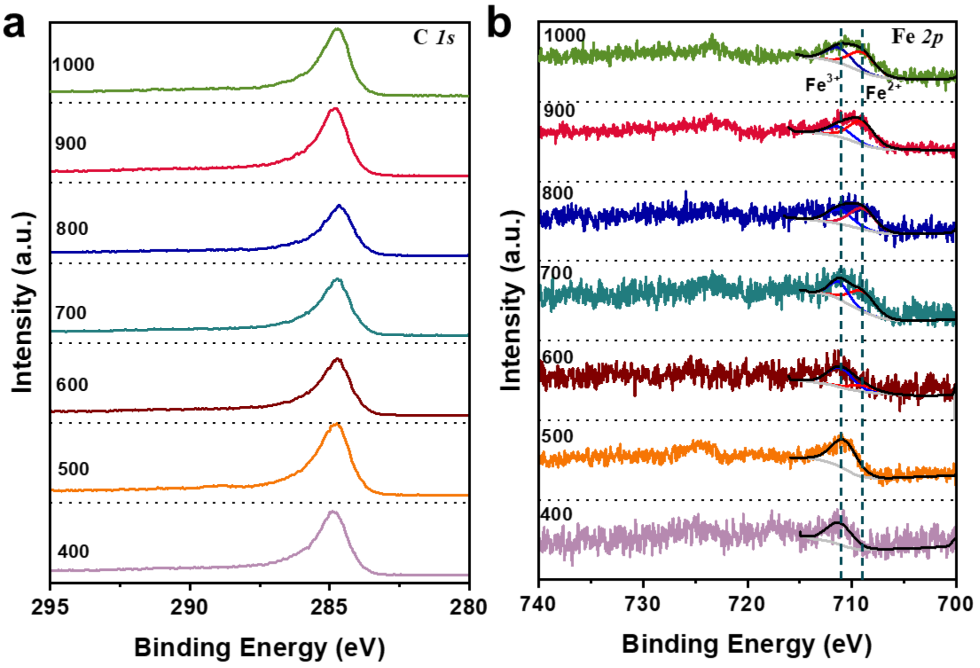

Supplementary Fig. 14 (a) XPS C *1s* spectra; (b) XPS Fe *2p* spectra.

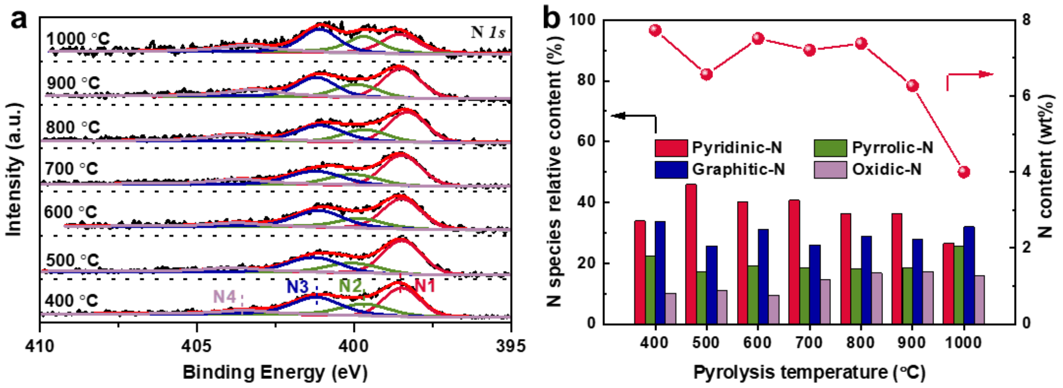

Supplementary Fig. 15 XPS analysis of 0.015Fe-NC-*T* samples. (a) XPS N *1s* spectra; (b) Fraction of various types of nitrogen and total N content.

Supplementary Table 1 Qusai in-situ XPS data for the surface Fe valence status of 0.015Fe-NC-500 samples.

Samples	Fe^{3+} / %	Fe^{2+} / %
	**711.00 eV**	**709.00 eV**
0.015Fe-NC-RT	100	0
0.015Fe-NC-500	66.27	33.73

Supplementary Table 2 XPS data for the surface Fe valence status of 0.015Fe-NC-*T* samples.

Samples	Fe^{3+} / %	Fe^{2+} / %
	**711.20 eV**	**709.00 eV**
0.015Fe-NC-400	100	0
0.015Fe-NC-500	100	0
0.015Fe-NC-600	75.76	24.24
0.015Fe-NC-700	46.24	53.76
0.015Fe-NC-800	40.83	59.17
0.015Fe-NC-900	30.56	69.44
0.015Fe-NC-1000	44.75	55.25

* The reason for the difference in Fe^{2+} content between quasi-in-situ and ex-situ XPS at 500 ºC is atmosphere protection. The ex-situ XPS sample is exposed to air, and the reduced Fe^{2+} is easy to adsorb oxygen or be oxidized to Fe^{3+}.

Supplementary Table 3. XPS data for the surface species for the bulk species of 0.015Fe-NC-*T* samples.

Samples	C / wt%	N / wt%	O / wt%	Fe / wt%	Zn / wt%
0.015Fe-NC-400	78.87	7.73	5.45	1.06	6.89
0.015Fe-NC-500	77.54	6.57	8.09	1.77	6.03
0.015Fe-NC-600	78.00	7.51	5.32	1.51	7.66
0.015Fe-NC-700	79.56	7.21	5.74	1.36	6.13
0.015Fe-NC-800	80.16	7.38	6.00	1.54	4.92
0.015Fe-NC-900	83.34	6.27	6.08	1.75	2.56
0.015Fe-NC-1000	87.43	4.00	6.59	1.57	0.42

The nanoparticle aggregation at 1000 °C stems from the depletion of N sites. The N content diminishes from approximately 7.5 wt% to around 4.0 wt%, as shown in **Supplementary Fig. 15** and **Table 3**. This suggests that the N component is lost at 1000 °C. Since Fe-N_x sites require an abundance of N sites to anchor Fe atom formation, the loss of N content leads to a weakened ability of the carbon support to secure Fe atoms, culminating in their aggregation into nanoparticles.

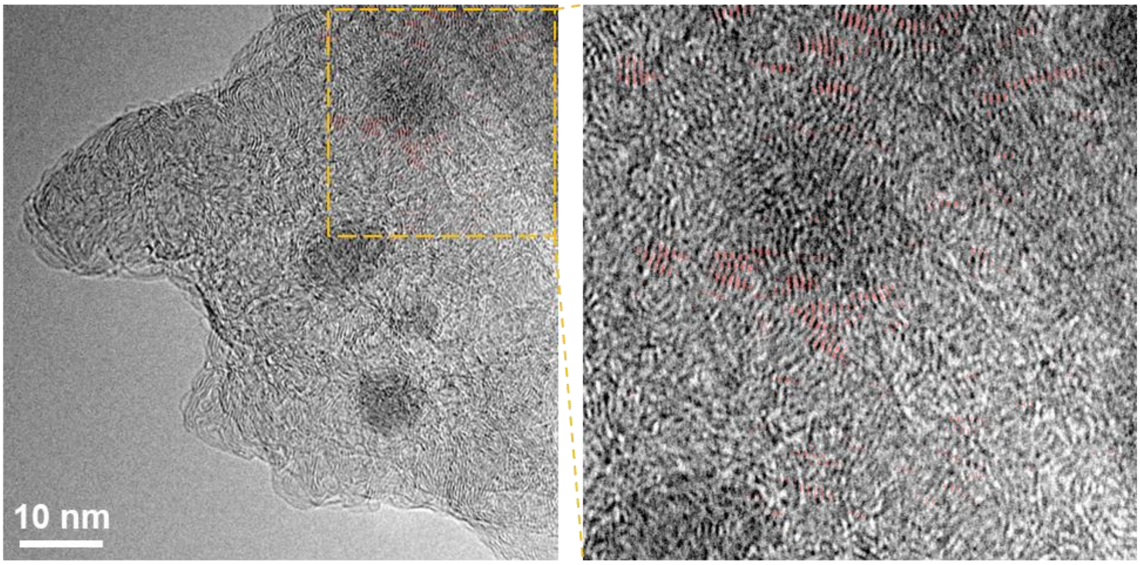

Supplementary Fig. 16 The original image of **Figure 2c** and a magnified region.

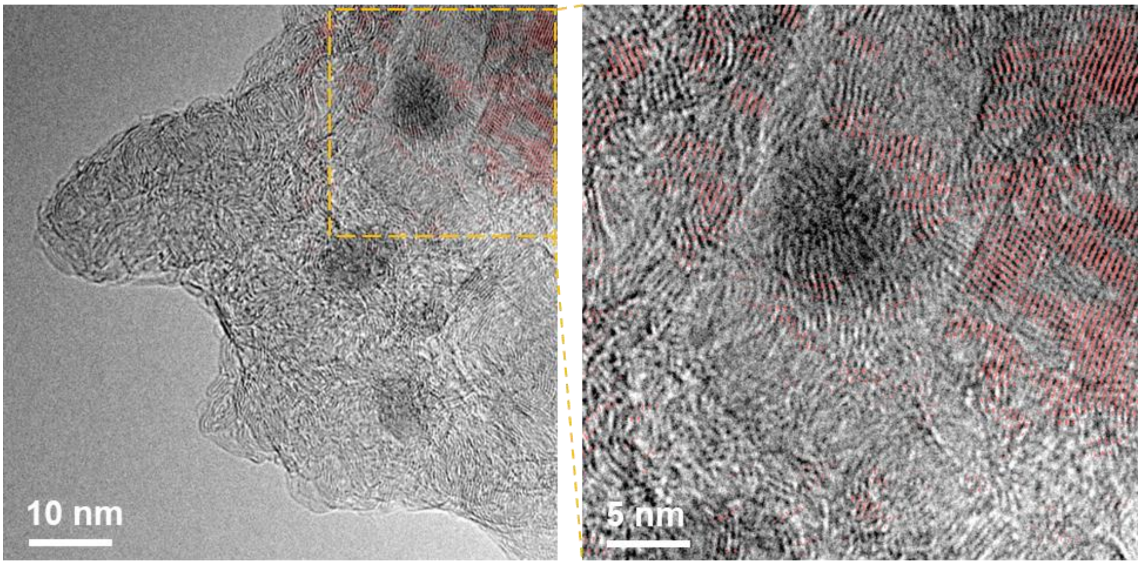

Supplementary Fig. 17 The original image of **Figure 2d** and a magnified region.

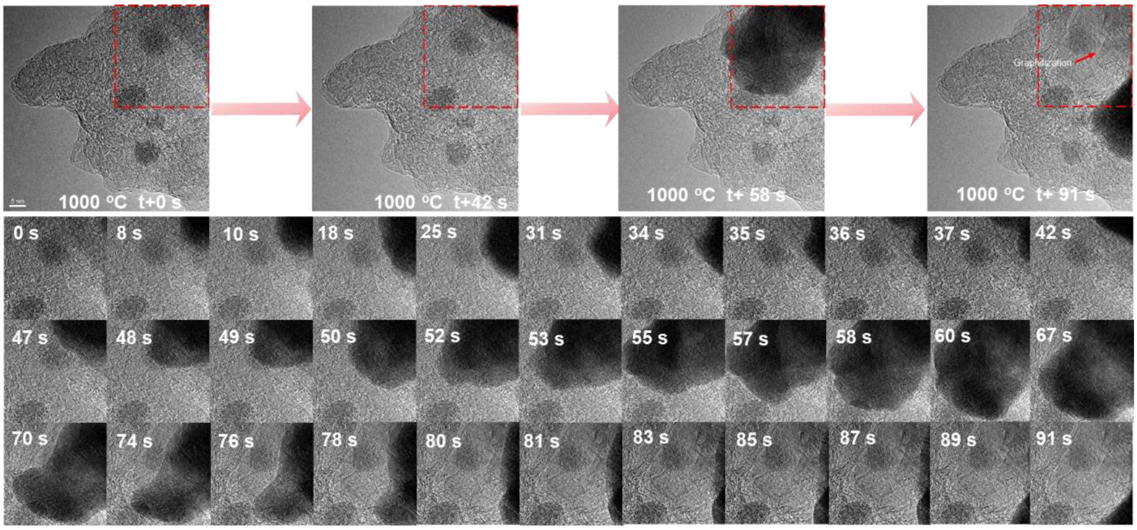

Supplementary Fig. 18 The diffusion migration of liquid nanoparticles during in-situ heating at 1000 ºC.

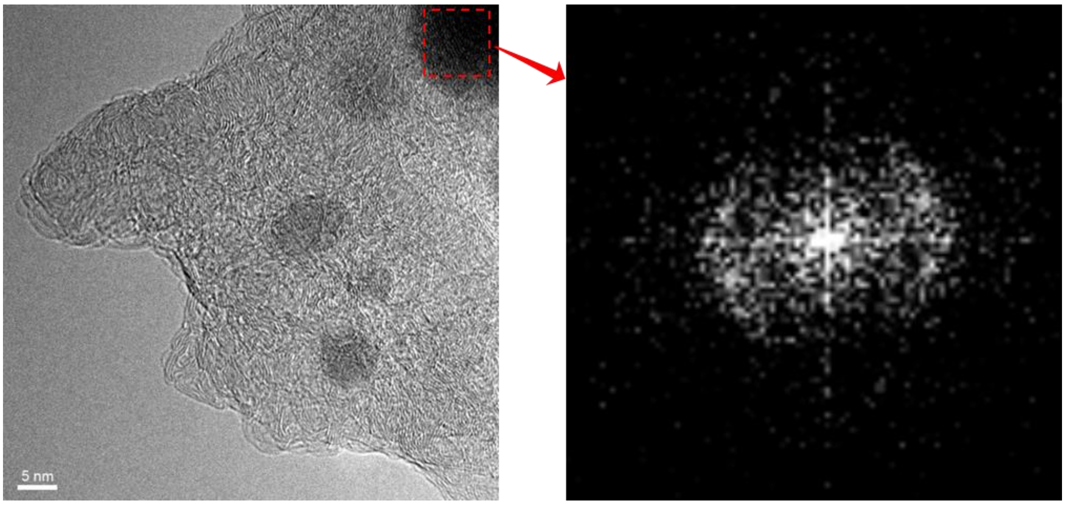

Supplementary Fig. 19 The corresponding fast Fourier transform (FFT) pattern of the moving nanoparticles.

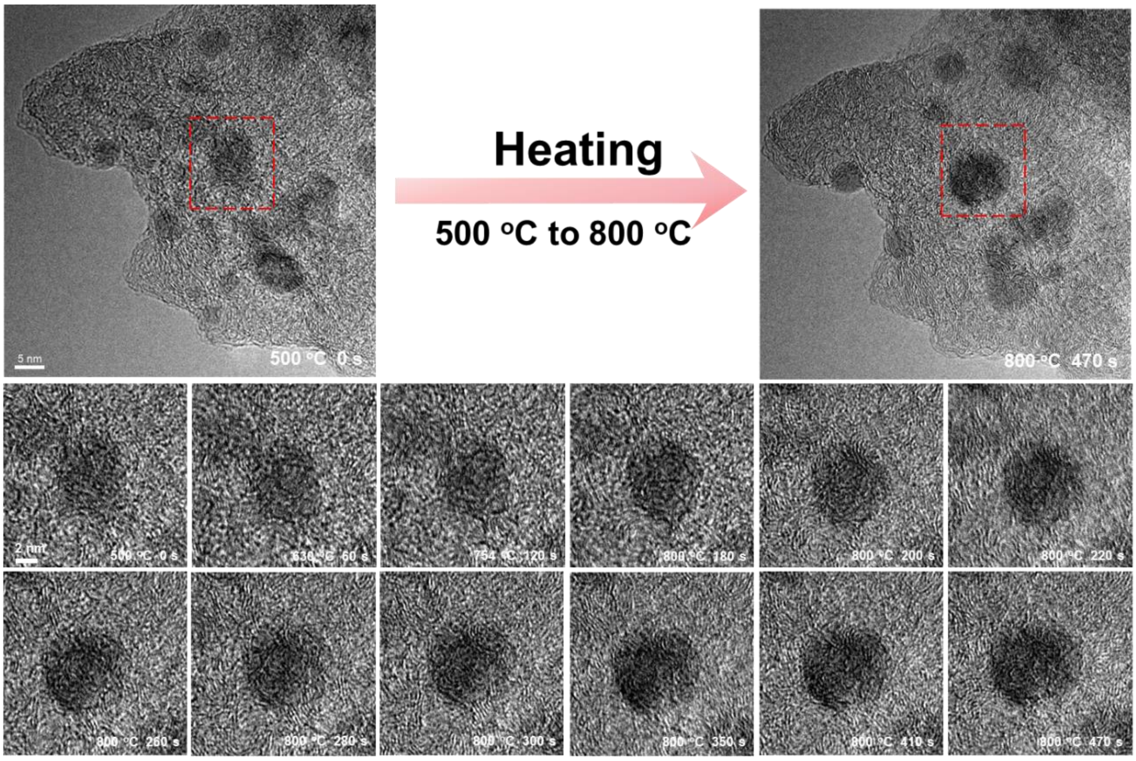

Supplementary Fig. 20 In-situ heating TEM experiments between 500 ºC to 800 ºC.

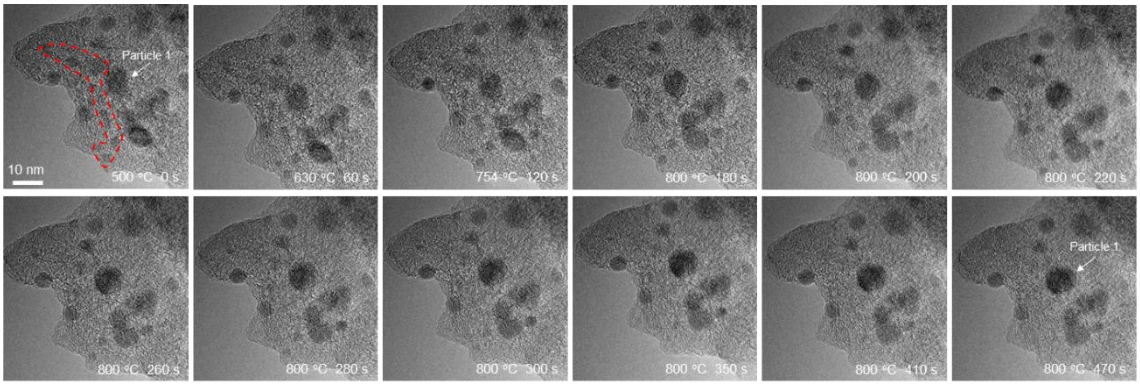

Supplementary Fig. 21 In-situ heating TEM experiments between 500 ºC to 800 ºC.

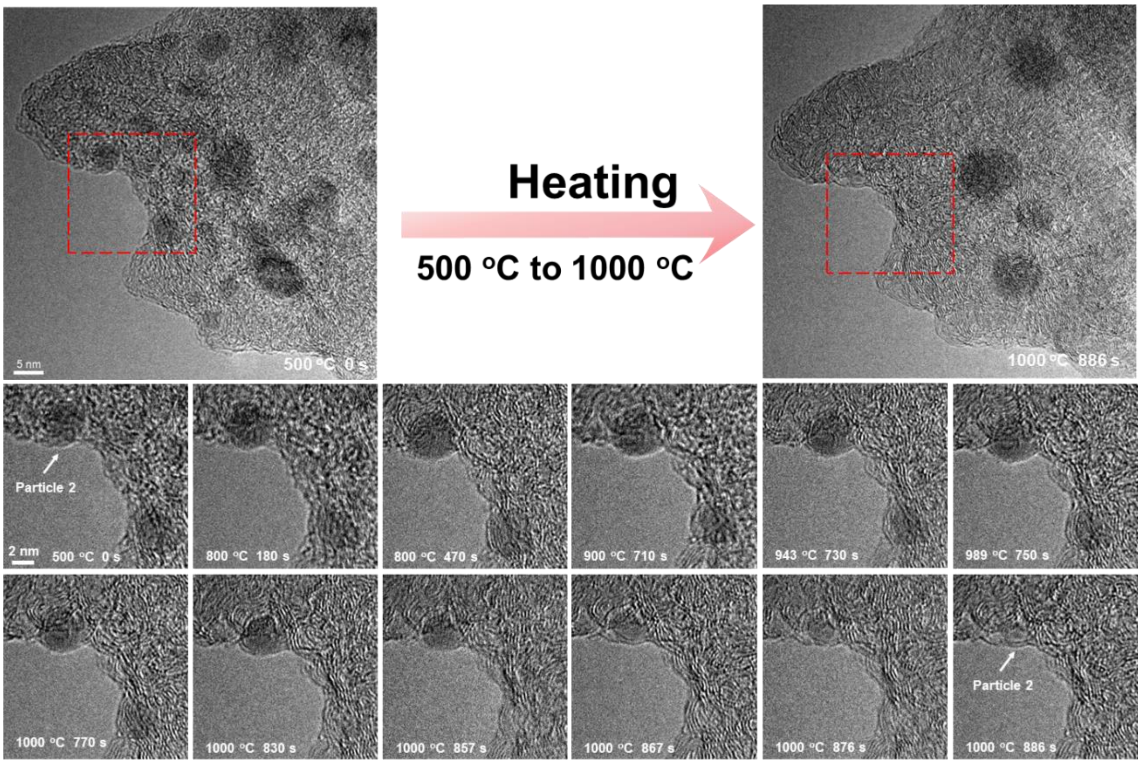

Supplementary Fig. 22 The geometric morphology of Particle 2 between 500 ºC to 1000 ºC during in-situ heating experiment.

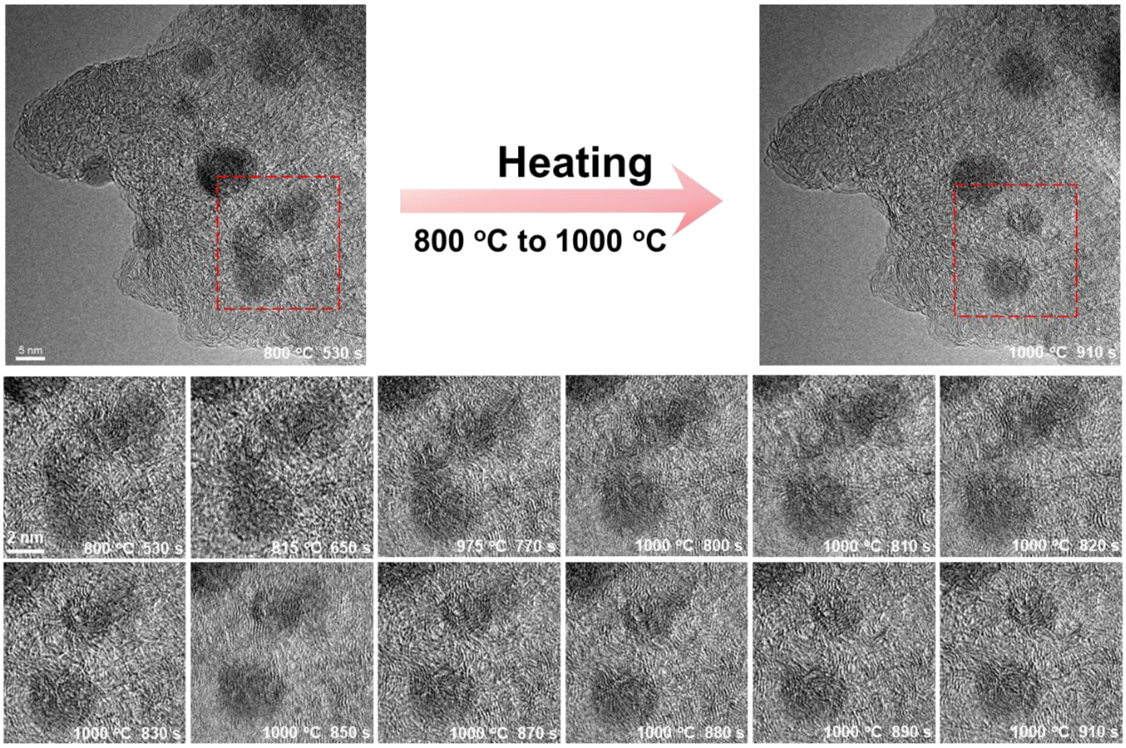

Supplementary Fig. 23 The geometric morphology of aggregated nanoparticles between 500 ºC to 1000 ºC during in-situ heating experiment.

Supplementary Notes

Note 2. The approach to handle in-situ TEM-captured videos

It is well-known that images or videos captured by transmission electron microscopes (TEM) often entail vast amounts of data, posing challenges for automated processing. For a video composed of tens of thousands of images, employing manual methods to periodically assess areas or contrasts typically requires counting every few dozen to a few hundred frames. Manual counting, however, confronts issues such as poor accuracy and tedious work. In this study, we applied a machine learning approach to handle in-situ TEM-captured videos. In this specific case, the grayscale differences between nanoparticles and the background were not distinctly apparent, prompting us to employ semantic segmentation to eliminate the randomness associated with manual counting.

To mitigate interference from large particles passing through the middle of the video, we divided it into two segments, conducting separate training for the semantic segmentation model. The first segment predicted four target particles and utilized 8,720 images after drift correction (Supplementary Movie 2), while the second segment predicted a single target particle and used 11,429 images (Supplementary Movie 3), focusing on the lower-left region unaffected by massive particles. After 100 rounds of model training for semantic segmentation, both segments reached optimal performance by the 80th round. Machine learning results proved to be highly accurate, with the model in the first segment achieving an MIOU (mean intersection over union) of 86.11 and a loss rate of 0.11. Notably, the second segment's trained model achieved an even higher MIOU of 90.01, accompanied by a low initial loss rate of 0.06. The training outcomes are presented in supplementary materials through a visualization video. Furthermore, we automated the conversion of all counted particle regions into changes in area and contrast using code, presenting the results in the form of charts.

Moreover, **Supplementary Fig. 24** showcases the evolution of the adjacent carbon layer within the orange virtual frame as the nanoparticles decompose. We note that initially, a single carbon layer encases the nanoparticles. After 30 seconds of treatment, a bi-layer forms, which then becomes a tri-layer after 60 seconds, while the nanoparticle size diminishes, indicative of decomposition. It is conceivable that the FeO_x nanoparticle surface may be reduced to metallic

Fe atoms. The amalgamation of these Fe atoms with carbon atoms on the surface results in the formation of carbon-rich and carbon-poor areas, prompting carbon migration toward the surface and the generation of an ordered carbon layer. Concurrently, Fe atoms diffuse outward, likely driven by local pressure disparities caused by the pyrolysis temperature and gases produced during the carbothermal reaction. During migration on the carbon substrate, these iron atoms readily bind to nitrogen sites, forming Fe-N_x sites (**Supplementary Fig. 25**).

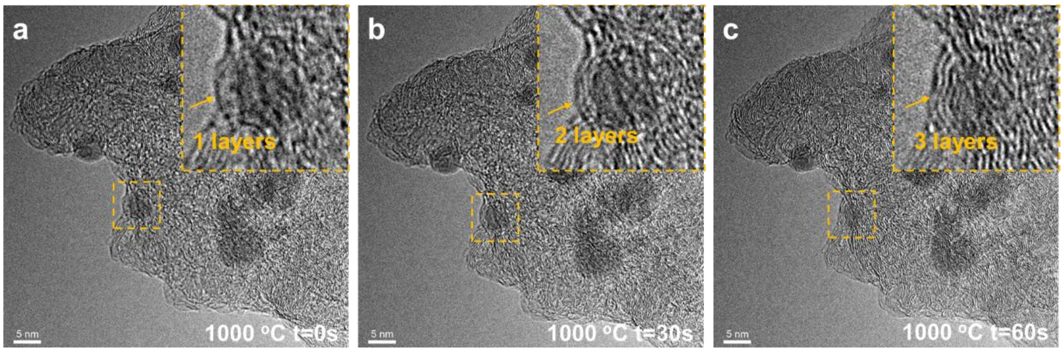

Supplementary Fig. 24 The change of nanoparticles adjacent to the carbon layer.

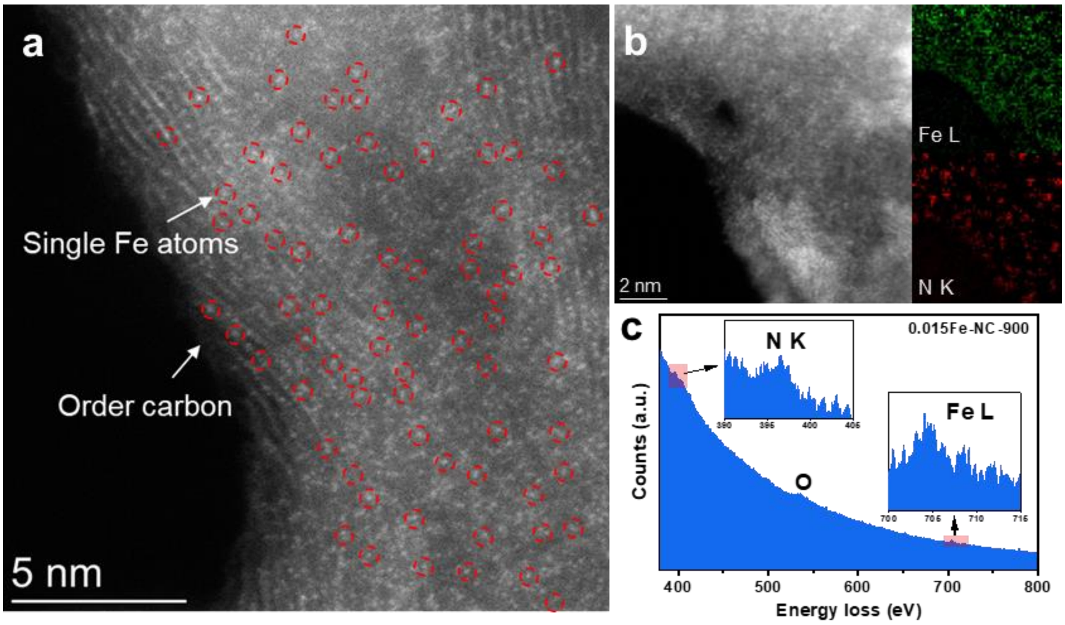

Supplementary Fig. 25 (a) The AC-HAADF-STEM image; (b) HAADF-STEM image and EDS elemental maps; (c) corresponding EEL point spectra of the 0.015Fe-NC-900.

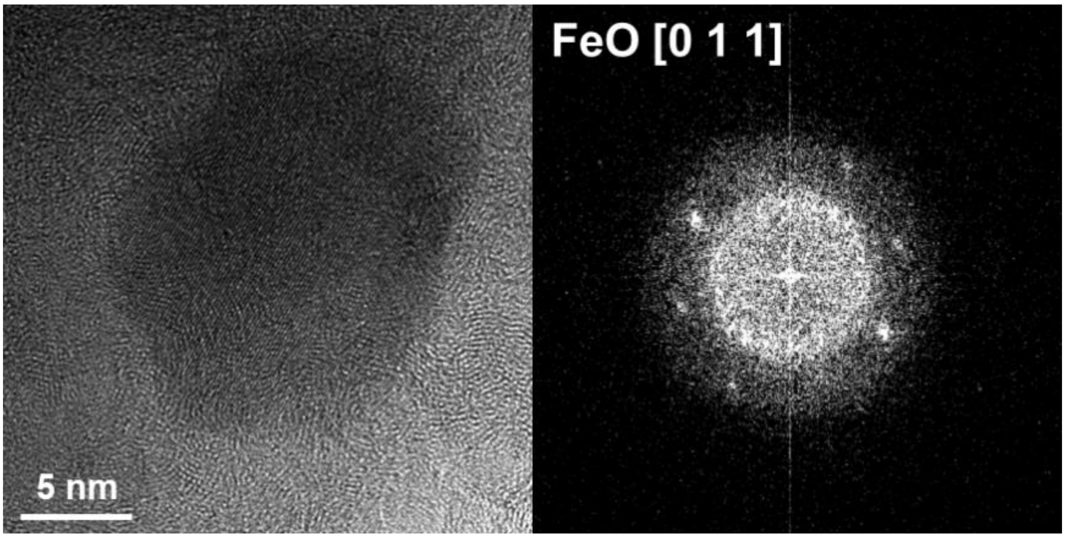

Supplementary Fig. 26 The corresponding fast Fourier transform (FFT) pattern of the ultra-large nanoparticles after stopping movement.

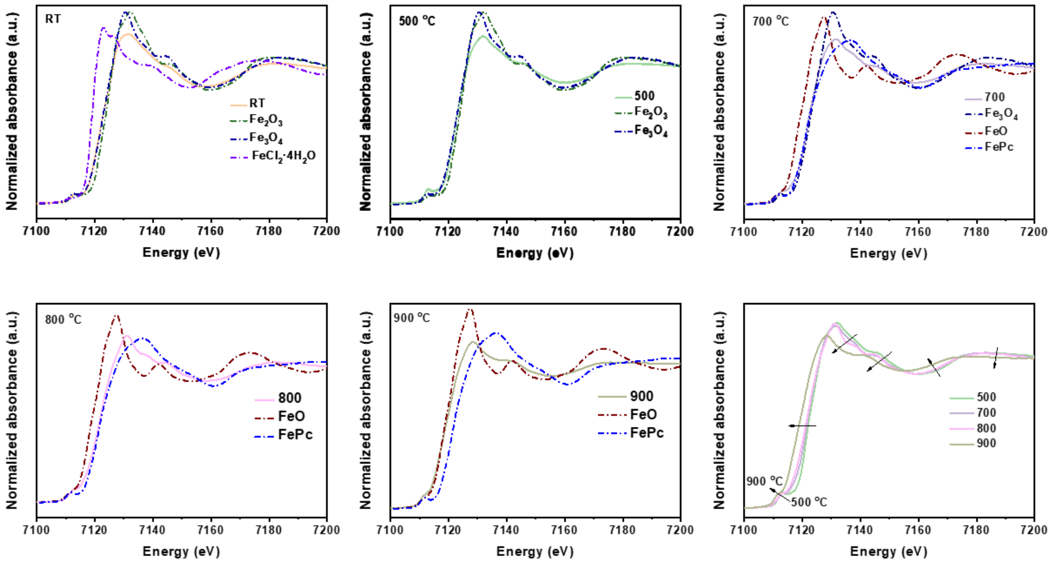

Supplementary Fig. 27 Fe K-edge XANES of 0.015Fe-NC-*T*.

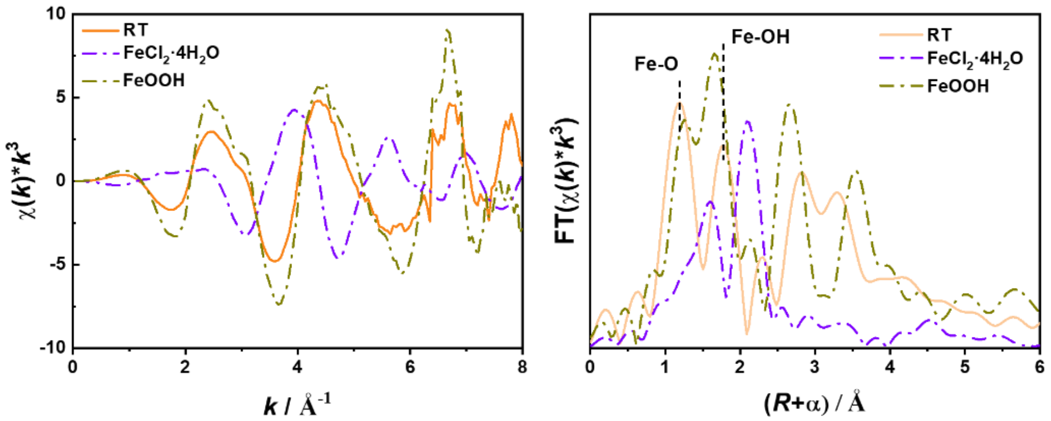

Supplementary Fig. 28 Fe K-edge FT-EXAFS of 0.015Fe-NC-RT, $FeCl_2{\cdot}4H_2O$ and FeOOH.

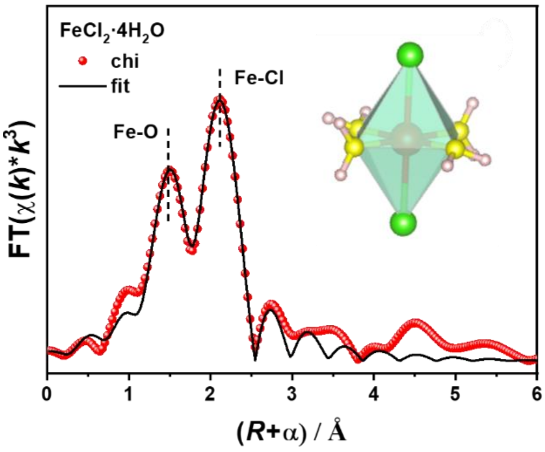

Supplementary Fig. 29 Fe K-edge EXAFS-fitting of $FeCl_2{\cdot}4H_2O$.

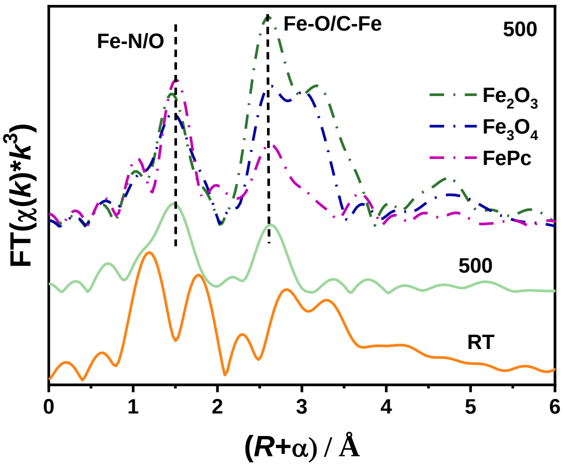

Supplementary Fig. 30 Fe K-edge EXAFS of 0.015Fe-NC-RT, 0.015Fe-NC-500, Fe_2O_3, Fe_3O_4 and FePc.

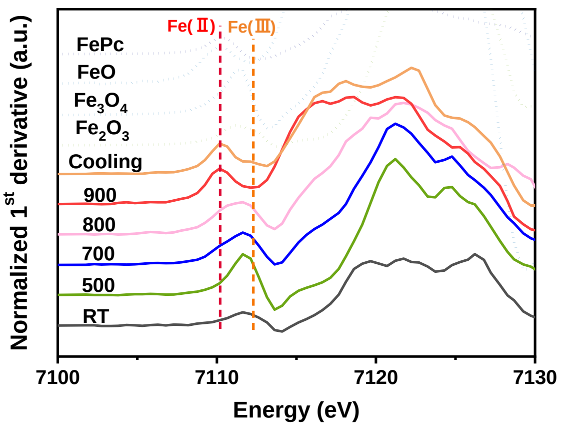

Supplementary Fig. 31 The 1st derivative of the normalized XANES data for the 0.015Fe-NC-*T* during in-situ heating XAS experiment.

Supplementary Notes

Note 3. Linear combination fitting (LCF) of 0.015Fe-N-C-*T* during pyrolysis

We fitted the near-edge absorption to determine the proportions of the various components by linear combination fitting (LCF) (**Supplementary Fig. 32, Table 4** and **5**). We attempted to fit using a variety of standard samples, including $FeCl_2$-4H_2O, $FeCl_3$, FeOOH, FePc, and Fe_2O_3. After testing different combinations, we found that the combination of FeOOH (40.9%), $FeCl_2\cdot 4H_2O$ (22.4%), $FeCl_3$ (21.6%), and FePc (15.1%) had the best fitting effect, with a R factor of only 1.3%. The main component of the precursor is FeOOH, which is in agreement with the results of the previous analysis, and this further confirms that the oxidation hydrolysis product is present. Due to the dehydration of FeOOH above 500 ºC, which resulted in the formation of FeO_x. Thus, the samples treated at 500 ºC, 700 ºC, 800 ºC, and 900 ºC were fitted with Fe_2O_3, Fe_3O_4, FeO, $FeCl_2$, Fe foil and FePc until the best fitting effect was achieved, with all R factors being less than 1%. The square-planar Fe–N_4 configuration of FePc is consistent with the Fe–N_4 configuration of pyrolytic Fe/N/C. It is assumed that the FePc component is the Fe–N_4 sites component that is generated during pyrolysis. The content of Fe_2O_3 decreased with increasing temperature, vanishing at 700 °C; the content of Fe_3O_4 increased initially, peaked at 700 °C, and then vanished completely at 900 °C; the content of FeO and FePc increased gradually (**Figure 4b**). At a temperature of 900 °C, the Fe species that have not fully converted predominantly exist as FeO and Fe foil, with the Fe-N_4 active sites constituting only 44.1% of the total.

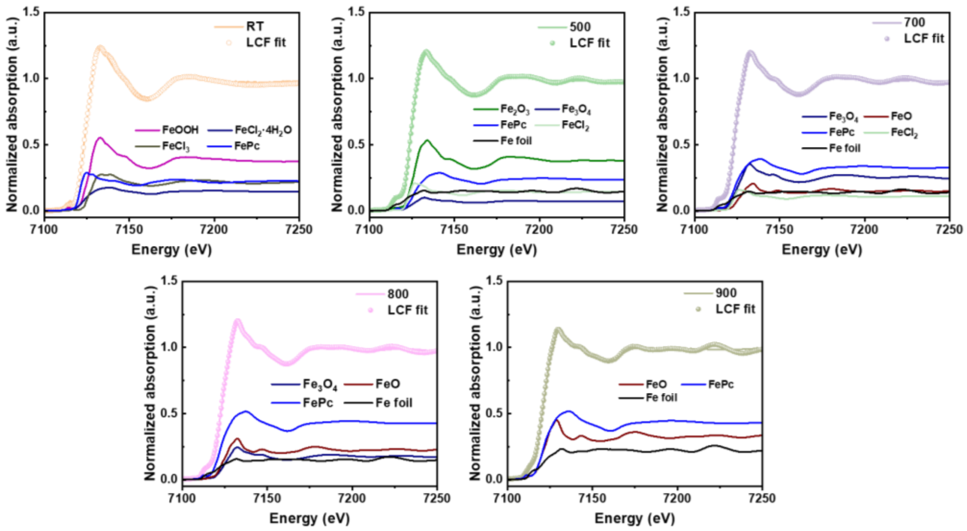

Supplementary Fig. 32 LCF fitting results of 0.015Fe-NC-*T*.

Supplementary Table 4 LCF result of 0.015Fe-NC-RT.

Content / Sample	FeOOH(%)	$FeCl_2·4H_2O$(%)	$FeCl_3$(%)	FePc(%)	R-factor
0.015Fe-NC-RT	40.9	22.4	21.6	15.1	0.0013

Supplementary Table 5 LCF result of 0.015Fe-NC-*T*.

Content / Samples	FeO (%)	Fe_3O_4 (%)	Fe_2O_3 (%)	FePc (%)	Fe foil (%)	$FeCl_2$ (%)	R-factor
0.015FeNC-500	0	7.3	38.7	24.5	15.1	14.5	0.0003
0.015FeNC -700	15.1	25.9	0	33.4	14.5	11.0	0.0002
0.015FeNC -800	22.7	17.9	0	43.9	15.4	0	0.0005
0.015FeNC -900	33.0	0	0	44.1	22.9	0	0.0006

Supplementary Table 6 EXAFS fitting parameters at the Fe K-edge various samples (S_0^2=0.792).

Samples	Shell	CN^a	R(Å)^b	σ^2(Å^2)^c	ΔE_0(eV)^d	R factor
Fe foil	Fe-Fe	8	2.46	0.0045	5.0	0.0017
	Fe-Fe	6	2.85	0.0045		
FeO	Fe-O	6.0	2.12	0.0054	-1.4	0.0009
	Fe-Fe	11.7	3.07	0.0139		
Fe_2O_3	Fe-O	6.0	2.04	0.0097	0.1	0.0009
	Fe-Fe	5.9	2.97	0.0073		
	Fe-Fe	4.2	3.39	0.0073		
	Fe-Fe	5.6	3.70	0.0073		
Fe_3O_4	Fe-O	6.0	1.98	0.0088	-1.1	0.0009
	Fe-Fe	4.5	2.97	0.0106		
	Fe-Fe	10.8	3.47	0.0106		
FePc	Fe-N	4.0	1.96	0.0036	1.2	0.0015
0.015Fe-NC-500	Fe-N/O	5.7±0.5	1.98	0.0139	0.2	0.0001
	Fe-Fe	3.3	2.95	0.0142		
0.015Fe-NC-700	Fe-N/O	5.1±0.4	1.99	0.0117	1.1	0.0002
	Fe-Fe	4.2	2.97	0.0152		
0.015Fe-NC-800	Fe-N/O	4.9±0.6	2.01	0.0210	-1.0	0.0003
	Fe-Fe	4.4	2.99	0.0165		
0.015Fe-NC-900	Fe-N/O	4.5±0.7	2.00	0.0306	-1.7	0.0007
	Fe-Fe	2.4	2.97	0.0199		
	Fe-Fe	3.3	3.81	0.0199		

^{a}*CN*: coordination numbers; ^{b}*R*: bond distance; $^c\sigma^2$: Debye-Waller factors; ^{d} ΔE_0: the inner potential correction. *R* factor: goodness of fit. S_0^2 was fixed at 0.792, according to the experimental EXAFS fit of Fe foil by fixing CN as the known crystallographic value.

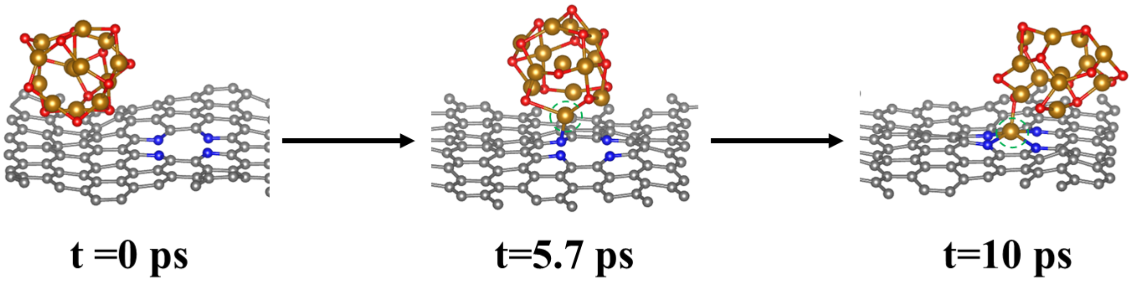

Supplementary Fig. 33 Time-series images during $Fe_{13}O_{13}$ to Fe-N_4 site from **Supplementary Movie 4**.

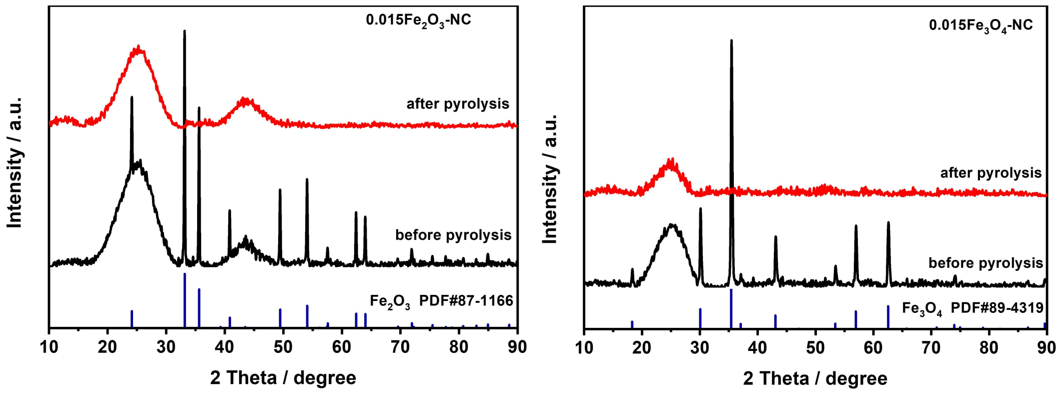

Supplementary Fig. 34 XRD of 0.015Fe_2O_3-NC and 0.015Fe_3O_4-NC before and after pyrolysis.

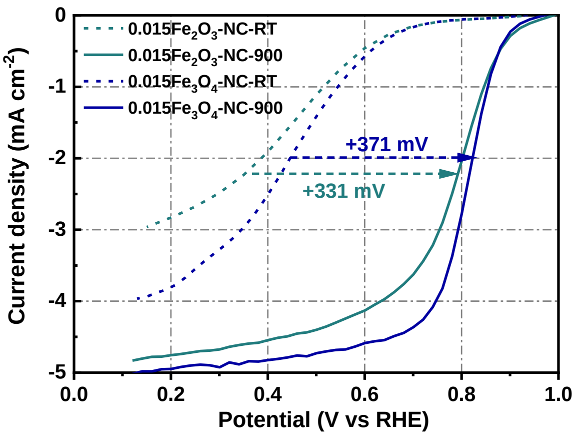

Supplementary Fig. 35 ORR performance under 0.1 M H_2SO_4 of 0.015Fe_2O_3-NC-900 and 0.015Fe_3O_4-NC-900 before and after pyrolysis. The potential has been corrected by IR, and the resistance is automatically compensated by 80 %.

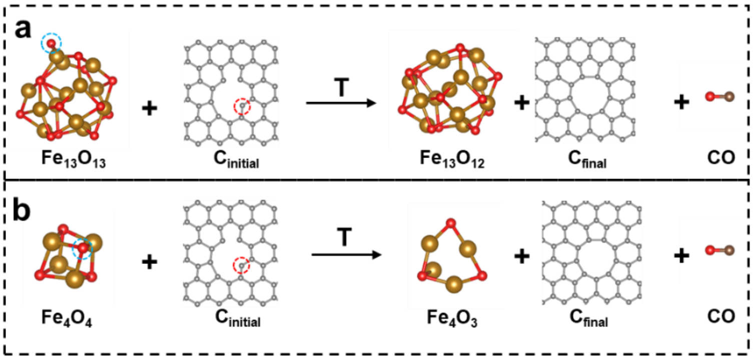

Supplementary Fig. 36 A schematic diagram of the reaction of Fe_nO_n with different atomic numbers with C to form CO, the equation: $Fe_nO_n + C_{initial} \rightarrow Fe_nO_{n-1} + C_{final} + CO$. (a) $Fe_{13}O_{13}$, (b) Fe_4O_4.

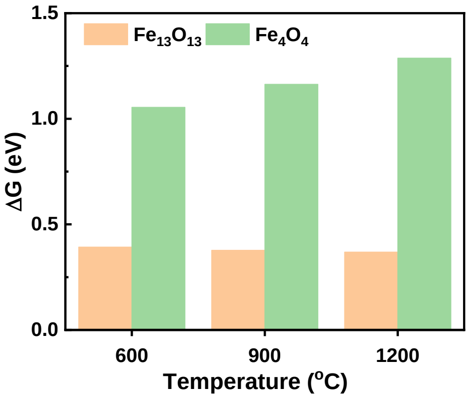

Supplementary Fig. 37 The Gibbs free energy changes (Δ*G*) of two reactions at different temperatures.

The thermodynamic Gibbs free energy change (Δ*G*) for the reaction between FenOn with varying atomic numbers and carbon to form CO at different temperatures. As shown in Supplementary Fig. 36, we fixed n at 4 and 13 to simulate different sizes of FeO_x nanoparticles. The carbon atom at the defect site reacts with an oxygen atom from Fe_nO_n, breaking the Fe-O bond and generating CO and Fe_nO_{n-1}. Interestingly, the reaction involving Fe_4O_4 clusters requires a higher Δ*G*, likely because the small number of atoms makes it difficult to break the Fe-O bond (Supplementary Fig. 37). Moreover, the Δ*G* values for the reaction at different temperatures are essentially the same. This indicates that the pyrolysis temperature does not overcome the thermodynamic barrier of the conversion reaction but rather accelerates the reaction kinetics, thereby increasing the conversion rate of FeO_x to $Fe-N_4$ sites.

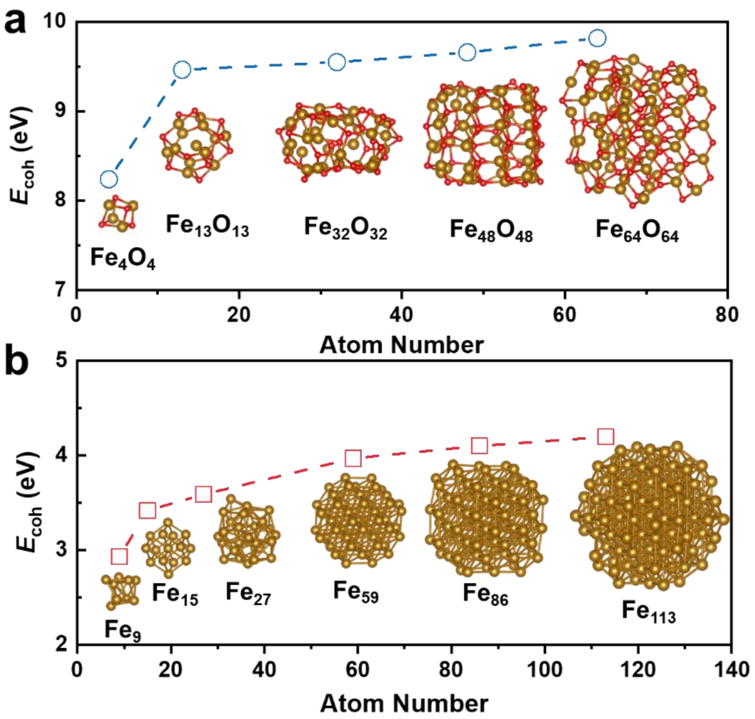

Supplementary Fig. 38 The cohesive energy of model molecules with different atomic numbers. (a) Fe_nO_n models, (b) Fe_n models.

The release of a Fe atom from the nanoparticles involves the structural stability of the nanoparticles themselves. A very important metric for stability of nanoparticle is the cohesive energy (E_{coh}), which can be written as

$$E_{coh} = |E_{FenOn}/n - (E_{Fe} + E_O)| \quad (1)$$

$$E_{coh} = |E_{Fen}/n - E_{Fe}| \quad (2)$$

where E_{FenOn} and E_{Fen} are the electronic energies of the Fe_nO_n and Fe_n nanoparticles, respectively, E_{Fe} and E_O are energies of free Fe and O atoms, respectively. In this convention, a lower cohesive energy indicates a weaker cohesion of the nanoparticle, i.e. thermal decomposition. Obvious, whether Fe_nO_n or Fe_n models (n denotes the number of atoms), the E_{coh} increases with the increase of n, indicating that the separation of nanoparticles into single atoms requires greater energy (Supplementary Fig. 38). This is also consistent with our experimental results, that is, we observed that nanoparticles less than 7 nm were completely decomposed, forming Fe-N_x sites or sintered into larger nanoparticles by Ostwald ripening. The nanoparticles larger than 10 nm are not completely decomposed and tend to be sintered to form larger nanoparticles.

Supplementary Notes

Note 4. Active Fe–N_4 sites of 0.015Fe-N-C-*T* during pyrolysis

By pyrolyzing precursors at varying thermal activation temperatures, we are able to produce Fe-N-C materials with different number of active Fe–N_4 sites. We then investigate the correlation between material structure and catalytic performance. By employing the nitrite poisoning experiment, we quantitatively determined the active Fe–N_4 site density of materials at varying thermal activation temperatures. To eliminate the interference of iron oxide, Fe_2O_3-NC material was initially employed in the nitrite poisoning experiment (**Supplementary Fig. 39**). The results were in agreement with those reported in the literature, indicating that Fe_2O_3 or other metal-free sites had a minimal impact on nitrite. To further analyze the pyrolysis temperature and conversion rate of Fe–N_4 sites, we take the FePc component content from LCF-fitting results to be the bulk Fe–N_4 sites, subtracting the number of active Fe–N_4 sites (obtained from nitrite poisoning experiment) to obtain the number of inactive Fe–N_4 sites. The Fe weight content obtained by ICP-MS can be converted into the total number of Fe atoms. The results of the calculation are outlined in **Supplementary Table 9**.

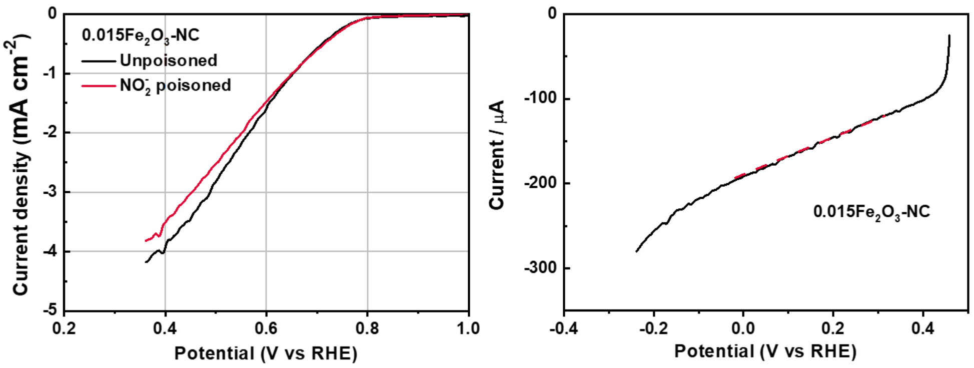

Supplementary Fig. 39 Determination of SD of 0.015Fe_2O_3-NC. Left column, LSV curves before and after nitrite adsorption in a 0.5 M acetate buffer at pH 5.2. Right column, CV curves during nitrite adsorption in the nitrite reductive stripping region. Catalyst loading is 0.242 mg cm^{-2}. The potential has been corrected by IR, and the resistance is automatically compensated by 80 %.

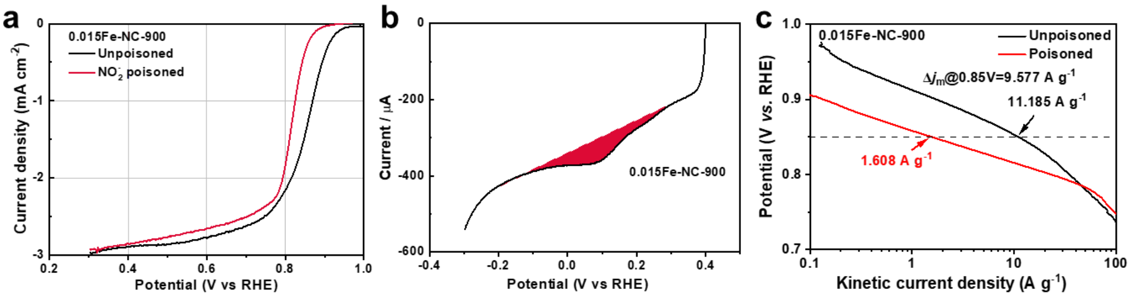

Supplementary Fig. 40 Determination of SD of 0.015Fe-NC-T. Taking 0.015Fe-NC-900 as an example, the ORR polarization curves before and after nitrite poisoning were first measured (Fig. 40a), and then the cv of nitrite poisoning was measured. The Q_{strip} was obtained by integrating the charge of the poisoning region (Fig. 40b), and the site density was further obtained. TOF is obtained by calculating the MA difference at 0.85 V before and after poisoning and combining the SD value (Fig. 40c).

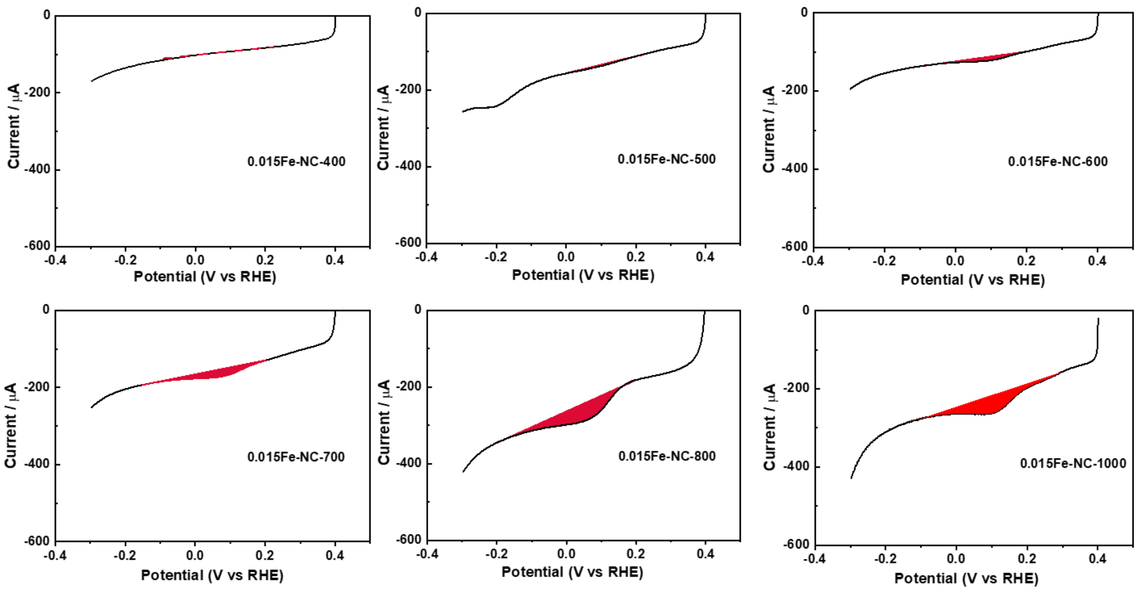

Supplementary Fig. 41 The Q_{strip} determined from poisoned CV of 0.015Fe-NC-T samples.

Supplementary Table 7. Summary of $Q_{stripping}$ and active Fe–N_4 sites.

Samples	$Q_{stripping}$ (C g^{-1})	Δj_m@0.85V (A g^{-1})	active Fe–N_4 (sites g^{-1})	TOF@0.85V (e $site^{-1}$ s^{-1})
0.015Fe-NC-400	0.28	/	$0.04\pm0.005\times10^{19}$	/
0.015Fe-NC-500	0.63	/	$0.08\pm0.01\times10^{19}$	/
0.015Fe-NC-600	2.92	0.282	$0.36\pm0.08\times10^{19}$	0.49±0.05
0.015Fe-NC-700	6.85	1.147	$0.86\pm0.10\times10^{19}$	0.83±0.06
0.015Fe-NC-800	13.61	3.778	$1.70\pm0.15\times10^{19}$	1.39±0.08
0.015Fe-NC-900	21.34	9.577	$2.66\pm0.20\times10^{19}$	2.25±0.1
0.015Fe-NC-1000	17.63	5.688	$2.20\pm0.13\times10^{19}$	1.68±0.06

Supplementary Table 8. ICP-MS data of 0.015Fe-NC-*T* samples.

Samples	Fe (wt%)	Fe ($\times10^{19}$ sites g^{-1})
0.015Fe-NC-500	1.52	16.39
0.015Fe-NC-700	1.51	16.28
0.015Fe-NC-800	1.50	16.18
0.015Fe-NC-900	1.52	16.39

Supplementary Table 9. The Fe atoms number of 0.015Fe-NC-*T*. (unit: $\times10^{19}$ sites g^{-1})

Samples	$N_{active\ Fe–N4}$[a]	$N_{inactive\ Fe–N4}$[b]	$N_{inactive\ Fe\ species}$[c]	$N_{total\ Fe}$[d]
0.015Fe-NC-500	0.08	3.94	12.37	16.39
0.015Fe-NC-700	0.85	4.58	10.85	16.28
0.015Fe-NC-800	1.70	5.40	9.08	16.18
0.015Fe-NC-900	2.66	4.56	9.17	16.39

[a]The data from **Supplementary Table 7** nitrite poisoning method; [b] $N_{\text{inactive Fe}-\text{N}_4} = \frac{x_{\text{ICP}}}{M_{\text{Fe}}} \times N_{\text{A}} \times x_{\text{LCF}} - N_{\text{aciive Fe}-\text{N}_4}$, x_{icp} is the Fe weight ratio by ICP-MS from **Supplementary Table 8**, M_{Fe} is the molar mass of Fe, N_A is the Avogadro constant, x_{LCF} is the FePc content by LCF fitting results from **Supplementary Table 5**; [c] $N_{\text{inactive Fe species}} = N_{\text{total Fe}} - N_{\text{aciive Fe}-\text{N}_4} - N_{\text{inaciive Fe}-\text{N}_4}$; [d]$N_{\text{total Fe}} = \frac{x_{\text{ICP}}}{M_{\text{Fe}}} \times N_{\text{A}}$.

Quantitative analysis process of different Fe species:

The number of active Fe-N_4 sites is derived from the nitrite poisoning experiment, because the active sites obtained by nitrite poisoning are electrochemically accessible, so we think that the active sites calculated by nitrite poisoning are the real active sites (contribute most to ORR current) involved in the ORR reaction. The number of total Fe-N_4 sites is obtained by LCF-fitting results. Because XANES results are bulk results, the content of different components of the sample can be distinguished by LCF fitting, so it can be considered that LCF-fitting can obtain the total Fe-N_4 sites. Combining the number of total Fe-N_4 sites and the number of active Fe-N_4 sites, we can calculate the number of inactive Fe-N_4 sites. The number of total Fe atoms is obtained from ICP-MS data (Supplementary Table 8). Further combining the number of total Fe atoms and the number of total Fe-N_4 sites, we can calculate the number of inactive Fe species.

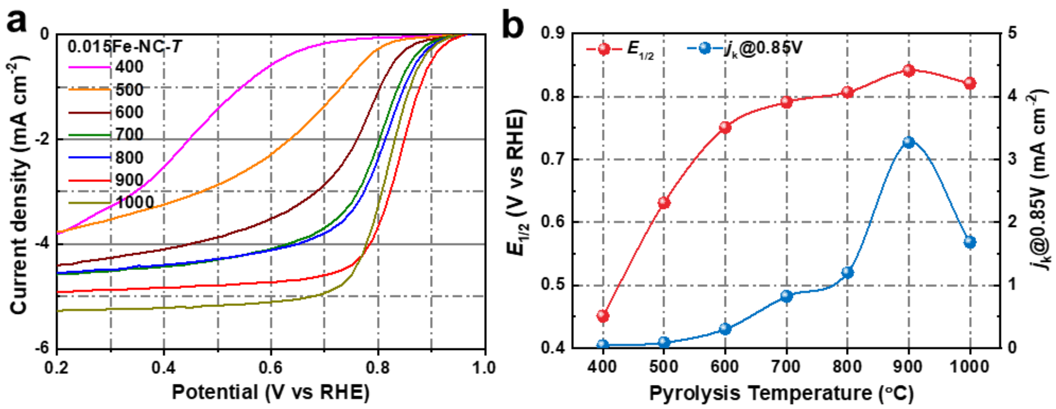

Supplementary Fig. 42 (a) ORR polarization curves under 0.1 M H_2SO_4. (b) the $E_{1/2}$ and j_k@0.85V as a function of the pyrolysis temperature. The potential has been corrected by IR, and the resistance is automatically compensated by 80 %.

As shown in Supplementary Fig. 42, when the pyrolysis temperature is 500 °C, we observed that the half-wave potential ($E_{1/2}$) of the material increased from 0.45 V to 0.63 V, indicating the formation of some active sites at 500 °C. The $E_{1/2}$ increased from 0.63 V to 0.75 V at 600 °C. As the pyrolysis temperature gradually rose to 900 °C, the $E_{1/2}$ of the material slowly increased from 0.75 V to 0.84 V. However, we observed that at 500 °C, the kinetic current density (j_k) of the material at 0.85 V and the TOF were low (**Figure 5c**, Supplementary Table 7, obtained by nitrite poisoning). We associated this with the electrochemical active sites density and found that the site density quantified by nitrite poisoning at 500 °C was only 0.08×10^{19} sites g^{-1}, while at 600 °C, the site density was 0.36×10^{19} sites g^{-1}, which is four times higher. Peter Strasser et al. found that nitrite adsorption could lead to a 70-80% reduction in ORR activity and they suggested that nitrite poisoning mainly affects the sites that contribute the most to the ORR current[1]. we found that when the density of active Fe-N_4 sites significantly increased (at a pyrolysis temperature of 600 °C), the TOF and mass activity at 0.85 V showed a significant increase (**Figure 5c-d**). Therefore, we think that at 500 °C, a small amount of the less active site (D2 site) is formed, quantified by Mössbauer spectroscopy as approximately 26.6% (Supplementary Table 10), which is similar to the results quantified by LCF (24%). At 600 °C, the formation of the highly active site (D1 site) begins.

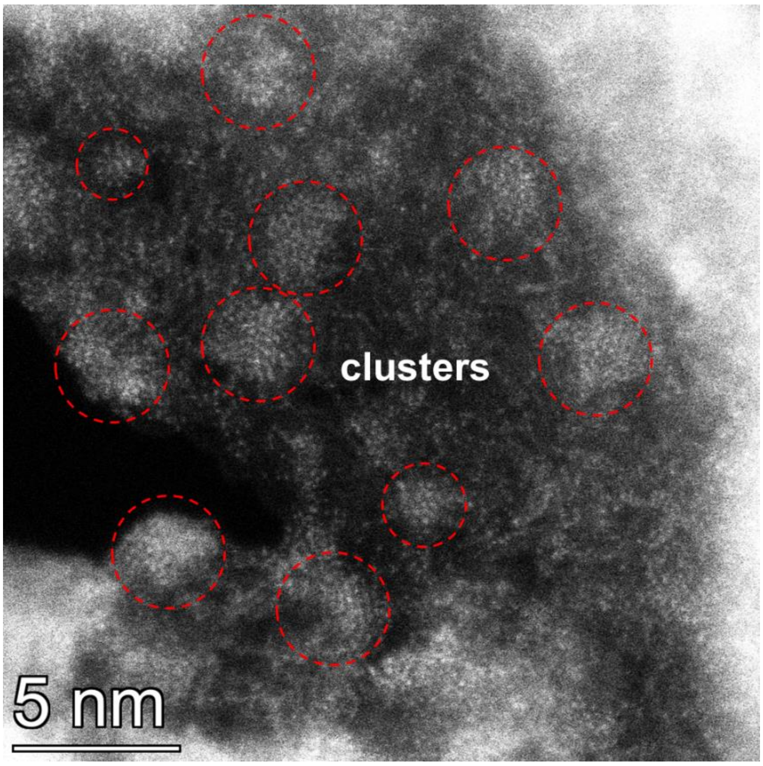

Supplementary Fig. 43 AC-HAADF-STEM image of 0.015Fe-NC-900.

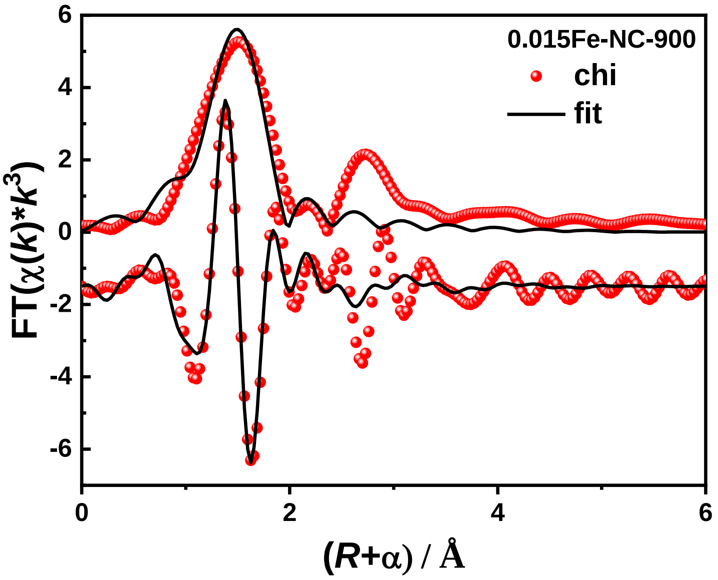

Supplementary Fig. 44 Fe K-edge EXAFS (points) and curvefit (line) for 0.015Fe-NC-900, shown in *R*-space (FT magnitude and imaginary component). The data is k^3-weighted and not phase-corrected. Surprisingly, no significant Fe-Fe scattering peak was seen in the FT-EXAFS of the 0.015Fe-NC-900 (**Fig. 44**). It is questionable to rely heavily on EXAFS spectroscopy technology to confirm that Fe/N/C materials are composed of atomic dispersed Fe–N_4 sites.

Recently, Simon *et. al.* reported that EXAFS characterization of heterogeneous single-atom catalysts may not be able to detect metal oxide species with a concentration of 40 at% or more, even if they are clustered.[2]

Supplementary Notes

Note 5. The ^{57}Fe Mössbauer spectroscopy of 0.015Fe-NC-T during pyrolysis process

Supplementary Fig. 45 clearly demonstrates that with decreasing nanoparticle size, the magnetic splitting spectrum of FeO_x transitions into a superparamagnetic spectrum and doublet. At room temperature, the 0.015Fe-NC-RT precursor consists of 90.2% FeOOH and 9.8% $FeCl_2·4H_2O$. This aligns with the analysis reported in our manuscript, suggesting that $FeCl_2·4H_2O$ undergoes oxidative hydrolysis at room temperature to yield $Fe(OH)_3$. At 400 °C, the FeO_x content in the product is 84.6%, with the remaining 15.4% attributed to the D2 sites, which correspond to the low-spin $Fe^{II}N_4$ sites, with a higher quadrupole splitting (QS) value[3]. Consequently, the oxidative hydrolysis occurring at room temperature is implicated in the high-temperature formation of FeO_x. Upon exceeding 600 °C, the FeO_x quantity diminishes to 73.4%. However, at temperatures above 700 °C, the sext signal is markedly reduced, leaving only the doublets associated with D1 and D2 peaks. D1 peak is presumed to represent a high-spin Fe^{III} species, potentially comprising high-spin $Fe^{III}N_4$ sites with axial oxygen ligand and superparamagnetic FeO_x nanoclusters[3]. At 900 °C, the spectrum retains the two doublets, which are ascribed to high-spin FeN_4 and low-spin FeN_4 sites, respectively. In conclusion, our observations confirm the formation of FeO_x at lower temperatures (< 600 °C) and their subsequent transition to FeN_x sites at elevated temperatures.

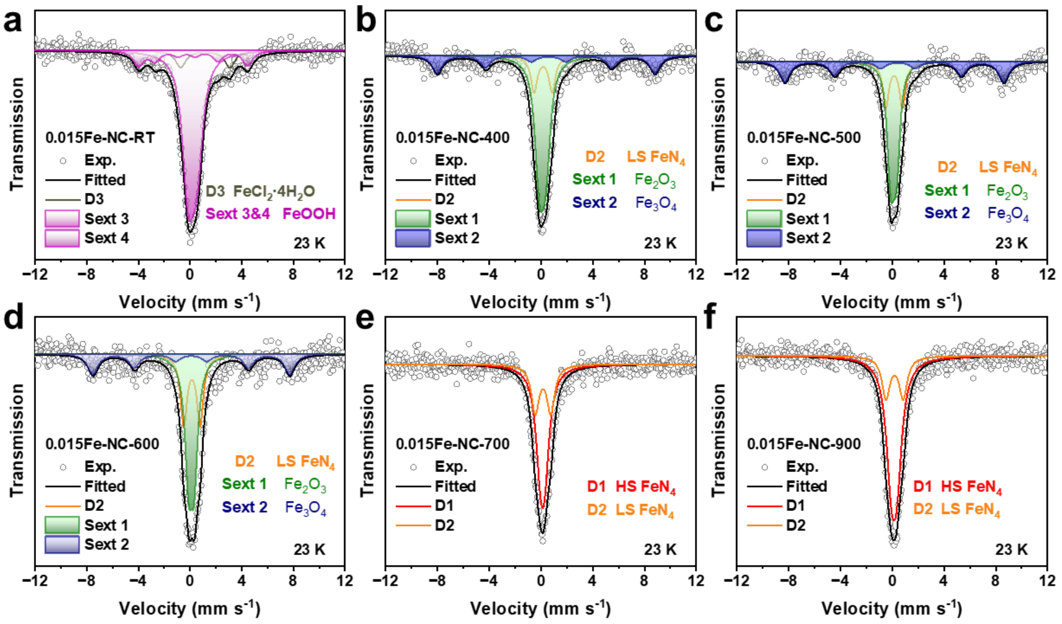

Supplementary Fig. 45 The ^{57}Fe Mössbauer spectra of 0.015Fe-NC-*T* at 23 K.

Supplementary Table 10. Average Mössbauer parameters of different samples and assignments for the iron species.

Samples	Comp.	RA %	IS mm s^{-1}	QS mm s^{-1}	LW mm s^{-1}	H Tesla	Assignment
0.015Fe-NC-RT	D3	9.8	1.1750	3.8090	0.9582	/	$FeCl_2{\cdot}4H_2O$
	Sext 3	71.2	0.1869	0.0898	0.9582	2.88	FeOOH
	Sext 4	19.0	0.0092	0.5023	0.9582	26.18	FeOOH
0.015Fe-NC-400	D2	15.4	0.1643	1.4009	0.6001	/	LS $Fe^{II}N_4$
	Sext 1	59.8	0.1445	0.0448	0.7460	2.13	Fe_2O_3
	Sext 2	24.8	0.5202	-0.2046	0.9582	52.21	Fe_3O_4
0.015Fe-NC-500	D2	19.5	0.1459	1.2759	0.5620	/	LS $Fe^{II}N_4$
	Sext 1	48.8	0.1274	0.0284	0.5512	1.93	Fe_2O_3
	Sext 2	31.7	0.3273	-0.2939	0.9582	52.53	Fe_3O_4
0.015Fe-NC-600	D2	26.6	0.1416	1.2593	0.6034	/	LS $Fe^{II}N_4$
	Sext 1	47.8	0.1405	0.0448	0.6760	2.08	Fe_2O_3
	Sext 2	25.6	0.1199	-0.0130	0.9582	47.34	Fe_3O_4
0.015Fe-NC-700	D1	67.9	0.1276	0.3223	0.8878	/	HS $Fe^{III}N_4$
	D2	32.1	0.1587	1.1987	0.7215	/	LS $Fe^{II}N_4$
0.015Fe-NC-900	D1	55.4	0.1383	0.2968	0.8959	/	HS $Fe^{III}N_4$
	D2	55.6	0.1704	1.1702	0.8925	/	LS $Fe^{II}N_4$

Supplementary Notes

Note 6. The changes in pore structure and carbon defects during thermal activation process

We investigated the evolution of the specific surface area and pore structure of 0.015Fe-NC-*T* materials during pyrolysis using nitrogen adsorption-desorption isotherms. **Supplementary Fig. 46** presents the statistical analysis of the specific surface area and pore size distribution derived from N_2 isothermal adsorption-desorption tests. Compared with the control sample NC, the specific surface area of 0.015Fe-NC-RT decreased from 811.44 $m^2\ g^{-1}$ to 487.95 $m^2\ g^{-1}$, as shown in **Supplementary Fig. 46a** and **Table 11**. At ambient temperature, there is a reduction in the volume of micropores in the precursor, while the mesoporous volume remains unchanged (**Supplementary Fig. 46b** and **c**). This suggests that $FeCl_2{\cdot}4H_2O$ may occupy the micropores, undergoing oxidative hydrolysis to yield $Fe(OH)_3$. Upon heating beyond 400 °C, dehydration of $Fe(OH)_3$ leads to the formation of FeO_x nanoparticles, which frees the micropores, thereby restoring the pore volume to levels comparable with the NC substrate. As the temperature further increases to 800 °C, the nanoparticles diminish in size, liberating additional micropores and mesopores and consequently enhancing the specific surface area. Above 900 °C, the majority of nanoparticles decompose into isolated atomic sites and clusters. Concurrently, the etching effect of mobile nanoparticles and carbon consumption through carbothermal reactions lead to an augmentation in the proportion of micropores and mesopores within carbon supports, with the specific surface area increasing to 949.60 $m^2\ g^{-1}$.

The enhancement of mesoporosity and microporosity typically results in an increased occurrence of defects on the carbon support. Raman spectroscopy serves as a reliable technique for examining the structural attributes of carbon-based materials. In this study, we employed Raman spectroscopy to investigate the evolution of carbon defects during the pyrolysis of 0.015Fe-NC-T materials. The experimental findings are depicted in **Supplementary Fig. 47**. Peak deconvolution of the Raman spectra was conducted, where the I peak signifies the sp^2 hybridized domains of graphene, the D peak represents carbon defects, the D'' peaks indicate distortions due to C5 rings or heteroatoms, and the G peak corresponds to sp^2 hybridized graphene. Typically, the ratio of the D peak area to the G peak area serves as an indicator of the degree of carbon defectiveness. A higher ratio suggests a greater degree of defectiveness.

The deconvoluted spectra and the aggregated data are presented in **Supplementary Fig. 48** and **Table 12**, respectively. It is observed that the degree of carbon defects increases progressively with rising pyrolysis temperatures, attributable to the formation of an increased number of micropores and mesopores, corroborating with the results from BET analysis.

In conclusion, with escalating pyrolysis temperatures, nanoparticles undergo a gradual transformation from formation to dissolution, concomitantly enhancing the specific surface area of 0.015Fe-NC-*T* material. Furthermore, the etching effect of mobile nanoparticles and carbon consumption through carbothermal reactions lead to an augmentation in the proportion of micropores and mesopores within carbon supports, thereby amplifying the degree of carbon defects.

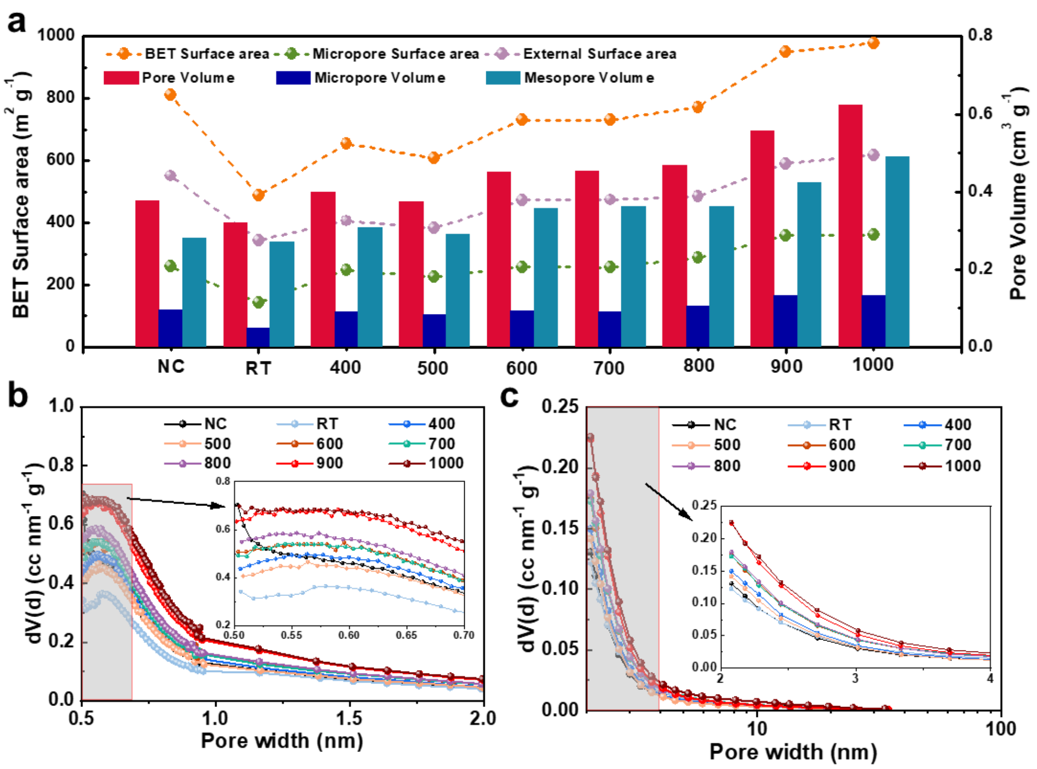

Supplementary Fig. 46 Structure evolution of 0.015Fe-NC-*T* carbon hosts during thermal activation at different temperatures. (a) A comprehensive comparison of BET surface areas and porosity; (b) Micropore size and (c) mesopore size distributions.

Supplementary Table 11. Porosity of 0.015Fe-NC-*T* samples.

Samples	S_{total} / $m^2\ g^{-1}$	S_{Micro} / $m^2\ g^{-1}$	$S_{External}$ / $m^2\ g^{-1}$	Pore Volume / $cm^3\ g^{-1}$	Micropore volume / $cm^3\ g^{-1}$
NC	811.44	260.13	551.31	0.378	0.097
0.015Fe-NC-RT	487.95	143.42	344.53	0.320	0.048
0.015Fe-NC-400	655.02	248.41	406.60	0.399	0.091
0.015Fe-NC-500	607.77	225.41	382.37	0.374	0.082
0.015Fe-NC-600	731.49	258.06	473.43	0.451	0.093
0.015Fe-NC-700	731.05	256.63	474.42	0.454	0.092
0.015Fe-NC-800	772.76	287.86	484.91	0.468	0.106
0.015Fe-NC-900	949.60	358.79	590.82	0.557	0.133
0.015Fe-NC-1000	979.17	360.90	618.27	0.623	0.133

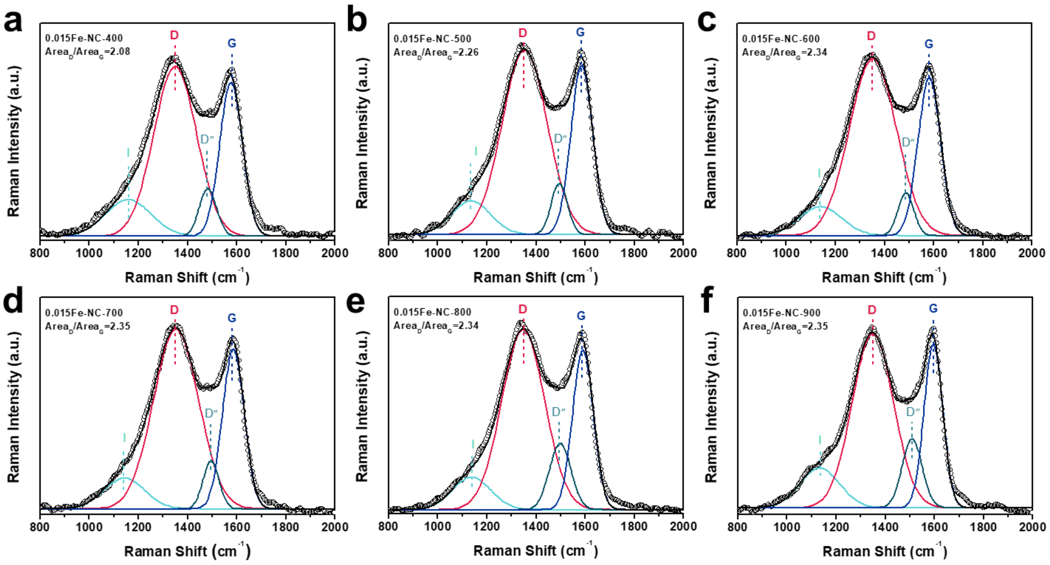

Supplementary Fig. 47 Raman spectra of 0.015Fe-NC-*T* samples obtained after thermal activation at different temperatures.

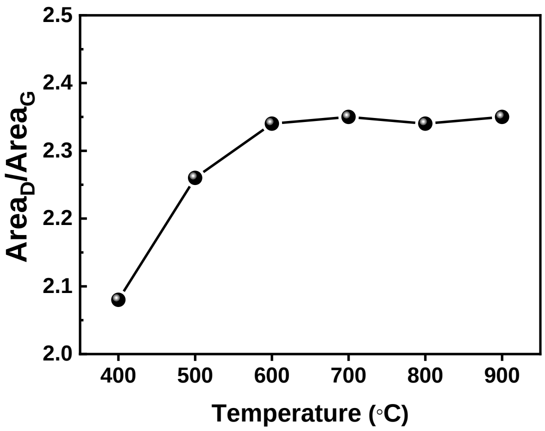

Supplementary Fig. 48 Area$_D$/Area$_G$ of 0.015Fe-NC-*T* samples obtained after thermal activation at different temperatures.

Supplementary Table 12. Raman fitting results of 0.015Fe-NC-*T* samples obtained after thermal activation at different temperatures.

Samples	I peak%	D peak%	D" peak%	G peak%	Area$_D$/Area$_G$
0.015Fe-NC-400	12.7%	54.9%	6.2%	26.3%	2.08
0.015Fe-NC-500	9.3%	58.8%	5.8%	26.0%	2.26
0.015Fe-NC-600	9.0%	60.3%	4.9%	25.7%	2.35
0.015Fe-NC-700	9.3%	59.7%	5.6%	25.4%	2.35
0.015Fe-NC-800	9.2%	56.3%	10.4%	24.1%	2.34
0.015Fe-NC-900	12.0%	54.4%	10.5%	23.1%	2.35

Fitting result was calculated by peak area; Area$_D$/Area$_G$ was calculated by the area at peak center; I peak: sp^2-C outside graphene; D" peak: distortion, C5 ring or heteroatoms.

Supplementary Notes

Note 7. The activity and durability of 0.015Fe-NC-900 cathode in H_2-air PEMFCs

Supplementary Fig. 49a-b show that the catalyst achieves a peak power density of 0.45 W cm^{-2} (at an absolute pressure of 1.5 bar) and a current density of 64.2 mA cm^{-2} at 0.8 V, positioning it at a mid-level among high-performance Fe-N-C catalysts (**Supplementary Table 13**). Besides, a rapid degradation was observed in the 0.015Fe-NC-900 cathode, with a 67% loss after 23 h (**Supplementary Fig. 49c**). This aligns with the instability commonly reported for Fe-N-C catalysts in existing literature.

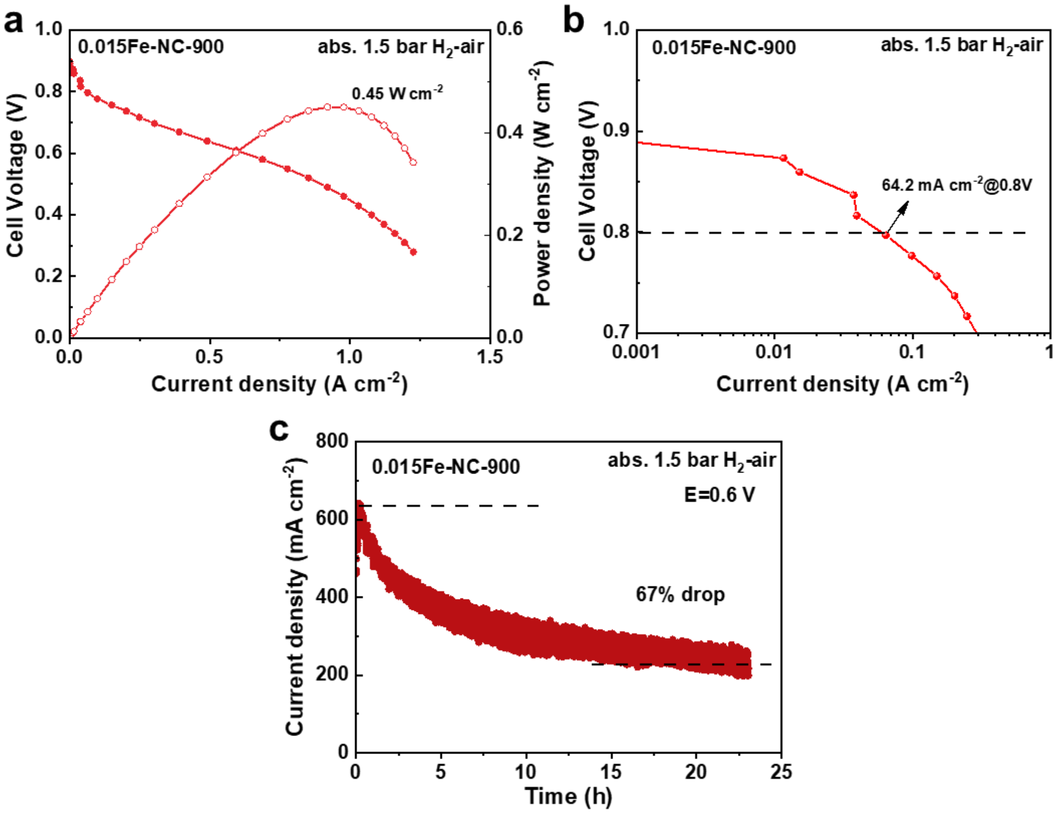

Supplementary Fig. 49 (a) H_2-air fuel cell *i*-*V* polarization and powder density plots of 0.015Fe-NC-900; (b) Current density at 0.8 V; (c) The fuel cell durability of 0.015Fe-NC-900 catalyst under H_2-air conditions at a constant potential of 0.6 V (1.5 bar absolute air pressure and flow rates of air 0.3 L min^{-1} and H_2 0.3 L min^{-1}. Anodes: Pt/C, 0.40 mg_{Pt} cm^{-2}, cathodes: Fe-N-C, 3.5 mg cm^{-2}, 100% RH, and 1.5 bar absolute H_2 pressure. Membrane: Nafion 211. Temperature: 80 °C. MEA area: 4.41 cm^2. The cell voltage and power density are not iR corrected.

Table 13. Comparison of H_2–air PEMFCs activity of 0.015Fe-NC-900 with other reported M-N-C catalysts.

**Catalyst**	$\boldsymbol{P_{max}}$ **/ W cm^{-2}**	***J*@0.8V / mA cm^{-2}**	**Abs. Pressure / bar**	**Ref.**
**0.015Fe-NC-900**	**0.45**	**64.2**	**1.5**	**This work**
Fe_g-NC/Phen	0.71	120.8	1.5	Energy Environ. Sci., 2022, 15, 3033-3040
Fe-N-C-FG	~0.72*	191.0	1.5	*Nat. Catal.*, 2023, 6, 1215-1227
Fe-AC	~0.60*	151	1.5	*Nat. Energy*, 2022, 7, 652-663
FeNC-CVD-750	~0.37	~40	1.5	*Nat. Mater.*, 2021, 20, 1385-1391
Fe-MOF catalyst	0.61	~50	1.0	*ACS Appl. Mater. Interfaces.* 2020, 12, 2216
SA-FeNx-ZIF8-PCM	0.60	~100*	1.9	*Nano Energy* 2021, 83, 105734
NPMC (CA#1)	0.57	~80*	1..74	*Sci. Adv.* 2018, 4, 7180
Mn−N−C−S	~0.500	~80*	2	*ACS Nano* 2021, 15, 6886
(Fe,Co)/N-C	0.505	54*	2	*J. Am. Chem. Soc.* 2017, 139, 17281
Fe/N/C(4mIm)-OAc	0.467	~40*	1	*Adv. Funct. Mater.* 2021, 31, 2009645
TPI@Z8(SiO_2)-650-C	0.42	105	1	*Nat. Catal.* 2019, 2, 259
(CM+PANI)-Fe-C	0.42	75	1	*Science* 2017, 357, 479
Fe-N-C-Phen-PANI	0.38	85*	1.88	*Adv. Mater.* 2017, 29, 1604456
FePhen@MOF-ArNH_3	0.38	50	2	*Nat. Commun.* 2015, 6, 7343
Co(mIm)-NC(1.0)	~0.32	~30*	1	*Nat. Catal.* 2020, 3, 1044
Fe-MOF-700/1000	0.302	~30*	1.5	*J. Mater. Chem. A* 2014, 2, 12270

*These values are not directly given in the papers, thus obtained by digging the polarization curves or calculated by the data provided.

Supplementary References

1. Primbs, M et al. Establishing reactivity descriptors for platinum group metal (PGM)-free Fe–N–C catalysts for PEM fuel cells. *Energy Environ Sci*, **13**, 2480-2500 (2020).

2. Finzel, J et al. Limits of Detection for EXAFS Characterization of Heterogeneous Single-Atom Catalysts. *ACS Catal*, **13**, 6462-6473 (2023).

3. Li, J et al. Identification of durable and non-durable FeN_x sites in Fe–N–C materials for proton exchange membrane fuel cells. *Nat Catal*, **4**, 10-19 (2021).
