# Supplementary material for: An in situ exploration of how Fe/N/C oxygen reduction catalysts evolve during synthesis under pyrolytic conditions
Source: Nat Commun. 2024 Jul 24;15:6229. doi: 10.1038/s41467-024-50629-x (PMC11266712; doi:10.1038/s41467-024-50629-x)
Supplement: Supplementary file 3 — Description of Additional Supplementary Files [file 41467_2024_50629_MOESM3_ESM.pdf]

Description of Additional Supplementary Files

File Name: Supplementary Movie 1
Description: Heating process from 0 s to 2419 s

File Name: Supplementary Movie 2
Description: Machine learning during heating from 0 s to 900 s

File Name: Supplementary Movie 3
Description: Machine learning during heating from 1407 s to 2419 s

File Name: Supplementary Movie 4
Description: Molecular Dynamics Simulation

File Name: Supplementary Data 1
Description: atomic coordinates
