# Supplementary material for: An in situ exploration of how Fe/N/C oxygen reduction catalysts evolve during synthesis under pyrolytic conditions
Source: Nat Commun. 2024 Jul 24;15:6229. doi: 10.1038/s41467-024-50629-x (PMC11266712; doi:10.1038/s41467-024-50629-x)
Supplement: Supplementary file 8 — Supplementary Data 1 [file 41467_2024_50629_MOESM8_ESM.docx]

Fe64O64

Fe O

1.00000000000000

20.0000000000000000 0.0000000000000000 0.0000000000000000

0.0000000000000000 20.0000000000000000 0.0000000000000000

0.0000000000000000 0.0000000000000000 20.0000000000000000

Fe O

64 64

Direct

0.4040784711732232 0.2889745884823576 0.2788327203517095

0.3991099253281922 0.2387938393052892 0.3841882724612163

0.4992985049322653 0.3711091378439965 0.2998261112945008

0.2953176041673589 0.3242514575788812 0.3593149037602795

0.3480838124832654 0.4143739219699400 0.2743466386507081

0.4036623885473610 0.3742259053235890 0.4034691522257995

0.4528568485137510 0.5079846163404592 0.2874768235686870

0.2835259800945744 0.4581566621736671 0.3648434847704491

0.5405530838147571 0.2594961484639651 0.3032820951630458

0.6658493496503396 0.2378177039264759 0.4698800114875041

0.6628420877542751 0.2797551926758177 0.3515330046670588

0.5426599702150433 0.2988955006460522 0.4221325108583009

0.6083501682694302 0.3655144946148039 0.2405882699830528

0.6679108581382703 0.3974963056438662 0.4548793644927486

0.6848852349186146 0.4518688203618523 0.3482574072440885

0.5284939452626587 0.4479671488382422 0.4096792406556336

0.3257594350002726 0.5548808759134819 0.2654127983247009

0.3850207745512245 0.5418928344374065 0.3998424914325638

0.4175183619622098 0.6264792159698395 0.2981232167862486

0.2994965922636975 0.6428862367209497 0.3712765060223059

0.3199089845135598 0.6964295215425080 0.2671475896123198

0.4111127271716860 0.6757128245593541 0.4106653981880690

0.4489105393517323 0.7463403227598597 0.2801560518561094

0.3380141747722746 0.7733755551915865 0.3761283024316266

0.5986067084875617 0.5028895732414110 0.2630930813739603

0.6597915692774868 0.5375956439018270 0.4561737150550295

0.6650477079299770 0.6222213177095739 0.3350333695349665

0.5286722709247151 0.6006074117708589 0.3965082519193375

0.5610633627519029 0.6642800744842818 0.2888317358753233

0.6568374242578499 0.7004150400025158 0.4639665072327295

0.6288697529512276 0.7503210030100844 0.3427076689206254

0.5295577419380290 0.7388505570280410 0.4029780260269972

0.2880242580825355 0.2664553838024658 0.4836328772611997

0.3964711122352100 0.2384679671572533 0.6547317111912447

0.3937745511811776 0.2986522939053196 0.5380832615238410

0.2872393458059915 0.2983745334352999 0.6193537508632080

0.2671443298545836 0.3800111536449360 0.4869984239842710

0.4024393422147585 0.3752008005947691 0.6618878973129888

0.3850955269324963 0.4640476250370641 0.5259316782520622

0.2879960652766961 0.4631798568277172 0.6159966692454701

0.5304510836642454 0.2363921442383954 0.5562343327149170

0.6578755771530954 0.2353361748287676 0.7212123519633027

0.6725231699786192 0.3062225584974388 0.6014496123248806

0.5459894156331767 0.2956154947833177 0.7061968468022285

0.5323626069201099 0.3858210736528681 0.5599720552977285

0.6721182514087074 0.3826538798475946 0.7446830006271609

0.6667053670323693 0.4610110933091240 0.6061520717542380

0.5353197533214991 0.4609112311141610 0.7056954302179188

0.2754632228517375 0.5267028228748367 0.4918868477452424

0.4044073031493474 0.5334770366154473 0.6663987255074487

0.3876621752086641 0.6197838828663819 0.5295169344411715

0.2904808373289027 0.6305561736817743 0.6315618318475966

0.2878635146601256 0.7021397646890380 0.5078789544568451

0.4068286577652324 0.6968181170174184 0.6763552155843304

0.3933293913465404 0.7563642224294077 0.5228189472204882

0.2953615902266090 0.7416353846502305 0.6347201800273108

0.5300275668612381 0.5423096671806730 0.5597402276801855

0.6683962652116955 0.5401085763119103 0.7444877003547282

0.6727394729088312 0.6188982272029032 0.6023120664119589

0.5380945756519134 0.6218338745570752 0.7092750336310414

0.5339038465421031 0.6977240969827884 0.5671911836811803

0.6645854933206454 0.6954350223527931 0.7318431750399765

0.6579674079896308 0.7653009366537470 0.6104324313182632

0.5479053315113632 0.7667812211249921 0.6988179759927539

0.4585569356493450 0.2179224837346081 0.3080728131876239

0.3060116136628325 0.2330904996742517 0.4005984253277090

0.3216914034297454 0.3281588778164126 0.2638964232018040

0.4446033869014082 0.2962972289317400 0.4490022434639697

0.5372864110160195 0.4284149213284356 0.2310097234805563

0.2347737627075577 0.3888255778281475 0.3948385097468157

0.3798251303608542 0.4884398962406675 0.2310357276260948

0.4322872242973524 0.4611803725379691 0.4404920013880930

0.6934096894241043 0.2072707788984023 0.3915752925602917

0.5751551745081227 0.2233872225060140 0.4769531639220651

0.6249937520948252 0.2764780895430075 0.2650937075533694

0.6970073051644452 0.3117576397926560 0.5127825980868945

0.6995826525528256 0.3639181793156198 0.3709597984812222

0.5663304646536784 0.3848537426241579 0.4717874427049842

0.6703809869114813 0.4377397433069845 0.2574328710754255

0.6875639738707104 0.4644555143089474 0.5174742019760198

0.5328937493646790 0.5158078145421339 0.3389617184524335

0.2813671679385545 0.5466416758182507 0.3976643990162164

0.2653268611021023 0.6235148607936312 0.2695674856171338

0.4455171165897770 0.5993653872458015 0.4534519707218759

0.4816757486348814 0.6650447329718666 0.2395562449375871

0.2691104383610615 0.7238780108512919 0.4169231277659954

0.3603221734281761 0.7804535932635804 0.2833359367319072

0.4174749376987588 0.7749894064374463 0.4321319806013655

0.7012995416697695 0.5399732355366265 0.3686021224451667

0.5642706413203787 0.5328128882839677 0.4707037991494226

0.6162256612242136 0.5975233429419307 0.2573180332652767

0.6839030418445838 0.6152853465154973 0.5089199794038464

0.6996405280790943 0.7002925374559680 0.3741541918827244

0.5580695903751798 0.6929109130840047 0.4779796244434452

0.5417501489847897 0.7676577512476591 0.3094625167320593

0.6817515093162403 0.7655278867005338 0.5263641532213518

0.4423916094854640 0.2180995376848509 0.5744645154435457

0.3083442266822084 0.2318196702558703 0.6753481890494811

0.2330308585612491 0.3056817997593295 0.5450530892335205

0.4529226286414766 0.3010153036272899 0.7003315677646922

0.4389647261525929 0.3855735473750370 0.5681031139183458

0.3043940894003599 0.3818931661178385 0.6605122853065936

0.2325113808125732 0.4575035520775816 0.5409380609082234

0.4423490501062141 0.4569779700223484 0.7022023611308410

0.6969248042051484 0.2306432137716768 0.6419977313655318

0.5716210970998586 0.2121491790649193 0.7213776140812727

0.5699526766572894 0.3109884967716983 0.6036383127500176

0.6897637560664137 0.2993241322182199 0.7728089467494900

0.6944894204546144 0.3856520110262474 0.6497635678736129

0.5724527463097298 0.3808784403230679 0.7386671599333355

0.5656922414273158 0.4632502349995132 0.6084886858484875

0.6953640708643547 0.4620856836914643 0.7770487859558145

0.4343147997037929 0.5361391932272788 0.5754633275128266

0.3099783335024686 0.5398865787343831 0.6623202328121356

0.2846181627596980 0.6096212544395129 0.5401056136929571

0.4453700476530683 0.6089753058756687 0.7004329487705335

0.4415757571220423 0.6927604573348012 0.5784999127660750

0.3136396534698107 0.6845599759113105 0.7045652551327054

0.3069240427631093 0.7859468813446939 0.5563883369594108

0.4606415305449232 0.7668442609797851 0.7054192946907021

0.6904784788993032 0.5414218021049190 0.6482267744016055

0.5743040778973669 0.5391181585697505 0.7395723299431632

0.5675434753636162 0.6185049188535973 0.6096257430711830

0.6947690057008499 0.6248967958780526 0.7730020909511836

0.7108382641125095 0.6938835886229087 0.6482272522255710

0.5727011175752652 0.6954028273171292 0.7591895717208490

0.5627350531419790 0.7796830732023017 0.6044462646261444

0.6415780953712102 0.7832476213484011 0.7024031400540063

Fe48O48

Fe O

1.00000000000000

20.0000000000000000 0.0000000000000000 0.0000000000000000

0.0000000000000000 20.0000000000000000 0.0000000000000000

0.0000000000000000 0.0000000000000000 20.0000000000000000

Fe O

48 48

Direct

0.3095169767511297 0.3538689178671203 0.3381720671730234

0.4206157705170877 0.3089689867976891 0.3835624809893444

0.4217283968178456 0.3713481416025589 0.2731233427280624

0.2640394119883625 0.4418266784353153 0.3786775326517014

0.2867721532930562 0.3359593765769553 0.4638854139609923

0.3996411829185826 0.3096585833807545 0.6413589587573094

0.4042469283664271 0.3537399482484334 0.5288350717761049

0.2849338205768420 0.3657477758078110 0.6047626412295198

0.3216516501139849 0.4680672865120782 0.2688537974119360

0.4044628719421393 0.4584601905930648 0.3902771547862059

0.4298312364649574 0.5178171774596162 0.2876105911837695

0.2896922758498262 0.5650302555704064 0.3848249782492677

0.2466243636344191 0.4515980248657647 0.5006681672789097

0.3932251010433537 0.4487844896605303 0.6488005693344351

0.3802402784512774 0.5360281065407112 0.5173559036247782

0.2746715284135616 0.5427181141458159 0.6225240603696759

0.3487844155805607 0.6251620292942980 0.2817311049282998

0.4246805863849478 0.6109803395656357 0.4028733599280110

0.4522617000227178 0.6787888842628540 0.2946539178940681

0.3222809293661471 0.6871636558914878 0.3947609586655319

0.2504281795323542 0.6198628138492414 0.5170069436866138

0.4037056963417734 0.6088929387870093 0.6645643015412757

0.3775443547071337 0.6803340492161748 0.5067166047034655

0.2917021146015362 0.6559479432525261 0.6285689260076506

0.5444824782988611 0.3331441527839092 0.2981503583366405

0.6724830383851261 0.3128826948725652 0.4565891461902701

0.6722617727177060 0.3695580161583072 0.3379663150110718

0.5548603455174087 0.3720494794667847 0.4153751612059066

0.5586729678639903 0.2988229202885289 0.5650949671267879

0.6522547606545415 0.3201704037645902 0.6990329915589217

0.6554604785060414 0.3854263057432574 0.5956905351719222

0.5351786054183565 0.3841078916765790 0.6915044803575893

0.5680087512527098 0.4436775353302849 0.2771875834686741

0.6791971491555769 0.4543236480852347 0.4555357860341535

0.6824257167393931 0.5348359840966865 0.3277061561184819

0.5591971073898917 0.5459746530647651 0.3997728501045038

0.5188738162827475 0.4702288177386370 0.5337386371229377

0.6675045699086656 0.4591306267366915 0.7295091161334534

0.6612797939794470 0.5381935552287969 0.5900313882369231

0.5362380517614479 0.5391305903832306 0.6895940789516705

0.5731591175029673 0.5887700303479987 0.2698956693241158

0.6771242724123914 0.6208995172106028 0.4584346906977818

0.6492145348015032 0.6552287539263545 0.3388745954263153

0.5296901391752195 0.6853720538132247 0.4016707782902208

0.5176584225446804 0.6267852835939607 0.5444727780813156

0.6563667712756878 0.6141166319442832 0.7216152447748635

0.6554958959538245 0.6901856633583707 0.5976336334454861

0.5428831513989402 0.6829355323640729 0.6868657866248191

0.4644591060107983 0.2912623768574604 0.3009479180442298

0.3658969448333793 0.2920199439325497 0.4614778129034211

0.3311682702799991 0.3771897655654341 0.2500811724802133

0.4605340237712999 0.3857056628406224 0.4266104287143289

0.4646836515038975 0.2910245532353402 0.5797684932081811

0.3136524664871099 0.2957604117290021 0.6561928620570807

0.2269951981368153 0.3651430254291054 0.5273141187145102

0.4392122614514186 0.3742834572395606 0.6929749516427509

0.5171603094095655 0.5106615856531975 0.3172572846821160

0.3179705758335587 0.4965218061845756 0.4466532222809845

0.3544308987876676 0.5482328258135132 0.2349503749005132

0.4535795812856796 0.5293540972707088 0.4448470092904671

0.4234071108592096 0.4491807237168685 0.5532643992565688

0.2971200901729288 0.4517685206111776 0.6447210496099147

0.2230897671039041 0.5329120610657169 0.5457282465152618

0.4428061708505921 0.5218904440566274 0.6863944155189123

0.5100459967963469 0.6443448731898962 0.2400212162731890

0.2465106833856359 0.6382443998850590 0.4205714864819558

0.3677663883860192 0.7125969120349779 0.3166943818128914

0.4599986021664667 0.6863259353561507 0.4631682072837727

0.4246136350096532 0.6058088682371756 0.5669255567712761

0.3102964739535827 0.5926869377820476 0.6929731559816640

0.2955971509519860 0.7010966149190240 0.5481969104948214

0.4550301332184670 0.6826904220101039 0.6896943551343402

0.7077853982894361 0.2978526823398941 0.3767187970816511

0.5818582966969996 0.2971710275817752 0.4731099390704573

0.6193037695021921 0.3689160662663734 0.2590133545162306

0.7028314786310911 0.3806434099776294 0.5109093922033732

0.6417237335273580 0.2871859063928877 0.6131694483643091

0.5716694235124888 0.3083483854680681 0.7318001623351007

0.5569707207952909 0.3992707564892638 0.5933376526421243

0.6950912922031819 0.3746539361045520 0.7518985439684489

0.7114526974220350 0.4546098510836163 0.3638605052597116

0.5790246145573013 0.4612173068394977 0.4558916998983243

0.6313657593791292 0.5132692400784754 0.2544405172773126

0.6973887465632124 0.5364867540918935 0.5022355314564065

0.6894350477988981 0.4631189161634711 0.6377517233593634

0.5739736491541305 0.4622040139557991 0.7299330079975904

0.6417237335273580 0.2871859063928877 0.6131694483643091

0.5716694235124888 0.3083483854680681 0.7318001623351007

0.5569707207952909 0.3992707564892638 0.5933376526421243

0.6950912922031819 0.3746539361045520 0.7518985439684489

0.7114526974220350 0.4546098510836163 0.3638605052597116

0.5790246145573013 0.4612173068394977 0.4558916998983243

0.6313657593791292 0.5132692400784754 0.2544405172773126

0.6973887465632124 0.5364867540918935 0.5022355314564065

0.6894350477988981 0.4631189161634711 0.6377517233593634

0.5739736491541305 0.4622040139557991 0.7299330079975904

0.5610943608015072 0.5429677801518277 0.5908742784065398

0.6885677765359669 0.5430427102241980 0.7587245603192599

0.7221911241541571 0.6078550032962933 0.3678765746478438

0.5764878664529468 0.6163492952308693 0.4657781908410821

0.5912168412655171 0.7220162316632155 0.3466281514664178

0.6885407027519946 0.6891174637195657 0.5157499493058708

0.6970983675925311 0.6114616484860004 0.6348625322130971

0.5636510147098531 0.6122407785043760 0.7480639780164310

0.5595339901473168 0.6954430368851200 0.5918831782232346

0.6342719038702719 0.7029910409695084 0.6946842904333187

Fe32O32

Fe O

1.00000000000000

18.0000000000000000 0.0000000000000000 0.0000000000000000

0.0000000000000000 18.0000000000000000 0.0000000000000000

0.0000000000000000 0.0000000000000000 18.0000000000000000

Fe O

32 32

Direct

0.3748202368496846 0.5015504450288663 0.3457567527025189

0.4729193014878926 0.4270246410933295 0.4370300365423148

0.5002080582832678 0.5158769421216832 0.3297329619304378

0.3072206711028767 0.5050200277661683 0.4750822298664053

0.3974460183219488 0.4170822037686102 0.5978308303141705

0.4499337953137068 0.4250237489700144 0.7334309887781162

0.4510909475694866 0.5663670144132145 0.6206922566476699

0.3225053327375995 0.5391494137577014 0.6325733408286944

0.4056657516406342 0.5901571398427299 0.2533771685415281

0.4391443817054881 0.5984466208526436 0.4660780633283948

0.4623449021399292 0.6606018990136219 0.3382169553553891

0.3211302078427161 0.6259223372876079 0.3737751295922560

0.3213933450061451 0.6440282408389065 0.5315754678799762

0.4011686861319053 0.5671202349063313 0.7722830925077463

0.4588446823634746 0.7096743891201382 0.5436722617486391

0.3755043209073582 0.6826775277202176 0.6817715418337630

0.6373991911601792 0.4350602428326901 0.3551870334352197

0.7018795310745187 0.4278854766288134 0.4862987883488809

0.7675638303030453 0.5142216281972681 0.3654664308537542

0.6207731147086315 0.5388817435493783 0.4537477986690718

0.5505554624104911 0.4439248269455356 0.6055172057416692

0.7118621820572576 0.4376111906458824 0.8145551364251106

0.7218815100054704 0.4567749863500673 0.6409294085761400

0.5590803681206526 0.4896103667347591 0.7957100224226338

0.6454189476718999 0.6081388599560263 0.3239252040805091

0.7754182334327719 0.5805978446468594 0.5135836020244730

0.7833685554258082 0.6424575377616510 0.3888493840470421

0.6235152260507136 0.6962799291726897 0.4348648755477764

0.5966181497292917 0.6526956366073745 0.5877118259859625

0.7070274297515853 0.5906820121690711 0.7801236999606975

0.7474472204000050 0.6544896393599959 0.6602238336044011

0.5226083392467008 0.6466253572882544 0.7418268351216283

0.5378033557304315 0.4190031447988703 0.3503059827781242

0.4805454692483746 0.3979542021344695 0.5358587838580431

0.3644589825326706 0.4345415002114726 0.4223261158232246

0.5163800825212298 0.5326560983629707 0.4552631798677382

0.5483858141559422 0.4216943298068423 0.7079501313539246

0.3860952610043263 0.3698835581389476 0.6840473822310796

0.3033976150979090 0.4600263709217526 0.5696589213773067

0.4499410907883422 0.4872287572744223 0.8162459042013811

0.5613519851682602 0.6670630187464914 0.3427240069534562

0.2635218735004654 0.6010822546664196 0.4545734532801646

0.3664838335064487 0.6773172686488135 0.2982903212641733

0.5498687947152825 0.7232925835683202 0.5008465762175467

0.4962863426920712 0.6850702755479010 0.6453390283000342

0.3118039235061524 0.5932779968305927 0.7169692635804303

0.3604815673021456 0.7248445692020309 0.5819730641149231

0.4260809009046336 0.6711461181178733 0.7804505076951697

0.7327852153407438 0.4106417393241913 0.3914621173184878

0.6458702079288451 0.4118470317975364 0.5698536423211731

0.6847224741719936 0.5168256437673200 0.2967990568532082

0.7713025132255378 0.4756601001524996 0.5443290990106069

0.7354642474551518 0.4059074265223125 0.7243440904918902

0.6206163894984683 0.4346050842941637 0.8502663460470761

0.5541250131865110 0.5491554190850213 0.6058935004090622

0.7513239502338406 0.5236988137793239 0.8384579828868037

0.8406034407589869 0.5657436604193803 0.4208537569502376

0.6636816962075690 0.6154387960397604 0.5037536256386560

0.7018311219078556 0.6955818193516010 0.3624844340435735

0.8059630727192481 0.6461419073089479 0.5807894666286155

0.7145862480691720 0.5529095922747344 0.6760662598174023

0.5965427312607557 0.5905202802628635 0.7892673319117488

0.6632708065247009 0.7029515619703873 0.6392284586129401

0.7629993225335118 0.6702751298395032 0.7576457142579359

Fe13O13

Fe O

1.00000000000000

18.0000000000000000 0.0000000000000000 0.0000000000000000

0.0000000000000000 18.0000000000000000 0.0000000000000000

0.0000000000000000 0.0000000000000000 18.0000000000000000

Fe O

13 13

Direct

0.4540722224226812 0.3696643957270709 0.4969609981428320

0.4650425745182076 0.4197142397821167 0.3677142178572694

0.3541847329590908 0.4691400046792253 0.4557251335445045

0.3949028769243768 0.4867597323056287 0.5921224611491027

0.4291421404574791 0.5620528576951287 0.3660427704415739

0.4971129930381437 0.5019842993120491 0.4885246355180897

0.5905620447093085 0.5348072286443397 0.3835968421544109

0.4104728652103731 0.6078312789483036 0.5118496828532884

0.5309996016356990 0.5996552559921255 0.5935288210687872

0.5854019740244567 0.4067536572777399 0.4301709442751616

0.6410960105274626 0.5491369935596921 0.5138593393637834

0.5408367168744945 0.4695821778536029 0.6230823883501415

0.5171758671817486 0.6387842181454744 0.4332823405113402

0.5119797648978235 0.3335870718514826 0.4138997091174801

0.3608434978558464 0.3959326668709977 0.5293422412324773

0.3750054620010417 0.4728848268743775 0.3534014107477645

0.5550859246687097 0.4057517465305480 0.5330726732954574

0.4898430867150836 0.6718508329404287 0.5337193406122296

0.5677101082050910 0.4450280640294366 0.3349100825892819

0.3321742109477555 0.5445856612886928 0.5289725973963684

0.5238843882124442 0.5989928567449354 0.3348751481946621

0.5309996016356990 0.5996552559921255 0.5935288210687872

0.5854019740244567 0.4067536572777399 0.4301709442751616

0.6410960105274626 0.5491369935596921 0.5138593393637834

0.5408367168744945 0.4695821778536029 0.6230823883501415

0.5171758671817486 0.6387842181454744 0.4332823405113402

0.5119797648978235 0.3335870718514826 0.4138997091174801

0.3608434978558464 0.3959326668709977 0.5293422412324773

0.3750054620010417 0.4728848268743775 0.3534014107477645

0.5550859246687097 0.4057517465305480 0.5330726732954574

0.4898430867150836 0.6718508329404287 0.5337193406122296

0.5677101082050910 0.4450280640294366 0.3349100825892819

0.3321742109477555 0.5445856612886928 0.5289725973963684

0.5238843882124442 0.5989928567449354 0.3348751481946621

0.6171327710294392 0.5451933080922515 0.6189762375941328

0.4512224501546243 0.4484100260658863 0.6646479747054670

0.6206400295923515 0.6212156040102883 0.4374921103407345

0.6628706166101708 0.4757383734480598 0.4374675135592412

0.4116035815273946 0.6529976339531203 0.4187088392411127

Fe4O4

Fe O

1.00000000000000

18.0000000000000000 0.0000000000000000 0.0000000000000000

0.0000000000000000 18.0000000000000000 0.0000000000000000

0.0000000000000000 0.0000000000000000 18.0000000000000000

Fe O

4 4

Direct

0.5252841189323448 0.4517876905693176 0.5316852249147810

0.5288633788812199 0.5186857399601589 0.4209273873257802

0.4018537514911457 0.4716819311306459 0.4640570349225161

0.4794678446062608 0.5731171073468695 0.5278948539792331

0.4247933443675305 0.4845095694927578 0.5678916189039915

0.5838410784333627 0.5428943704880047 0.5129099199554967

0.4293778707380896 0.5688385181833728 0.4283160485660270

0.4877032622677173 0.4155698018355753 0.4329957086987601

Fe113

Fe

1.00000000000000

23.0000000000000000 0.0000000000000000 0.0000000000000000

0.0000000000000000 23.0000000000000000 0.0000000000000000

0.0000000000000000 0.0000000000000000 23.0000000000000000

Fe

113

Direct

0.3180031270876397 0.3193462790361435 0.4441466212932756

0.3179843374711361 0.3182072260603893 0.5644145967870681

0.3183115527695123 0.4374136840169132 0.3267150501104603

0.3151641941880267 0.4384706118418664 0.4458469820947053

0.2578493485085093 0.3817460675578092 0.5061304591869594

0.3162305157608745 0.4379993298188514 0.5686940510361570

0.3257625395228816 0.4408690946838637 0.6900280439671030

0.3183804081093061 0.5607875352496009 0.3264719469653333

0.2582816535142724 0.4990626032403216 0.3865368050609173

0.3152529066464317 0.5604807398977646 0.4443492073941552

0.2554176618163786 0.5012573072303814 0.5109573494093326

0.3167534415687088 0.5620708810999420 0.5668877398611377

0.2600404880157526 0.5011726778972163 0.6259715948541741

0.3204853567750258 0.5644782582892481 0.6825061801860368

0.3221042814157754 0.6789993057864140 0.4453545797012819

0.2574412036271795 0.6164667792610841 0.5042261686039410

0.3244061144916761 0.6768916726327577 0.5701919301930198

0.4336223157785843 0.3230899929791431 0.3258783934815492

0.4386952472439056 0.3110959276081878 0.4426626388929197

0.3776395507675221 0.2574286527478916 0.5035268832896235

0.4379883262109384 0.3121529734088069 0.5671808967856183

0.4399980505678033 0.3160512112194993 0.6865976819315115

0.4366813809759664 0.4376855363527856 0.3211435228459375

0.3793052249463312 0.3799597641108383 0.3877329239239818

0.4390963686774145 0.4378491002543408 0.4454168075601237

0.3776369714302518 0.3762874360150469 0.5060224489678682

0.4381271750446005 0.4368613065265224 0.5671166565092377

0.3782974739779863 0.3768470765967568 0.6270894927571062

0.4402906463560154 0.4338078202724785 0.6888776350179596

0.3769021926211570 0.4982974003212302 0.2642084824519713

0.4370215076837078 0.5592973955239435 0.3196138659879641

0.3774098703739863 0.4987339820173735 0.3843520890295805

0.4391292949840997 0.5594792651424264 0.4451924807852921

0.3779947549794532 0.4987341074820175 0.5063030785403194

0.4385751376452106 0.5594831411780541 0.5656095125316899

0.3793677423735039 0.5001043892948973 0.6273780228115351

0.4380993551474310 0.5617755049366684 0.6875751061461061

0.3846427907601832 0.4969738116947778 0.7454971108383424

0.4395828485834826 0.6770454823325613 0.3256641738466367

0.3808556739614036 0.6181013814311411 0.3861044770777511

0.4391418324973664 0.6844669104713418 0.4422411108101911

0.3788321011409477 0.6215085856043354 0.5042739346844973

0.4402991672118433 0.6834597286336949 0.5646652026638298

0.3818852678528608 0.6200210958847586 0.6255138342970870

0.4414067456944389 0.6783080933277659 0.6852767740509271

0.3839079543471732 0.7415907498278500 0.5038062029585934

0.5689248198578238 0.3244600767854510 0.3154045434203396

0.5143228738223937 0.2714111358696005 0.3687871702879256

0.5599761792191881 0.3129630293122776 0.4473514630445115

0.5010589621657392 0.2505790255469854 0.5039886500114482

0.5618011254905810 0.3129681859998865 0.5678029189632524

0.5001624488698551 0.2562544969854099 0.6271925265315969

0.5626725043490771 0.3178484428669697 0.6858401953438031

0.4940997404703314 0.3769367831684418 0.2651446307235820

0.5587047227756893 0.4365860910951230 0.3199902824788930

0.4995390617982770 0.3765820474868925 0.3834095545719789

0.5581315519184268 0.4377497956456421 0.4457675701810080

0.4987314683675968 0.3761375313338303 0.5058189117050800

0.5593701515846601 0.4386257741884672 0.5666384727111230

0.4995451744499693 0.3761479852203194 0.6271294215304749

0.5618610739282025 0.4364499429900580 0.6898248229629261

0.5006642529705138 0.3778941838383661 0.7468197574825900

0.4993150097953913 0.4980526672345253 0.2600927379452856

0.5598580959009472 0.5588870952901011 0.3202187516777856

0.4986791894955433 0.4980283298163700 0.3835462883004734

0.5591631684543025 0.5583035018527905 0.4446836964506983

0.4989672244606897 0.4984739748884884 0.5063024344469488

0.5603005716441136 0.5589731920065184 0.5654150225240112

0.4982524796195733 0.4992733336821569 0.6283173011077695

0.5612744679010134 0.5591413989745344 0.6891686699214177

0.4990983889208995 0.4961225804674605 0.7466938511834504

0.4986346826725404 0.6165372691157630 0.2632252494579415

0.5678234303750764 0.6747295204803654 0.3278682616842223

0.4991984064203452 0.6195970637249685 0.3826200014776171

0.5593371653275772 0.6804051098839258 0.4415811859180020

0.5004815984934913 0.6209519998436521 0.5045691018626038

0.5618721656509424 0.6830286004085580 0.5662955203153974

0.4998727770607710 0.6197210458890382 0.6256920630415718

0.5569813786434202 0.6764351173792530 0.6867776304606766

0.4974987770286211 0.6043971467170741 0.7539026435075198

0.5023457236505138 0.7390443436675539 0.3844203294550836

0.5021644642548294 0.7414426265753475 0.5027131477229250

0.5021925081475064 0.7406035332278457 0.6232642132946121

0.6804720140608745 0.3223457652135728 0.4459433833151319

0.6234267746052520 0.2588787259670589 0.5065197480491307

0.6845794004010837 0.3267727625615733 0.5603909444161441

0.6771033194511620 0.4373459924701883 0.3288424120260565

0.6146105032284944 0.3761172089373747 0.3867159667813593

0.6800636236993813 0.4366625134800607 0.4426885741741741

0.6188481057401213 0.3782989510394060 0.5050268332891170

0.6837294813502361 0.4362932061621580 0.5673442673478899

0.6203382421878487 0.3787529042360243 0.6244774399299381

0.6798777918853582 0.4373150484970721 0.6860711964114500

0.6190321122745613 0.4972415692281230 0.2637323175346095

0.6804599332936869 0.5524144299567175 0.3313814175942346

0.6172916090630942 0.4965613373941198 0.3849160495267138

0.6819550640737232 0.5599583407917842 0.4427143907547790

0.6220659516849981 0.4987051887099754 0.5060373526586294

0.6826148869157597 0.5608720984246359 0.5685041714346325

0.6208132917071567 0.4987166275617571 0.6274850802062852

0.6805568024956279 0.5593829646536158 0.6866544683878558

0.6174136304472498 0.5009825000048049 0.7476026870921074

0.6177416691112568 0.6134914138825149 0.3852204291379502

0.6769803913458572 0.6755757452761969 0.4336139252028282

0.6214431174033226 0.6189401410782467 0.5044653432698163

0.6790060019820652 0.6749956919059870 0.5673233920956985

0.6189165855716364 0.6187847136368486 0.6264338444374583

0.6247540833894439 0.7327429638211038 0.4951691868095456

0.7534573881077139 0.4020954930041677 0.4956933133545486

0.7524507110085412 0.4897866951752168 0.4075285347407497

0.7419394936433981 0.5016520673791177 0.5077545556496829

0.7390164102946842 0.4977375676219786 0.6248364478707359

0.7358647659735952 0.6205570808882445 0.5047006280916053

Fe86

Fe

1.00000000000000

20.0000000000000000 0.0000000000000000 0.0000000000000000

0.0000000000000000 20.0000000000000000 0.0000000000000000

0.0000000000000000 0.0000000000000000 20.0000000000000000

Fe

86

Direct

0.3284874635085140 0.3223315605528828 0.3392550857150805

0.3290219562215919 0.3229900568591332 0.4656109417861440

0.3295311283694915 0.3281733370460445 0.6040436593914126

0.3235643590983537 0.4659846579275235 0.3270804889427492

0.2566748742012449 0.3956194296781850 0.3961190256937692

0.3234958406511173 0.4644325733012060 0.4649227488703628

0.2577115965208926 0.3925749097982839 0.5299474220344794

0.3238892764919343 0.4638634717595856 0.6056464633614586

0.3303720619035952 0.6010830377542981 0.3267227890507668

0.2556218001143457 0.5378110361632028 0.3940881491018878

0.3247333442216228 0.5987795460661423 0.4658722961969349

0.2542648244236245 0.5330198547548478 0.5338654627287968

0.3233589065672786 0.6042445231670595 0.6030929197181770

0.2793162036492414 0.6852578874947836 0.4025546523263800

0.3402584834108635 0.7563313081315116 0.4725716800983503

0.2555884614224458 0.6661524752744833 0.5275634317671625

0.3276596790474834 0.7362792271534139 0.5949791464986380

0.4673459237161039 0.3259859594334392 0.3260818907027915

0.4104270397709615 0.2496983738411245 0.4003200628295415

0.4667569172911460 0.3252091903966700 0.4658854170880209

0.3995614282644003 0.2552199241144230 0.5365497611457483

0.4690779950072077 0.3234532793076040 0.6053852711919200

0.3929262695905927 0.3937162667216545 0.2591526571487756

0.4640592012896335 0.4648973577519345 0.3217554517968035

0.3966318133738233 0.3962517045374221 0.3963895363402855

0.4658640388125536 0.4652287909455308 0.4661090212244868

0.3968235604091292 0.3955446668272719 0.5359854443422758

0.4658056749415107 0.4647934182908243 0.6065818701366729

0.3996199255399986 0.3959283896032532 0.6754450822981640

0.4671492726360954 0.4676595607362936 0.7458284708461028

0.3933357412205701 0.5317958862437976 0.2550363957840836

0.4629003627638384 0.6056541623415651 0.3231466157934881

0.3971286344172808 0.5338240131105959 0.3951471361221642

0.4665472451741011 0.6040800174148278 0.4640961277764061

0.3949362411141902 0.5330522763360535 0.5360043572895964

0.4644096926443628 0.6033997302161582 0.6045902550376601

0.3942165249916980 0.5353753555645625 0.6769761324708083

0.4668733811709120 0.6046004264048177 0.7452192037476439

0.3945077523678332 0.6789568045863791 0.4018494357329940

0.4691733102111085 0.7464530775535193 0.4720359712073192

0.3973600641420986 0.6705551423545700 0.5325771577221509

0.4606914592247704 0.7434450607091775 0.5998003419567361

0.3965829209487282 0.6717643241527402 0.6733409224585084

0.6020945426834324 0.3316620094246672 0.3215450920341185

0.5395061839275146 0.2594418294245265 0.3942375934194562

0.6040389404811430 0.3284249962562649 0.4644999202157108

0.5347758935233630 0.2532776588312379 0.5249753372170702

0.6074530754796659 0.3259742571277426 0.6024533856064535

0.5288615809878046 0.3949653981496562 0.2530363850482258

0.6028514983832314 0.4649238126027537 0.3199129119705756

0.5346310648004327 0.3973396219765545 0.3932131943238903

0.6037359696584842 0.4659957555623639 0.4647977747549910

0.5347375480697373 0.3952872277316012 0.5348527998845533

0.6037176638863420 0.4663276184319238 0.6068305273246410

0.5368955771726612 0.3966831171227034 0.6784153624680000

0.6044121530583368 0.4715519603293479 0.7477703943920031

0.5294139371171871 0.5339334304105928 0.2530510671032567

0.5990507228282315 0.6015912065496286 0.3233419368884692

0.5339288963196498 0.5339409882657363 0.3927096186188300

0.6030988463198739 0.6040606794609855 0.4634362009008181

0.5348699474459061 0.5338457451092934 0.5342939722681201

0.6047881450185482 0.6041183969431727 0.6033545160443115

0.5351348364462631 0.5360370984100759 0.6768233522981876

0.6035075194245250 0.6082956025488935 0.7436670999867141

0.5336187606947697 0.6763741048673877 0.3953803840482573

0.6037858815478347 0.7445383689932570 0.4673350337939221

0.5347758573438383 0.6752051533643951 0.5355664030323387

0.5984504715602286 0.7453112931923924 0.6050110735814093

0.5325954378398535 0.6752696830811150 0.6740667004535875

0.6719385612823936 0.2669460122243900 0.3934266981751776

0.7383715928421188 0.3281140387708784 0.4733531191320371

0.6655034390692287 0.2492785108811277 0.5229178673334066

0.7233643606933968 0.3238908110638276 0.6029241828199559

0.6755822368867000 0.3973748113495042 0.3953428394217603

0.5347758573438383 0.6752051533643951 0.5355664030323387

0.5984504715602286 0.7453112931923924 0.6050110735814093

0.5325954378398535 0.6752696830811150 0.6740667004535875

0.6719385612823936 0.2669460122243900 0.3934266981751776

0.7383715928421188 0.3281140387708784 0.4733531191320371

0.6655034390692287 0.2492785108811277 0.5229178673334066

0.7233643606933968 0.3238908110638276 0.6029241828199559

0.6755822368867000 0.3973748113495042 0.3953428394217603

0.7462233461277841 0.4623798387160850 0.4684412926687991

0.6740534496941355 0.4010209740859403 0.5335430691097554

0.7403590038600277 0.4632025388342218 0.6070908576937871

0.6704419587455451 0.4011457133422837 0.6791180910261884

0.6722317122372544 0.5307616026756481 0.3954394230186729

0.7418194456326043 0.6040779864356476 0.4669621045859230

0.6752408930751852 0.5336092462321065 0.5332343366556132

0.7465174534212725 0.5958553955372731 0.5991121939840927

0.6745877390860430 0.5365910022718945 0.6769228790723538

0.6698073081344944 0.6708436676139312 0.3977155888933003

0.6744170627104900 0.6749451991384677 0.5355484745790272

0.6705468336241539 0.6741135842797008 0.6691025906785726

Fe

1.00000000000000

20.0000000000000000 0.0000000000000000 0.0000000000000000

0.0000000000000000 20.0000000000000000 0.0000000000000000

0.0000000000000000 0.0000000000000000 20.0000000000000000

Fe

59

Direct

0.2908443827639213 0.4283006039212983 0.4335780383828135

0.2894074391972040 0.4312869089269964 0.5713132184429113

0.2907454768444199 0.5666761878867558 0.4334245506231161

0.2895854110323811 0.5666090480337430 0.5695279120769567

0.4315343624764061 0.2920681784817186 0.4298225587401850

0.4336990172534838 0.2904280961744081 0.5661914247484151

0.4339638756460005 0.4343075716780569 0.2872419534762696

0.3642930874857674 0.3647375567806176 0.3625879165950861

0.4302289218539941 0.4317923342011625 0.4299731433480727

0.3594875624877290 0.3597488869302459 0.5005325595368476

0.4299910753694132 0.4292612904586876 0.5693810730359818

0.3617832202555324 0.3635748900718445 0.6379241685760634

0.4294799312707405 0.4314220570255305 0.7094980849690379

0.4304281453068934 0.5690603125842851 0.2915178992913247

0.3585229950402431 0.5002649030717170 0.3617727629441755

0.4286567366215104 0.5690029043226452 0.4307965527408437

0.3576280204960957 0.4993363056763593 0.5009555579216213

0.4302523632549660 0.5697904250851746 0.5700462573468257

0.3595025071531029 0.4991534279209139 0.6400526472704232

0.4291523021059657 0.5668702803421334 0.7105643123113631

0.3631897595910771 0.6369907379930130 0.3632205114415875

0.4333829514636824 0.7115038514663209 0.4285285619282544

0.3629364292650323 0.6427807489331523 0.5018631300797289

0.4291268019595744 0.7095058962751424 0.5618469618978872

0.3605165141739963 0.6353728971162226 0.6383701965369165

0.5674972933571341 0.2924077808052732 0.4302481799064302

0.5706629516840112 0.2922242495604235 0.5659382419683145

0.5660918648490086 0.4382140567267678 0.2862960379864419

0.5035620216276929 0.3637984454865701 0.3590201817616707

0.5679588008893215 0.4311717128444342 0.4322760540404126

0.5002394207126802 0.3589671420711971 0.4981621331131205

0.5692324540590586 0.4321783276901476 0.5685626549437893

0.5013655184590006 0.3618578405020770 0.6396490205713319

0.5679729228500178 0.4330356026772140 0.7077675247267020

0.5698223578858241 0.5690560222126311 0.2897454765481181

0.5009382399143950 0.5012412899229429 0.3568992633830133

0.5623712210387244 0.5664455084281712 0.4328416789086850

0.4977237398267164 0.4995212967582682 0.5006695091863402

0.5684475912918046 0.5695735472184581 0.5678330220021183

0.4986671938895708 0.5004378220304920 0.6408919520153337

0.5660848102503991 0.5713504536597794 0.7076183069238775

0.5010816527458981 0.6393764685619296 0.3609784880521499

0.5699569130004742 0.7081309379351863 0.4301629812660323

0.5006273693987946 0.6426773799642161 0.4975745526394569

0.5679058808429714 0.7064156940570596 0.5672620511815551

0.4973418423974784 0.6414011218993841 0.6408267886821993

0.6418670572626911 0.3726274907203742 0.3618669698285513

0.7116334139922407 0.4356249881831904 0.4261929252506246

0.6386188669139353 0.3627203480396122 0.4989632353384689

0.5623712210387244 0.5664455084281712 0.4328416789086850

0.4977237398267164 0.4995212967582682 0.5006695091863402

0.5684475912918046 0.5695735472184581 0.5678330220021183

0.4986671938895708 0.5004378220304920 0.6408919520153337

0.5660848102503991 0.5713504536597794 0.7076183069238775

0.5010816527458981 0.6393764685619296 0.3609784880521499

0.5699569130004742 0.7081309379351863 0.4301629812660323

0.5006273693987946 0.6426773799642161 0.4975745526394569

0.5679058808429714 0.7064156940570596 0.5672620511815551

0.4973418423974784 0.6414011218993841 0.6408267886821993

0.6418670572626911 0.3726274907203742 0.3618669698285513

0.7116334139922407 0.4356249881831904 0.4261929252506246

0.6386188669139353 0.3627203480396122 0.4989632353384689

0.7066556125279613 0.4342635879808181 0.5656743590482247

0.6393387896418088 0.3646322628384034 0.6362520908785116

0.6402164317755633 0.5043190989914587 0.3646544606949476

0.7198674340200505 0.5658071646058536 0.4325654652067573

0.6426430742051441 0.5028898066991129 0.4956687536840347

0.7093772400853658 0.5726186259896073 0.5639893796128402

0.6374861432656502 0.5016275683274340 0.6369962396978329

0.6358446297619926 0.6245816101696559 0.3736154144089691

0.6414441522459221 0.6344452859360413 0.4933657406362687

0.6324615785951819 0.6379336960344359 0.6365599737050974

Fe27

Fe

1.00000000000000

20.0000000000000000 0.0000000000000000 0.0000000000000000

0.0000000000000000 20.0000000000000000 0.0000000000000000

0.0000000000000000 0.0000000000000000 20.0000000000000000

Fe

27

Direct

0.4618529268875897 0.4551516838464301 0.4623205995734447

0.3857574008500955 0.3702233518016096 0.4653366986282646

0.4388140586222651 0.4142459845727681 0.5746061560502486

0.3750706288401596 0.4559326787390382 0.3834604769656391

0.4200459430175825 0.5629760360181865 0.4308458209657635

0.3563966087233278 0.4872602336295543 0.5109493088013460

0.3831740879135546 0.6145856798021890 0.5355999808530303

0.3843022007656294 0.5114138637194026 0.6268661927326507

0.4209958555636144 0.6819758061407925 0.4434892787577497

0.4642887094810659 0.3756133029546335 0.3761334281863544

0.5707763448385097 0.4258125270720453 0.4238108652269110

0.5077886133492534 0.3475307468659861 0.4939773448996841

0.5488559495018552 0.4535643281777876 0.5376277076529546

0.5404641375654535 0.3678051725521135 0.6176699149401325

0.4967514198861688 0.4960351518313993 0.3559389269521039

0.5431442538641528 0.5402124530945939 0.4581146186204447

0.3750706288401596 0.4559326787390382 0.3834604769656391

0.4200459430175825 0.5629760360181865 0.4308458209657635

0.3563966087233278 0.4872602336295543 0.5109493088013460

0.3831740879135546 0.6145856798021890 0.5355999808530303

0.3843022007656294 0.5114138637194026 0.6268661927326507

0.4209958555636144 0.6819758061407925 0.4434892787577497

0.4642887094810659 0.3756133029546335 0.3761334281863544

0.5707763448385097 0.4258125270720453 0.4238108652269110

0.5077886133492534 0.3475307468659861 0.4939773448996841

0.5488559495018552 0.4535643281777876 0.5376277076529546

0.5404641375654535 0.3678051725521135 0.6176699149401325

0.4967514198861688 0.4960351518313993 0.3559389269521039

0.5431442538641528 0.5402124530945939 0.4581146186204447

0.4616135329979481 0.5344009270639948 0.5486123487725597

0.5833091799225354 0.5644279138974369 0.5745738403715931

0.5116666027353407 0.4823991775191536 0.6500256652630930

0.5133214318805526 0.6260006337957507 0.3899771339850731

0.5031111860449926 0.6440198941908538 0.5150709684703392

0.4858058094739279 0.6053305746064899 0.6328608362694182

0.6276191961224399 0.3763544270710627 0.5302163513084230

0.6181784257038867 0.5267087189349864 0.3752851092610927

0.6507121143118172 0.4949558329040351 0.4921979756218273

0.6295105481340291 0.4617110045836075 0.6218796649040423

0.6166728330022595 0.6233518946140771 0.4725527859658235

Fe15

Fe

1.00000000000000

20.0000000000000000 0.0000000000000000 0.0000000000000000

0.0000000000000000 20.0000000000000000 0.0000000000000000

0.0000000000000000 0.0000000000000000 20.0000000000000000

Fe

15

Direct

0.4324298635434803 0.4310919158601191 0.4312027230386640

0.4310958455153989 0.4327089532813596 0.5689301366496735

0.4313527584938396 0.5688605556914758 0.4323747214266259

0.3648339659405124 0.5025861755844508 0.5023356782364023

0.4278227368259100 0.5727046255514379 0.5722995212130466

0.5721801552007069 0.4272995713693464 0.4277017527172540

0.5020935954948680 0.3646465366122390 0.4978583615820945

0.5686439570476934 0.4311375515830062 0.5676245821476171

0.5023677256546497 0.4974112503323445 0.3646920469636467

0.5689065589189432 0.5672867996887533 0.4310697810078321

0.4999986416327555 0.5000066296968613 0.5000016638389915

0.5675671196933405 0.5689111433488845 0.5687992400345943

0.4976334805027379 0.5025846828251272 0.6353077751153960

0.4979074553367279 0.6353503793176472 0.5021333008208214

0.6351661401984356 0.4974132292569608 0.4976687152073405

Fe9

Fe

1.00000000000000

20.0000000000000000 0.0000000000000000 0.0000000000000000

0.0000000000000000 20.0000000000000000 0.0000000000000000

0.0000000000000000 0.0000000000000000 20.0000000000000000

Fe

9

Direct

0.4805377557046400 0.4476393659728472 0.4155179514729951

0.3793554241207792 0.4872971690078413 0.5640062369076595

0.4089334451752855 0.5285033104097359 0.4526770740450130

0.4665837490961102 0.5743435630026458 0.5472680748000295

0.5910656395608654 0.4767301713507912 0.4500500320335504

0.5891360374107519 0.4212426028682359 0.5508359436124157

0.5172175429465166 0.5654822266826000 0.4433721793159488

0.4863964487054068 0.4612909527198800 0.5277623287607084

0.5807739572796444 0.5374706379854370 0.5485101790516868

Fe13O13 on N4-C initial

C N Fe O

1.00000000000000

12.3468564999999995 0.0000000000000000 0.0000000000000000

0.0000000000000000 12.8312298271967009 0.0000000000000000

0.0000000000000000 0.0000000000000000 20.0000000000000000

C N Fe O

54 4 13 13

Direct

0.0090010391939507 0.9528738296625880 0.4866166169826899

0.0089699049783998 0.0639196179544921 0.4870278408830978

0.1089141235512117 0.1195428512623201 0.4862160767768351

0.1085430688062868 0.2303696582222983 0.4860654447247217

0.0089805732945766 0.2860031777189561 0.4874417106686948

0.0090045203673729 0.3974618629543293 0.4873378078764055

0.1087138718494129 0.4530077544145508 0.4855825367473969

0.1087131138144078 0.5639415929755649 0.4852928701701644

0.0089939742980834 0.6194586967349760 0.4866768008479265

0.0089972434439776 0.7309211199918171 0.4863327708708111

0.1085527901307284 0.7865513459668068 0.4849779099078728

0.1089481771513901 0.8973394642917841 0.4854343775900088

0.2092674834337739 0.9529965220316872 0.4844747060641943

0.2092825227550978 0.0638973097004151 0.4850316914385764

0.3092564378835614 0.1197608675122199 0.4840599234194115

0.3082632186305492 0.2313247500951421 0.4834729393649105

0.2084886116943064 0.2864468299209213 0.4842057639287213

0.2086178334807946 0.3973903400374034 0.4834318896780025

0.3106594166093311 0.4501469702692780 0.4783462445667915

0.3105958739600657 0.5667172464484103 0.4777420856534764

0.2086096232933147 0.6195933068902425 0.4827092668295927

0.2084365621213524 0.7305344838210681 0.4829130426648899

0.3081540444291822 0.7856393427788113 0.4816475367021632

0.3092168715888606 0.8972640144915295 0.4826889826044796

0.4093032040419870 0.9525946711530012 0.4826745149785345

0.4094989440319237 0.0646749002340334 0.4835071203958203

0.5088437987330064 0.1239202804897203 0.4854699830068769

0.5088978055188815 0.2360930201809270 0.4857490339519881

0.4059015802964006 0.2918176756815964 0.4815460367818835

0.4055088359013594 0.7249138721009514 0.4796451114974164

0.5088362901413271 0.7805738694050520 0.4843086069089478

0.5087952450076647 0.8933464659237697 0.4844564615187958

0.6084358706729577 0.9524546837493656 0.4863012838041980

0.6082529681922669 0.0646913494288325 0.4870438675765454

0.7086043383156134 0.1197701518308287 0.4880204082830888

0.7095979468620484 0.2313322266030046 0.4892547179986815

0.6118875008803715 0.2918336852291663 0.4901698810834748

0.7071679635350344 0.4501902170632762 0.4929809662302049

0.7072341132045620 0.5667080785973637 0.4920223392785215

0.6122381911190690 0.7247137583287884 0.4872929304824574

0.7096971153430929 0.7854513720127008 0.4863060022044280

0.7086795724171612 0.8970488567598566 0.4862646957043376

0.8086443043888150 0.9529148015208240 0.4870151505113913

0.8086019470449513 0.0638661351320938 0.4877591117576762

0.9089994982975934 0.1195108110482734 0.4879413768410631

0.9094027845787636 0.2303460674193669 0.4885128893477354

0.8094316271977923 0.2864071451836499 0.4895677136542559

0.8093489431812130 0.3973538136498656 0.4899126955265675

0.9092833919666952 0.4529907269475494 0.4884765320309407

0.9092819194650248 0.5639137922450668 0.4880421048186506

0.8093115458152418 0.6195061615217268 0.4886746528836254

0.8094096104607971 0.7304452453891245 0.4873425333420977

0.9093676179705265 0.7864799855581023 0.4869545824880872

0.9090234955897928 0.8972880529725544 0.4869157193723206

0.4036937760991563 0.3970727851898438 0.4759975422031248

0.4035429036462024 0.6197899350019622 0.4745690750393259

0.6140285570779834 0.3971812970607713 0.4947970811546036

0.6140622834910985 0.6197480732223453 0.4925466920232696

0.4288464087592667 0.7941078496933048 0.8868024759771870

0.3983536458174288 0.6348267585065495 0.7889748722285810

0.2936153924109031 0.8032764284306944 0.8019050224255356

0.3550425806326116 0.9931245268329127 0.8145848529392912

0.3440105894893601 0.7173485014868938 0.6831116079631555

0.4789882278797519 0.8301215584586952 0.7547382956304667

0.5788472345735958 0.6821111470255636 0.6916781102689065

0.3206297849865719 0.9310675193565042 0.6830926865850514

0.5529614328833203 0.9926726444020940 0.6909871000472563

0.5812368642210977 0.6892199265082919 0.8153788176638800

0.6859100286164922 0.8414086009640299 0.7132789813085778

0.5775302527595475 0.9790708055656772 0.8121396103715989

0.5076149858188458 0.8234843099384499 0.6218709316691186

0.4673525379393560 0.6504857150744161 0.8769109504287969

0.3100294178289019 0.8923845515191183 0.8764334696480868

0.2591960345029457 0.6798092297330204 0.7570101749558349

0.5741276922596621 0.8317028854934388 0.8450654979001490

0.4426921538680570 0.9643578605037180 0.6301876950753288

0.5321755691863711 0.5830059893186812 0.7561413296578228

0.3057565437587386 0.0424464412612805 0.7355044792743755

0.4582491612793687 0.6758453870092811 0.6316569555039214

0.6829929885130336 0.9870087144131824 0.7388184210413326

0.4704297684616168 0.0595075395842670 0.8483132254952418

0.6538209929293392 0.7703215712665833 0.6307766615218322

0.6945118532466087 0.7048450185590437 0.7545413666142911

0.2718236851076854 0.8201541371946373 0.6386376691767431

Fe12O13 on N4-C final

C N Fe O

1.00000000000000

12.3468564999999995 0.0000000000000000 0.0000000000000000

0.0000000000000000 12.8312298271967009 0.0000000000000000

0.0000000000000000 0.0000000000000000 20.0000000000000000

C N Fe O

54 4 13 13

Direct

0.0097903338116909 0.9577186974822024 0.4875461449952043

0.0097583674701053 0.0685632781736012 0.4878230783272037

0.1101732049314139 0.1237459317274254 0.4871972821563175

0.1095885436073879 0.2348042727386075 0.4876116396295454

0.0097520544604478 0.2902725122601540 0.4887931855127465

0.0097722460997005 0.4015066048148189 0.4891368370283401

0.1080408445622791 0.4578624867231621 0.4877536561145021

0.1080923926515998 0.5686303958368999 0.4874449665716866

0.0097977811131119 0.6249189387047820 0.4885864348995301

0.0098273354377727 0.7361148953950115 0.4878940873420151

0.1095995290825423 0.7915333736922857 0.4864497199177336

0.1101994080189728 0.9025108763496942 0.4864926064843200

0.2109364090702453 0.9572487021729924 0.4851770601811247

0.2108513544703487 0.0690049790357651 0.4857706144354599

0.3109248939804882 0.1253835761386509 0.4849694997522121

0.3099401494356383 0.2376613274409352 0.4853940719815737

0.2087927003082367 0.2908039591513205 0.4864893300571830

0.2069682317928899 0.4019070192160013 0.4861680341960370

0.3046090144902098 0.4572600716171430 0.4841323675340866

0.3046036723919224 0.5693325910910462 0.4828632824588759

0.2069443816338182 0.6246017435684158 0.4850240511153321

0.2087858949674736 0.7355811815979892 0.4846649034665984

0.3098130029504506 0.7886598146227849 0.4828531613933072

0.3109558614961758 0.9008762538131621 0.4831827622608557

0.4111011296080127 0.9565580578924964 0.4825969707214908

0.4110534093748309 0.0697125991608499 0.4839109130544413

0.5096670200863621 0.1286561589510925 0.4851696648537112

0.5096989863847415 0.2429041402480763 0.4858857738348151

0.4080326155031702 0.2981439868877545 0.4850130707006121

0.4076896589309155 0.7280052594075249 0.4818540769181364

0.5097274637269908 0.7833875810794951 0.4835445419133094

0.5097368224111951 0.8978344274129467 0.4828334013776474

0.6083410341100109 0.9564669982604908 0.4847581755526359

0.6083438144941137 0.0698030386330530 0.4857949897035318

0.7084971290888080 0.1254615514849565 0.4873271664997602

0.7094575814420504 0.2377615768179355 0.4881221153449533

0.6113202855355799 0.2982074218231335 0.4874254357729363

0.7147780235053331 0.4572741703183374 0.4891361188123216

0.7148947851323901 0.5692562788584927 0.4883662926598780

0.6117086944615727 0.7280694821891225 0.4848074147458578

0.7097229721476978 0.7886214900554540 0.4858226120984965

0.7085927884361717 0.9008016015641545 0.4859259126004312

0.8086137025542983 0.9572646327049060 0.4873207054049782

0.8086182680500170 0.0690214869244452 0.4878029348296462

0.9093332619206432 0.1237507084729339 0.4883787366770825

0.9099099600037688 0.2348225344397571 0.4890080453981167

0.8106647375325921 0.2908414724163204 0.4892490320423210

0.8124881359215351 0.4019324940214197 0.4898138046270540

0.9114488204530464 0.4578530378754873 0.4898962001358738

0.9114832516534794 0.5686189013058370 0.4895710015041383

0.8125539259387984 0.6245452311498833 0.4888377075529182

0.8107291842701579 0.7355600955086924 0.4876148644418474

0.9099559712590743 0.7915177661005847 0.4879371558643266

0.9093743031927648 0.9025303397633296 0.4877294193201448

0.4028468526318430 0.4065467296632405 0.4840942078370063

0.4025212676889769 0.6200264542624391 0.4805950988221431

0.6164375097950134 0.4066477209825976 0.4880949489301212

0.6167660093894051 0.6199563195999535 0.4861197798441579

0.4267438266601976 0.8070987410207026 0.8852142323276595

0.4273577966695791 0.6327626276622865 0.7971806166450530

0.2895902390634993 0.7872179284809595 0.8056840998766835

0.3390892917342182 0.9877634400305521 0.7990281787028696

0.3351627264020646 0.6829905148298552 0.6915447276240223

0.4680894919273859 0.8189631828873297 0.7530736607903973

0.5094343370942324 0.5135731866853924 0.4865086993640504

0.3120947367270355 0.8939737297332311 0.6759519897183489

0.5445324165921124 0.9588830891288096 0.6766063457882202

0.6029554002848163 0.7104998016059256 0.8178317658625998

0.6805531194803703 0.8299355776960451 0.7057914504458445

0.5584103092975833 0.9911238186461924 0.7962692197120388

0.5123133043856208 0.7559971666883076 0.6304750110006627

0.4920564685701416 0.6762560116353871 0.8857979713679613

0.3008249387788917 0.8975153426557096 0.8681472996929631

0.2761811900825342 0.6501622575382607 0.7761228522485623

0.5606294438535331 0.8564954448557736 0.8415467922046120

0.4374767797631380 0.9021655277608864 0.6225554660698843

0.5656456082827929 0.5924463145911022 0.7769433870260626

0.2967214139241856 0.0184619408014363 0.7145968141160770

0.4207970885476590 0.6435510605851349 0.6247499279338314

0.6692941780682966 0.9770730664262725 0.7272546726320940

0.4470433280600706 0.0722861225052759 0.8247638147453418

0.6635048643100327 0.7495155066928967 0.6350622240750142

0.7230355850586518 0.7492347798242845 0.7744226469380754

0.2476843027716618 0.7792879379139761 0.6494065778975783

Fe13O11

Fe O

1.00000000000000

15.0000000000000000 0.0000000000000000 0.0000000000000000

0.0000000000000000 15.0000000000000000 0.0000000000000000

0.0000000000000000 0.0000000000000000 15.0000000000000000

Fe O

13 11

Direct

0.4679269304533662 0.3392737671881250 0.5189430346937343

0.4842767928681440 0.4000701258326629 0.3373732862021086

0.3516034806214918 0.4483914792838277 0.4402722090287199

0.3906013315734000 0.4771205550218625 0.6039811225182348

0.4266573154076738 0.5717330951189936 0.3373408075220965

0.4973199942972713 0.5079760569003398 0.4785993939126030

0.6258373941999196 0.5383299333473133 0.3785450173070117

0.3884767531567519 0.6336321178176618 0.5142080989435129

0.5127458895577703 0.6125806970424179 0.6255555971459827

0.6077758047793225 0.3850150679514428 0.4425265074669227

0.6574164499655026 0.5547010239975307 0.5378719707957842

0.5608436705890796 0.4484824447484286 0.6218779994301329

0.4679269304533662 0.3392737671881250 0.5189430346937343

0.4842767928681440 0.4000701258326629 0.3373732862021086

0.3516034806214918 0.4483914792838277 0.4402722090287199

0.3906013315734000 0.4771205550218625 0.6039811225182348

0.4266573154076738 0.5717330951189936 0.3373408075220965

0.4973199942972713 0.5079760569003398 0.4785993939126030

0.6258373941999196 0.5383299333473133 0.3785450173070117

0.3884767531567519 0.6336321178176618 0.5142080989435129

0.5127458895577703 0.6125806970424179 0.6255555971459827

0.6077758047793225 0.3850150679514428 0.4425265074669227

0.6574164499655026 0.5547010239975307 0.5378719707957842

0.5608436705890796 0.4484824447484286 0.6218779994301329

0.5270430196076674 0.6669273045140258 0.4293327407315743

0.5199401966455568 0.2994275534894157 0.4077733350841513

0.3473067334014601 0.3758244453793580 0.5455331351171395

0.3699232063532918 0.4592068924060300 0.3166810343784832

0.5915343960194193 0.3430763546440900 0.5638757808234161

0.5025547153320460 0.7092077471918188 0.5434175562081407

0.6078926471739692 0.4287322917967629 0.3209691279859967

0.3802145214460211 0.5950830637631128 0.6371341741026129

0.5476026236101319 0.6145275862051207 0.3144513135534837

0.6493089164204306 0.6444093251213161 0.4438941896759303

0.7016710339819170 0.4685218943702552 0.4555482666747608

0.4019291428445868 0.6811825554654334 0.3984483887063064

Fe12O11

Fe O

1.00000000000000

15.0000000000000000 0.0000000000000000 0.0000000000000000

0.0000000000000000 15.0000000000000000 0.0000000000000000

0.0000000000000000 0.0000000000000000 15.0000000000000000

Fe O

12 11

Direct

0.4736736652730684 0.3450346444129693 0.5298579435935125

0.4845410786126934 0.4059789503538838 0.3397576598160725

0.3462690737997747 0.4462990715416453 0.4392555453371827

0.3732939342530344 0.4658283273141777 0.5957464335402705

0.4266882671625112 0.5709096054102076 0.3447346785220731

0.4947813154551142 0.5035088300639998 0.4886581938190844

0.6266069225703674 0.5403090227401179 0.3682045552880808

0.3629485188643216 0.6140548991142936 0.5070870195622064

0.4736736652730684 0.3450346444129693 0.5298579435935125

0.4845410786126934 0.4059789503538838 0.3397576598160725

0.3462690737997747 0.4462990715416453 0.4392555453371827

0.3732939342530344 0.4658283273141777 0.5957464335402705

0.4266882671625112 0.5709096054102076 0.3447346785220731

0.4947813154551142 0.5035088300639998 0.4886581938190844

0.6266069225703674 0.5403090227401179 0.3682045552880808

0.3629485188643216 0.6140548991142936 0.5070870195622064

0.4823123145942674 0.6174953031446861 0.6054120770397518

0.6086999640929847 0.3947129625466914 0.4462848479718247

0.6547444582519688 0.5851558917725157 0.5307803055123372

0.5414478793373618 0.6580907249691066 0.4329179702931754

0.5165109424703260 0.3087819131694900 0.4186641032020759

0.3484454303262012 0.3615553267258136 0.5385287246088524

0.3701324273685174 0.4604997937648676 0.3162931415468861

0.5858193648359347 0.3587711006713619 0.5630149659547854

0.5740654488774126 0.6828816643629949 0.5628685451487456

0.6083176647523558 0.4261116594711183 0.3203687193829197

0.3661766600221483 0.5816835685466728 0.6347270931715698

0.5417160645583436 0.6136598581134933 0.3112062666200188

0.6645398365836499 0.6506001876988989 0.4150483606521576

0.6943158356061403 0.4831554744907791 0.4657868145700732

0.4115122220486001 0.6798721534491250 0.4170721234250436

N4-C

C N

1.00000000000000

12.3468565000000012 0.0000000000000000 0.0000000000000000

0.0000000000000000 12.8312298271967276 0.0000000000000000

0.0000000000000000 0.0000000000000000 20.0000000000000000

C N

54 4

Direct

0.0000675837204440 0.9492810243997951 0.5000000410542427

0.0000693987002265 0.0603521742555344 0.5000001850441294

0.0999819274239400 0.1158282604323020 0.5000024129081098

0.0995673470572029 0.2266321585743256 0.5000020583758982

0.0000701741123521 0.2822315560533966 0.5000006783176403

0.0000765701218057 0.3938007196989872 0.5000009845823072

0.0996196204754440 0.4493187636628733 0.5000021424145871

0.0996190345733723 0.5603085903953371 0.5000016584531011

0.0000705227078512 0.6158193025777763 0.5000003530336343

0.0000693677467041 0.7274030332588595 0.4999999417155010

0.0995728710350292 0.7829968821939346 0.5000012884094218

0.0999822927201990 0.8937909708409180 0.5000017990077410

0.2003816946101153 0.9493819938484052 0.5000039343736731

0.2003794122560549 0.0602387089408225 0.5000043167227588

0.3003716864787265 0.1159710599393762 0.5000031505983742

0.2993072035477624 0.2275906565622918 0.4999989063098340

0.1995267844853856 0.2826572636469464 0.5000005904105222

0.1994265179861401 0.3935915531840450 0.4999989449060251

0.3014630237401690 0.4464089616912403 0.4999826064530382

0.3014510276894384 0.5631929595438265 0.4999841020819886

0.1994203038601441 0.6160189277947552 0.4999990342820411

0.1995262842116787 0.7269657851500781 0.5000008304838133

0.2993169756298713 0.7820224607389505 0.5000004801549167

0.3003636432556860 0.8936245624994541 0.5000032415248370

0.4006793565583564 0.9486483110503414 0.5000013332847502

0.4006746569546493 0.0609146351798131 0.5000009814274406

0.5000696109205265 0.1202404483419528 0.4999990086870696

0.5000711073639863 0.2323891335626769 0.4999963389846229

0.3968477319482133 0.2881033179590489 0.4999877635704024

0.3968464338675730 0.7214869514867175 0.4999919511544491

0.5000736498551106 0.7772027971425004 0.4999996739366139

0.5000679691722425 0.8893234366459595 0.5000003750908348

0.5994614207099502 0.9486487713052796 0.5000000792765320

0.5994632252354606 0.0609139344531826 0.4999994492942292

0.6997685189208340 0.1159731735712626 0.4999974762117034

0.7008322936441331 0.2275923999105984 0.4999990124519940

0.6032967392115367 0.2881071503027144 0.5000045503069259

0.6986853197252358 0.4464104547119803 0.5000122208378954

0.6986919635664890 0.5631917693580064 0.5000124161332948

0.6032946543635660 0.7214853243078352 0.5000068751418212

0.7008240374862780 0.7820217518187889 0.5000004230663464

0.6997746380979493 0.8936249866001660 0.4999985001005740

0.7997600804482161 0.9493855545848859 0.4999976267487156

0.7997601920977928 0.0602422787685697 0.4999972617809689

0.9001562479183676 0.1158305435439217 0.4999984929932211

0.6997685189208340 0.1159731735712626 0.4999974762117034

0.7008322936441331 0.2275923999105984 0.4999990124519940

0.6032967392115367 0.2881071503027144 0.5000045503069259

0.6986853197252358 0.4464104547119803 0.5000122208378954

0.6986919635664890 0.5631917693580064 0.5000124161332948

0.6032946543635660 0.7214853243078352 0.5000068751418212

0.7008240374862780 0.7820217518187889 0.5000004230663464

0.6997746380979493 0.8936249866001660 0.4999985001005740

0.7997600804482161 0.9493855545848859 0.4999976267487156

0.7997601920977928 0.0602422787685697 0.4999972617809689

0.9001562479183676 0.1158305435439217 0.4999984929932211

0.9005738837734540 0.2266330184586423 0.4999990097927167

0.8006155215926967 0.2826566977639899 0.4999991429175097

0.8007213810008409 0.3935889225968054 0.5000002065632003

0.9005319538924041 0.4493142142662521 0.4999992187881901

0.9005273681272072 0.5603053833666536 0.4999991357351234

0.8007226302341479 0.6160169251242067 0.5000006062582258

0.8006178096546961 0.7269645935213163 0.4999997142102259

0.9005654215115868 0.7829958796591967 0.4999989228981434

0.9001554488149566 0.8937896361383508 0.4999986093172524

0.3943851270159016 0.3936040177815485 0.4999689041379582

0.3943794622237604 0.6159893734557258 0.4999741184220241

0.6057625074389463 0.3936058719007676 0.5000202511493228

0.6057631063007847 0.6159876603433005 0.5000218016096412

Fe-N4-C

C N Fe

1.00000000000000

12.3468565000000012 0.0000000000000000 0.0000000000000000

0.0000000000000000 12.8312298271967276 0.0000000000000000

0.0000000000000000 0.0000000000000000 20.0000000000000000

C N Fe

54 4 1

Direct

0.0000608475275854 0.9494448779136064 0.5000001076418670

0.0000603542580464 0.0602363582424204 0.5000003541919469

0.1004786873482999 0.1154221785154093 0.5000005801872405

0.0998954929638813 0.2264911205151492 0.5000014124751289

0.0000726156352258 0.2819384602898865 0.5000007239353152

0.0000845640706899 0.3930348150554934 0.5000011599908802

0.0983094372995481 0.4494166500995007 0.5000028730936066

0.0983044557111157 0.5602262061824845 0.5000024965447452

0.0000797381532038 0.6166202863947348 0.4999994861445956

0.0000708164447474 0.7277361607808004 0.4999989098384106

0.0998885480163239 0.7831740531910967 0.4999998211111176

0.1004753629209816 0.8942307303996344 0.5000000058666956

0.2011038589431706 0.9489581368635029 0.5000019624857330

0.2010910360329774 0.0606544712211274 0.5000025956044922

0.3012269700503936 0.1169265078661326 0.5000045442087000

0.3001637489220244 0.2289874579648838 0.5000026965782993

0.1990859691493441 0.2824138194941241 0.5000010007023477

0.1971648422570311 0.3935677579485031 0.4999994963826921

0.2949982513832339 0.4487736060355871 0.4999861911892012

0.2949905900112526 0.5608465289974092 0.4999884081215171

0.1971561745811314 0.6160670077317434 0.5000002345696970

0.1990572858664379 0.7272247364984565 0.5000016764790018

0.3001520595322305 0.7806001100837037 0.5000038234178490

0.3012184835307323 0.8926462392019161 0.5000044462503652

0.4015198154131951 0.9481828917273134 0.5000024233511482

0.4015072665988058 0.0613303729565899 0.5000023765744075

0.5000620285002100 0.1200103351871533 0.4999991943225268

0.5000675023391423 0.2342861222321631 0.4999959913889001

0.3983191794782144 0.2893560671434727 0.4999907667139283

0.3983148861769763 0.7202131190820324 0.4999959643586962

0.5000688750086582 0.7752309788694589 0.4999997244258267

0.5000617584717943 0.8894901902279813 0.5000009503893850

0.5986030055210065 0.9481816277043507 0.4999995556633436

0.5986154354407773 0.0613301542265674 0.4999990447005871

0.6988985952033104 0.1169244297387309 0.4999962500912858

0.6999738023731084 0.2289858980254351 0.4999954881403141

0.6018217939028006 0.2893556081917836 0.4999996061656375

0.7051723899543703 0.4487673375271655 0.5000095087762944

0.7051706373239810 0.5608414488108693 0.5000105164131341

0.6018296750823637 0.7202121772628516 0.5000032831503418

0.6999829011292888 0.7805997859508551 0.4999972611540855

0.6989045389821911 0.8926459344963362 0.4999975960348664

0.7990187980394268 0.9489569772917505 0.4999993914841383

0.7990336804483249 0.0606537780883230 0.4999985452688520

0.8996469534573137 0.1154224245329729 0.4999999844365419

0.9002466715864870 0.2264925343320741 0.4999997012346284

0.8010601015841416 0.2824119443867019 0.4999984677722082

0.6018296750823637 0.7202121772628516 0.5000032831503418

0.6999829011292888 0.7805997859508551 0.4999972611540855

0.6989045389821911 0.8926459344963362 0.4999975960348664

0.7990187980394268 0.9489569772917505 0.4999993914841383

0.7990336804483249 0.0606537780883230 0.4999985452688520

0.8996469534573137 0.1154224245329729 0.4999999844365419

0.9002466715864870 0.2264925343320741 0.4999997012346284

0.8010601015841416 0.2824119443867019 0.4999984677722082

0.8030063262309568 0.3935654671945201 0.5000010861792603

0.9018627676822533 0.4494183809116521 0.4999988297205746

0.9018557237140001 0.5602277707940004 0.4999989372879057

0.8029998647555558 0.6160653123863824 0.5000017336590474

0.8010806301336970 0.7272230518683429 0.4999998056466097

0.9002482837184047 0.7831713433178962 0.4999991931368584

0.8996523616822145 0.8942274506949062 0.5000004224173793

0.3927414491020258 0.3972290737325395 0.4999586515209558

0.3927348229123169 0.6123708798426790 0.4999651056647920

0.6074239088394364 0.3972271365354985 0.5000260040978262

0.6074275898140087 0.6123686767093446 0.5000289897691559

0.5000897966222553 0.5048159757123258 0.4999869636911498

MD: initial Fe13O13 on N4-C

C N Fe O

1

14.823447 0.000000 0.000000

0.000000 12.753356 0.000000

0.000000 0.000000 20.000000

C N Fe O

66 4 13 13

Direct configuration

0.12851061 0.79355794 0.45666623

0.12102461 0.90434715 0.46512927

0.20600068 0.95898159 0.47110968

0.20808315 0.07483875 0.47596765

0.12761623 0.12060886 0.49419875

0.11985521 0.23304046 0.49141988

0.20212558 0.29151590 0.47345404

0.20086006 0.39902434 0.47715895

0.11926751 0.45598500 0.48084712

0.11967557 0.56390368 0.46416672

0.20181084 0.62295847 0.46286970

0.20855130 0.73403389 0.45624719

0.28970151 0.79698963 0.46049450

0.29124711 0.90316373 0.46894339

0.37068520 0.96172671 0.47114274

0.37796763 0.07233907 0.47075634

0.29338056 0.12952207 0.46956201

0.28761056 0.23618863 0.47474435

0.37498933 0.28714910 0.47932231

0.36963420 0.40445145 0.47997343

0.28349708 0.45605128 0.47176827

0.28791367 0.56868113 0.46699829

0.37366431 0.63132263 0.47039950

0.36886994 0.73980133 0.46174732

0.46070563 0.78845457 0.46563210

0.45810306 0.89689121 0.47202177

0.54169066 0.96406583 0.47577889

0.54119224 0.06953294 0.47839534

0.45399662 0.13180425 0.47686267

0.45758813 0.56842253 0.47806813

0.54289787 0.61636477 0.47066356

0.54283480 0.72982051 0.46646374

0.61680160 0.79986918 0.46318343

0.61516273 0.90801433 0.45956198

0.70014965 0.95295443 0.46662304

0.70599486 0.07359777 0.48593029

0.62413551 0.12842201 0.48094928

0.71165923 0.28642671 0.48716076

0.71366567 0.41234628 0.48317410

0.62480848 0.56731617 0.47323940

0.70557195 0.62704630 0.47510220

0.69893339 0.73772015 0.46084009

0.78341052 0.79502091 0.44858077

0.78157945 0.90349887 0.45832032

0.86628539 0.94758925 0.47481138

0.87433536 0.05851221 0.48021235

0.79366426 0.12031811 0.48964033

0.78995856 0.23853648 0.49786205

0.87277880 0.29348891 0.49034000

0.86935698 0.40347010 0.49230765

0.79117720 0.47103953 0.49326491

0.79005061 0.58526255 0.48971717

0.87064627 0.63840718 0.47664694

0.86350366 0.73484490 0.44811363

0.96010061 0.78960892 0.44874198

0.95394419 0.89654669 0.47022026

0.03811466 0.95897821 0.47810829

0.03662231 0.06552897 0.50245375

0.95857742 0.11931471 0.49091661

0.95446229 0.23460027 0.49633373

0.03889855 0.29589235 0.49829719

0.03265948 0.40481478 0.48784624

0.95159957 0.46207911 0.48858583

0.95509778 0.57970778 0.48070615

0.03528937 0.62532318 0.45241127

0.04470276 0.73810205 0.44911833

0.45396302 0.23933969 0.48051476

0.44789635 0.45576207 0.47489833

0.63063546 0.23428421 0.47958263

0.63071167 0.45141575 0.47714935

0.35435056 0.79974932 0.88707422

0.33182828 0.64081576 0.78998861

0.24408205 0.80862943 0.80237689

0.29539452 1.00170811 0.81321895

0.28614723 0.72309191 0.68314922

0.39757915 0.83674525 0.75537578

0.48281074 0.68438010 0.69220444

0.26655427 0.93844877 0.68230843

0.46106158 0.99777228 0.69075032

0.48503237 0.69152818 0.81606260

0.57157852 0.84603752 0.71162085

0.48100248 0.98630960 0.81147910

0.42277126 0.82788916 0.62229323

0.38486564 0.65104203 0.87555924

0.25339633 0.89751220 0.87679176

0.21587932 0.68850960 0.75808780

0.48160454 0.83929636 0.84590375

0.36789980 0.97063474 0.62805311

0.44262484 0.58672416 0.75391223

0.25800741 0.04643738 0.73654091

0.38023166 0.67879894 0.63324055

0.57396378 0.99216498 0.73973197

0.38847123 0.05815193 0.84835515

0.54589210 0.77516007 0.63221159

0.58200822 0.70905904 0.75702596

0.22394145 0.82255194 0.63896098

MD: final Fe13O12 on Fe-N4-C

C N Fe O

1

14.823447 0.000000 0.000000

0.000000 12.753356 0.000000

0.000000 0.000000 20.000000

C N Fe O

66 4 13 13

Direct configuration

0.60471061 0.45830391 0.46647895

0.61108515 0.56598639 0.47496395

0.68983829 0.62364201 0.46973937

0.69025006 -0.26267815 0.47769643

0.60956899 -0.20397138 0.47662091

0.61398393 -0.09169706 0.47824104

0.68864050 -0.03390548 0.48058449

0.69168140 0.07436581 0.47187827

0.60385711 0.12552760 0.46951718

0.60935881 0.23975215 0.46709510

0.68939844 0.29518570 0.46938761

0.69401230 0.40873471 0.46583877

0.77670683 0.45248900 0.46910084

0.77592807 0.56779358 0.47016164

0.86131305 0.61959709 0.47004608

0.86456420 -0.27181670 0.48379006

0.77488978 -0.20938723 0.48542404

0.76802647 -0.09723076 0.48616450

0.85390725 -0.04292598 0.48898724

0.85748590 0.07024255 0.47745649

0.77432542 0.12375755 0.47445935

0.76854844 0.24220935 0.47237310

0.85639592 0.28810642 0.47127701

0.85997293 0.39720295 0.46688565

0.93998490 0.45036692 0.47282208

0.94877775 0.56632433 0.48182414

1.03042381 0.62157767 0.49263403

1.03154043 -0.25175832 0.49582886

0.94533541 -0.20661760 0.48652356

0.94533933 0.23089167 0.46634423

1.02652967 0.27938151 0.46409884

1.02667428 0.39244738 0.47891748

1.10458116 0.45348492 0.48716878

1.10980231 0.56217205 0.49461000

1.19153645 0.62958165 0.49842215

1.19412758 -0.25402256 0.50927308

1.11547410 -0.19837639 0.50975936

1.19744030 -0.03366952 0.52410033

1.19791240 0.07388216 0.51852754

1.11087975 0.22990968 0.49305565

1.19471764 0.29615726 0.49137917

1.18608322 0.41373103 0.49995330

1.27385431 0.46941745 0.49467597

1.28348112 0.58435311 0.49708862

1.36431849 0.63372973 0.49391638

1.36151429 -0.25796931 0.49467484

1.28128272 -0.20988071 0.50809176

1.28003883 -0.09538015 0.51125299

1.36087847 -0.04493525 0.49587650

1.35718809 0.07398074 0.49669823

1.28092775 0.12378837 0.50190992

1.27526439 0.24300960 0.50154763

1.35460438 0.29404107 0.48725263

1.35180679 0.40692139 0.48666695

1.43777903 0.46116583 0.47542829

1.44466570 0.56893153 0.48531115

0.52443729 0.62212409 0.48326266

0.52361021 -0.26317027 0.48583728

1.43935359 -0.20307585 0.49288575

1.44265672 -0.09828066 0.49519483

0.52616535 -0.04166387 0.47465286

0.51742235 0.07261276 0.47883042

1.43865554 0.12930198 0.48326155

1.44299090 0.24103135 0.47169074

0.52498670 0.29983942 0.46127352

0.52145848 0.41185385 0.47219168

0.93517272 -0.09558797 0.48475353

0.93658566 0.12810275 0.47439187

1.11679709 -0.08150130 0.51094412

1.11488345 0.12179745 0.50726419

0.06545336 1.23265997 0.73885019

-0.07136035 1.31575853 0.82309921

0.02489366 1.01646281 0.51264084

0.22407223 1.09724468 0.79225398

-0.26182390 1.11076567 0.65171530

0.04865263 1.10163717 0.65972260

-0.12802453 1.23207952 0.70456676

-0.09298641 1.01191417 0.66735891

-0.10124807 1.00777624 0.82868672

0.06594865 1.14906356 0.86577444

-0.26212815 1.12801451 0.77241183

0.07126996 1.00334272 0.76354023

-0.24736284 1.29762464 0.78790364

0.18075544 1.13703626 0.86470315

0.14787012 1.14417653 0.71069564

-0.20994956 0.98494654 0.65833816

-0.03116514 1.06510317 0.88430454

-0.06131938 0.96398729 0.74879771

-0.02163592 1.32160759 0.71900879

-0.01158218 0.02012061 0.59841118

-0.17536663 1.21223407 0.62765134

-0.22549234 1.01042136 0.81667957

0.19572282 -0.02866240 0.76471590

-0.17624041 1.31288092 0.86021806

0.03937304 1.28815625 0.84782115

-0.32488643 1.21428947 0.73108476
