# Supplementary material for: An in situ exploration of how Fe/N/C oxygen reduction catalysts evolve during synthesis under pyrolytic conditions
Source: Nat Commun. 2024 Jul 24;15:6229. doi: 10.1038/s41467-024-50629-x (PMC11266712; doi:10.1038/s41467-024-50629-x)
Supplement: Supplementary file 2 — Peer Review File [file 41467_2024_50629_MOESM2_ESM.pdf]

nature portfolio

Peer Review File

An in-situ exploration of how Fe-N-C catalysts evolve during synthesis under pyrolytic conditions

Open Access This file is licensed under a Creative Commons Attribution 4.0 International License, which permits use, sharing, adaptation, distribution and reproduction in any medium or format, as long as you give appropriate credit to the original author(s) and the source, provide a link to the Creative Commons license, and indicate if changes were made. In the cases where the authors are anonymous, such as is the case for the reports of anonymous peer reviewers, author attribution should be to 'Anonymous Referee' followed by a clear attribution to the source work. The images or other third party material in this file are included in the article's Creative Commons license, unless indicated otherwise in a credit line to the material. If material is not included in the article's Creative Commons license and your intended use is not permitted by statutory regulation or exceeds the permitted use, you will need to obtain permission directly from the copyright holder. To view a copy of this license, visit http://creativecommons.org/licenses/by/4.0/.

REVIEWER COMMENTS

Reviewer #1 (Remarks to the Author):

In the manuscript, the authors provided a visualized process for the conversion of the Fe2O3 particles into FeNx single atom site. The manuscript was well organized, and the research methodology could be helpful in studying similar catalysts. However, significant concerns of this work are the novelty and potential impact. Recently, similar results of using Fe2O3 as a precursor were discovered and reported to synthesize single Fe atom catalysts. The dynamic conversion of Fe2O3 nanoparticles to single Fe sites in catalysts was also elucidated. Such correlations between catalytic activity and heating temperatures were also studied in the literature. Furthermore, the presenting catalyst performance in both aqueous electrolyte and fuel cells is inferior to those recently reported. Therefore, this manuscript is not recommended for publication in the journal. Meanwhile, more detailed technical comments are listed below:

1. The activity and stability of PGM-free ORR catalysts have been continuously improved recently. The claim of "We recently reported a Fe/N/C catalyst with a performance of 710 mW cm-2 in H2/air PEMFC, which is the highest value yet." (Line 49) is not fair. Especially, when the test conditions are different, the peak power density will change significantly. A neutral statement should be made here. We encourage the authors to compare the fuel cell performance at practical operating parameters, such as current densities at 0.67, 0.7 or 0.8 V in H2/air at ambient pressure.
2. The explanation of the migration of the FeOx nanoparticles in the N-doped carbon is speculative and subjected to debate. The concept of "The surface carbothermic reaction produced an energy release that aided in the conversion process" was proposed as the driving force for the migration of the Fe-based nanoparticles. The explanation spread across the manuscript (e.g., Line 153, Line 169, Line 226). However, a carbothermic reaction is an endothermic reaction. How could this process supply energy to other processes and kick them off? The hypothesis lacks support from the model or simulation. It is improper to claim a counterintuitive concept without theoretical evidence to support the major findings in this work.
3. Regarding to the suggestions for the rational design of Fe/N/C catalysts, NH4Cl and NH4Br treatment (Line 289) have been reported in the previous literature and served a postal treatment to the raw catalyst. The suggestion does not match with the findings reported in this paper and undermines the significance of the paper.
4. Some typos in the paper. In Line 30, does the metrics mean "matrix"? Line 123, a comma before "at 350 oC" could be deleted.
5. In Supplementary Fig.1, please specify what species refers to the domain peak at 35.1 from 600Co to 900Co.
6. In this work, the only possible FeN4 site observed by the TEM is the images in Supplementary Fig.22, which are shown as bright dots. However, the bright spots are not the strong evidence of the FeN4 site. The EELS spectroscopy must be employed to demonstrate the universal existence of the FeN4 site.
7. As the author mentioned in Figure 5b, only 44% of Fe contents are converted into FeN4 site. The other Fe species are Fe or FeOx. However, the Fe and FeOx are unstable during the Fuel cell operation in the acid media. The durability result of the fuel cell based on Fe-N-C catalysts is necessary to illustrate the benefits or negatives of the existence of FeOx in the catalysts for ORR reaction.

Reviewer #2 (Remarks to the Author):

This manuscript delves into the in-situ observation and mechanistic exploration of the structural transformation occurring during the pyrolysis of Fe/N/C electrocatalysts. Such insights are crucial for advancing the efficacy of oxygen reduction reaction catalysts. With the potential to supplant platinum-based alternatives, research on Fe/N/C catalysts is important. The integration of machine learning

with theoretical computations in this work sheds light on the development of Fe species and the genesis of Fe–N4 sites. This methodological innovation bestows a fresh perspective and a novel toolset for probing Fe/N/C catalysts, thereby enriching the depth and breadth of the research. The authors articulate a strategy to modulate the size of iron oxide particles during pyrolysis, aiming to augment the conversion rate to Fe–N4 sites. This inventive approach holds guiding relevance for fabricating high-performance Fe/N/C catalysts.
The manuscript boasts a coherent structure, sound experimental design, meticulous data analysis, and comprehensive discussion, which collectively buttress conclusions of the manuscript. It is recommended for publication after minor revisions.
i) The authors should address the changes in specific surface area, pore structure, and carbon defects through the pyrolysis process.
ii) The influence of nitrogen in the carbon matrix warrants consideration; for instance, is it feasible to synthesize single-atom catalysts with carbon supports devoid of nitrogen doping?
iii) The authors need to provide 57Fe Mössbauer spectroscopy characterization to demonstrate the conversion trajectory of iron oxide nanoparticles.
iv) To substantiate the synthesis proposition of the manuscript, the fabrication of Fe/N/C catalysts via acid gas-assisted pyrolysis should be attempted, to validate the study’s conclusions.

Reviewer #3 (Remarks to the Author):

This work of Yin et al. presents interesting observations regarding the formation of iron single atom catalysts supported on carbon. Although numerous types of SACs have been demonstrated, their formation mechanisms have been elusive. More recently, there have been attempts to better understand the process during pyrolysis and the reviewer finds that this work performing in-situ TEM nicely complements the knowledge that has been collected so far in the field. Thus, the reviewer recommends the publication of this work after the following questions and concerns have been addressed.

1. LCF of XANES is misleading as it suggests that Fe-N4 sites are formed even as low as 500 C (24%) and increased minorly up to 900C (44%). It does question whether this method is accurate enough to be valid. This also means that Fe-N4 sites are formed during the fusion and growth stage, but the discussion around the first half of this manuscript based on TEM data analysis suggests otherwise. The paper should present a consistent picture of the data that is collected by various means.

2. How are the data related to Fig. 5b collected? FeN4 active sites are I assume by nitrite adsorption. But what about the inactive FeN4 and the inactive Fe species? How are they quantified?

3. Figure 3c – shows the incorporation of iron atoms into the nitrogenated carbon structure leading to more ordered graphite sheets. How can we tell this is the right sequence of events? What if the ordered carbon is formed first by the catalytic action of iron (known to pyrolyze hydrocarbons to make graphites, carbon nanotubes, etc.) and then the iron single atoms become anchored by the N atoms?

4. Why are the XRD reflections lacking at 1000C when metallic Fe nanoparticles are formed?

5. What is NC? It is just mentioned at the start of the discussion without a clear explanation as to what it is.

6. In the introduction, the manuscript states that Fe site loadings demonstrated so far remain at a very low level, < 3 wt%. However, there have been demonstrations, such as Nature Communications volume 10, Article number: 1278 (2019), that go above such loading. There are probably more articles that demonstrate Fe loading > 3 wt% than any other element. The authors should revise this statement or clarify what their original intentions are.

Responses to Reviewers' Comments

We sincerely appreciate the reviewers for the expert and valued comments and constructive suggestions which greatly help us to improve the quality of this submission (NCOMMS-23-59721A). In the following, we carefully respond the reviewers' comments point by point (texts in blue), and describe the corresponding revisions made on the manuscript and Supplementary Information (orange font). The figures in this Response Letter are named as Figure R**.

Reviewer #1 (Remarks to the Author):

In the manuscript, the authors provided a visualized process for the conversion of the Fe2O3 particles into FeN_x single atom site. The manuscript was well organized, and the research methodology could be helpful in studying similar catalysts. However, significant concerns of this work are the novelty and potential impact. Recently, similar results of using Fe2O3 as a precursor were discovered and reported to synthesize single Fe atom catalysts. The dynamic conversion of Fe2O3 nanoparticles to single Fe sites in catalysts was also elucidated. Such correlations between catalytic activity and heating temperatures were also studied in the literature. Furthermore, the presenting catalyst performance in both aqueous electrolyte and fuel cells is inferior to those recently reported. Therefore, this manuscript is not recommended for publication in the journal. Meanwhile, more detailed technical comments are listed below:

Response:

We are thankful to Reviewer #1 for taking the time to evaluate our manuscript. We deeply thank the reviewer for the affirmation of our manuscripts, believing that our manuscripts are well organized, and the research methodology could be helpful in studying similar catalysts. The similar results mentioned by the reviewer may come from the recent work published by Wu's Group on *Nat Energy 7, 652-663 (2022)*. In fact, we have cited and discussed the recent articles on the formation mechanism of M/N/C active sites in the introduction of our manuscript, including this one. The transition behavior from FeO_x to FeN_x was mentioned by Wu et al. [*Nat Energy 7, 652-663 (2022)*] and Jia et al. [*J Am Chem Soc 142, 1417-1423 (2019)*]. These findings, however, cannot explain the origin of FeO_x. They only indicate that Fe oxides can be transformed into $Fe–N_4$ sites at high temperatures. The details and precautions of the conversion of FeO_x to FeN_x are also not mentioned.

In this manuscript, we aim to highlight the primary novelty and focus of our research, which lies in the development of an array of in-situ characterization techniques. These methods enable a systematic investigation into the evolution of Fe species and the tracking of $Fe–N_4$ site formation. We elucidate the origin of FeO_x, detail the transformation process from FeO_x to FeN_x, and ultimately furnish insights for the rational design of Fe/N/C catalysts. To integrate multiple in-situ

characterizations effectively, we have employed the most straightforward synthesis approach. This method may not yield full conversion of FeO_x to FeN_x active sites, potentially leading to suboptimal performance of the Fe/N/C catalysts. Nevertheless, our findings theoretically underpin the high-performance Fe/N/C catalysts recently documented, such as the contactless vapor deposition [*Nat Mater 20, 1385-1391 (2021)*] and the utilization of 5 nm Fe_2O_3 as the iron precursor [*Nat Energy 7, 652-663 (2022)*]. These constitute the core novelty and focus of our study.

In fact, this work began in 2021 and has been researched for three years. During this period, we also rationally designed two types of high-performance catalysts through our proposed four-point suggestions:

1) We designed a bottom-up "$FeCl_2$ gas-phase capture" device that achieved contactless gas-phase deposition, effectively avoiding the formation of FeO_x during pyrolysis, thus enhancing the site density to 5.67×10^{19} sites g^{-1}. This catalyst achieved a peak power density of 1.53 W cm^{-2} (absolute 2.5 bar) and a current density of 46 mA cm^{-2}@$0.9V_{iR\text{-}free}$, proving that our proposed strategy is genuinely effective [Energy Environ. Sci., 2022, 15, 3033-3040].

2) More recently, we have introduced acidic gases during the pyrolysis process to react with the formed FeO_x, thereby achieving the goal of refining the size of FeO_x nanoparticles and converting more sites, with a site density that can reach 6.31×10^{19} sites g^{-1}. This catalyst can achieve a peak power density of 1.86 W cm^{-2} (absolute 2.5 bar) and a current density of 54 mA cm^{-2}@$0.9V_{iR\text{-}free}$ (absolute 1.5 bar) (**Figure Rx**), similarly demonstrating the effectiveness of our proposed strategy. This work has been submitted to *Nature Communications* and is currently in Revision [Manuscript ID: NCOMMS-23-61683-T].

In summary, we believe that our work has certain theoretical guiding significance for the design of Fe/N/C materials and is conducive to advancing the development of this field.

1. The activity and stability of PGM-free ORR catalysts have been continuously improved recently. The claim of "We recently reported a Fe/N/C catalyst with a performance of 710 mW cm^{-2} in H_2/air PEMFC, which is the highest value yet." (Line 49) is not fair. Especially, when the test conditions are different, the peak power density will change significantly. A neutral statement should be made here. We encourage the authors to compare the fuel cell performance at practical operating parameters, such as current densities at 0.67, 0.7 or 0.8 V in H_2/air at ambient pressure.

Response 1:

We appreciate the insightful comment. Indeed, varying test conditions do influence the peak power density. Our initial statement was somewhat lacking in impartiality. To address this, we have conducted a comparative analysis of the current density at 0.8 V in H_2-air PEMFCs, as detailed in **Table R1**. Our analysis reveals that the performance of previously reported Fe/N/C catalyst is surpassed only by two recent catalysts, thus outperforming the majority of M/N/C catalysts reported to date. To reflect the experimental outcomes more accurately, we have revised the relevant sections of the manuscript.

"We recently reported a Fe/N/C catalyst with a performance of 710 mW cm^{-2} and 120.8 mA cm^{-2} at 0.8 V in H_2-air PEMFCs[8], which is more than most of the reported M/N/C catalysts."

Furthermore, we evaluated the air performance of the 0.015Fe-NC-900 catalyst following the standard test protocol for non-precious metal oxygen reduction electrocatalysts as recommended by Piotr Zelenay [*Nat. Catal., 5, 455-462 (2022)*]. The results, illustrated in **Figure R1a-b**, show that the catalyst achieves a peak power density of 0.45 W cm^{-2} (at an absolute pressure of 1.5 bar) and a current density of 64.2 mA cm^{-2} at 0.8 V, positioning it at a mid-level among high-performance Fe/N/C catalysts. Nonetheless, it is important to clarify that the focus of our work is to elucidate the evolution of active sites during the pyrolysis of Fe/N/C materials rather than on performance optimization.

Additionally, following the principles of rational design from our work, we have synthesized a Fe-NC^{BrCl} catalyst. This design utilizes NH_4Cl and NH_4Br which decompose to produce acidic gases, thereby inhibiting FeO_x growth during pyrolysis and enhancing the conversion of Fe-N_4 sites. The details of this process are addressed in our response to *Reviewer #2, Answer iv)*. Employing this strategy, we increased the active site density from 2.66 × 10^{19} sites g^{-1} to 6.31 × 10^{19} sites g^{-1}, resulting in a hydrogen-air fuel cell peak power of 0.88 W cm^{-2} at an absolute pressure of 2.5 bar, with a current density of 259 mA cm^{-2} at 0.8 V (**Figure R1c-d**). These experimental findings substantiate the viability of our proposed methodology.

Table R1. Comparison of H_2–air PEMFCs activity of Fe_g-NC/Phen in our previous work (Environ. Sci., 2022, 15, 3033-3040) with other reported M-N-C catalysts.

**Catalyst**	**P_{max} / W cm^{-2}**	***J*@0.8V / mA cm^{-2}**	**Abs. Pressure / bar**	**Ref.**
**0.015Fe-NC-900**	**0.45**	**64.2**	**1.5**	**This work**
**Fe_g-NC/Phen**	**0.71**	**120.8**	**1.5**	**Our previous work, Energy Environ. Sci., 2022, 15, 3033-3040**
**Fe-NC^{BrCl}**	**0.88**	**259**	**2.5**	**Our other work**
Fe-N-C-FG	~0.72	191.0	1.5	*Nat. Catal.*, 2023, 6, 1215-1227
Fe-AC	~0.60	151	1.5	*Nat. Energy*, 2022, 7, 652-663
FeNC-CVD-750	~0.37	~40	1.5	*Nat. Mater.*, 2021, 20, 1385-1391
Fe-MOF catalyst	0.61	~50	1.0	*ACS Appl. Mater. Interfaces.* 2020, 12, 2216
SA-FeNx-ZIF8-PCM	0.60	~100*	1.9	*Nano Energy* 2021, 83, 105734
NPMC (CA#1)	0.57	~80*	1..74	*Sci. Adv.* 2018, 4, 7180
Mn−N−C−S	~0.50	~80*	2	*ACS Nano* 2021, 15, 6886
(Fe,Co)/N-C	0.51	54*	2	*J. Am. Chem. Soc.* 2017, 139, 17281
Fe/N/C(4mIm)-OAc	0.47	~40*	1	*Adv. Funct. Mater.* 2021, 31, 2009645
TPI@Z8(SiO_2)-650-C	0.42	105	1	*Nat. Catal.* 2019, 2, 259
(CM+PANI)-Fe-C	0.42	75	1	*Science* 2017, 357, 479
Fe-N-C-Phen-PANI	0.38	85*	1.88	*Adv. Mater.* 2017, 29, 1604456
FePhen@MOF-$ArNH_3$	0.38	50	2	*Nat. Commun.* 2015, 6, 7343
Co(mIm)-NC(1.0)	~0.32	~30*	1	*Nat. Catal.* 2020, 3, 1044
Fe-MOF-700/1000	0.30	~30*	1.5	*J. Mater. Chem. A* 2014, 2, 12270

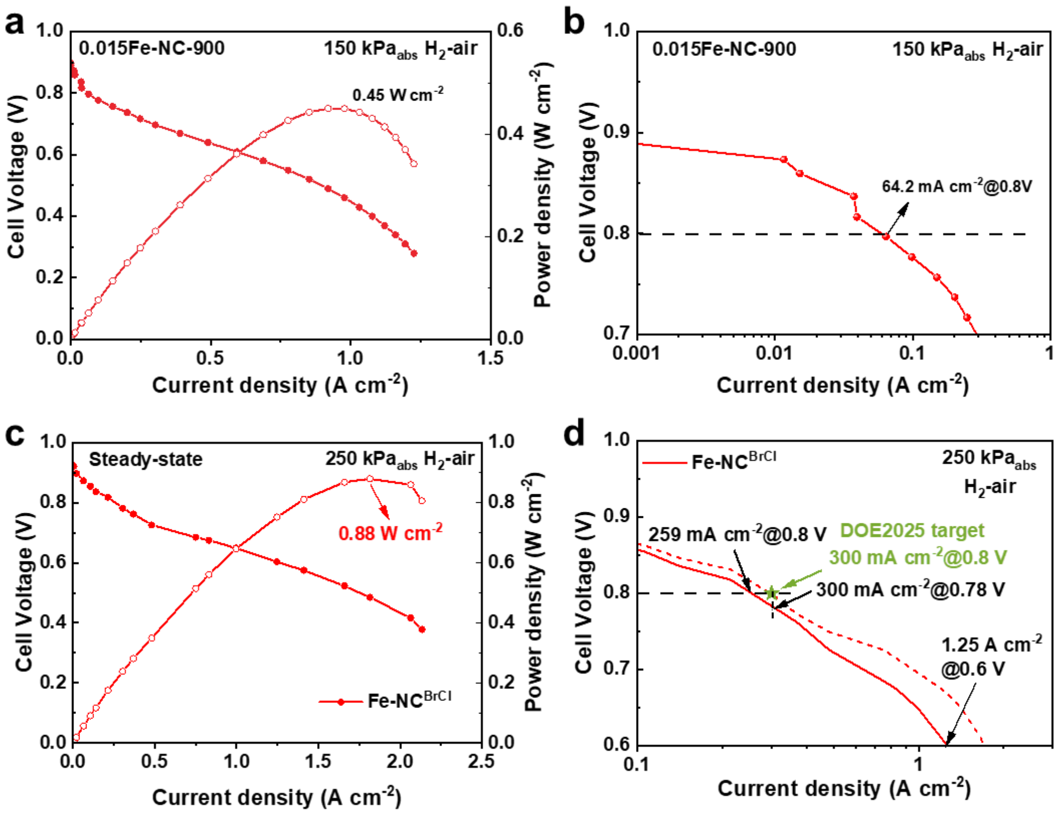

Figure R1. (a) H_2-air fuel cell *i*-*V* polarization and powder density plots of 0.015Fe-NC-900 record under absolute 1.5 bar of air pressure; (b) Current density at 0.8 V; (c) H_2-air fuel cell *i*-*V* polarization and powder density plots of Fe-NC^{BrCl} record under absolute 2.5 bar of air pressure; (d) Current density at 0.8 V. Test condition: H_2-air polarization curves, acquired from OCV to 0.70 V in 25 mV steps and 0.70 V to 0.25 V in 50 mV steps, with a hold time of 45 s per point. 0.3 L H_2 min^{-1} and 0.4 L air min^{-1} feed, 100% relative humidity (RH), 80 °C, cathode loading, 3.5 mg cm^{-2}.

Above discussions were also supplemented in the Supplementary Information (**Supplementary Fig. 46, Table 13, Note 7**).

2. The explanation of the migration of the FeO_x nanoparticles in the N-doped carbon is speculative and subjected to debate. The concept of "The surface carbothermic reaction produced an energy release that aided in the conversion process" was proposed as the driving force for the migration of the Fe-based nanoparticles. The explanation spread across the manuscript (e.g., Line 153, Line 169, Line 226). However, a carbothermic reaction is an endothermic reaction. How could this process supply energy to other processes and kick them off? The hypothesis lacks support from the model or simulation. It is improper to claim a counterintuitive concept without theoretical evidence to support the major findings in this work.

Response 2:

Thanks for the value comment. We refer to the relevant information, the carbon thermal reaction is an endothermic process. For example, the reaction of carbon and iron oxide to produce carbon dioxide and iron can be expressed by the following equation:

$$Fe_2O_3\ (s) + 1.5C\ (s) \rightarrow 2Fe\ (s) + 1.5CO_2\ (g)$$

The standard enthalpy change (ΔH^{θ}) of the reaction can be calculated according to the standard molar enthalpy of formation of the substance. The ΔH^{θ} of this reaction is usually endothermic, with a value of about +234.1 kJ mol^{-1}.

We are grateful that the reviewer can point out this error. We analyzed the source of driving force of nanoparticle movement by consulting the literature. It may be due to the following two reasons:

1) The nano-size effect reduces the melting point of nanoparticles.

The melting point of nanoparticles is significantly influenced by their size due to surface effects and quantum size effects. As the particle size decreases, the increased surface-to-volume ratio results in a higher surface energy and a reduced coordination number for surface atoms, making them less stable and easier to melt at lower temperatures [*Prog. Mater. Sci., 2007, 52, 1175-1262*; *Nature, 1977, 269, 481-483*].

With the development of characterization techniques, researchers have used *in-situ* transmission electron microscopy to capture the phenomenon of nanoparticle migration in carbon. For example, Zhiping Zheng et al. reveals the etching of the original imidazolate support by free-moving Co nanoparticles at 850 °C by *in-situ* environmental transmission electron microscope (ETEM) (**Figure R2**). These nanosized Co nanoparticles melt at 850 °C, lower than the melting point of bulk form Co (1495 °C) due to the size-dependent melting of small particles that the melting point of metal nanoparticles significantly decreases with decreasing particle size.

The melting point of iron oxide is above 1500 ºC. When the size of nanoparticles is as low as 10 nm, it may also melt, so it will migrate in carbon.

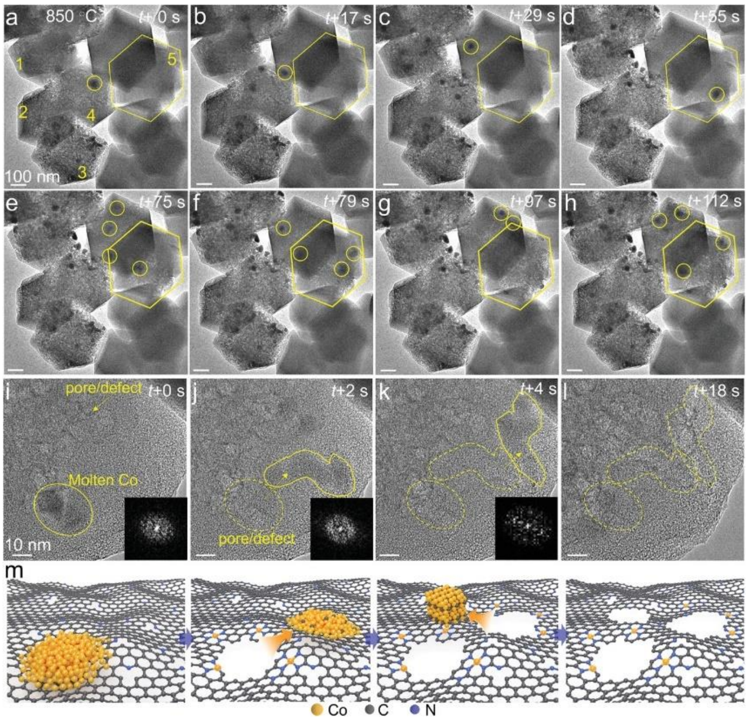

Figure R2. *In-situ* ETEM showing the molten Co etching ZIF to produce porous structure CN_x at 850 °C.

2) Gas drives the movement of nanoparticles

Mechanistically, when nanoparticles are exposed to carbon substrate or environmental conditions (such as light, temperature changes, or the presence of certain chemicals), carbothermic reaction on their surfaces generate gases. These gas molecules create a higher local pressure on one side of the nanoparticle, while the opposite side experiences lower pressure. According to the principles of physics related to forces and pressure, this pressure gradient exerts a force, driving the nanoparticle from the high-pressure area towards the low-pressure area.

Furthermore, the efficiency and direction of nanoparticles' migration on carbon surfaces are influenced not only by the kinetics of the catalytic reactions but also by the interactions between the nanoparticles and the carbon substrates. These interactions include but are not limited to van der Waals forces, electromagnetic interactions, and the chemical and physical properties of the carbon surfaces (such as roughness and surface functional groups).

Nanoparticle propulsion via gas generation has been documented in micromotor research. For instance, **Figure R3a** illustrates a micromotor designed by Jianguo Guang and colleagues, which is propelled by hydrogen bubbles produced from the reaction of magnesium with water [*Angew. Chem. Int. Ed., 2013, 125, 7349-7353*]. Similarly, as shown in **Figure R3b**, Chad A. Mirkin and his team have developed Au/Pt nanorotors (NRs) capable of rotation in a hydrogen peroxide solution [*J. Am. Chem. Soc. 2007, 129, 14870-14871*]. They determined that the motion's driving force in this catalysis-based nanomotor system stems from the dynamic breakdown of H_2O_2, releasing O_2. Consequently, it is reasonable to postulate that the gas produced by the carbothermal reaction on the FeO_x surface could likewise facilitate the transport of nanoparticles.

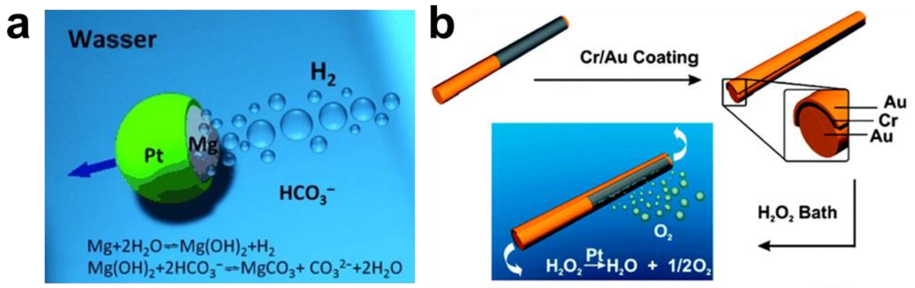

Figure R3. (a) The micromotor is powered by hydrogen bubbles produced by the reaction of magnesium with water [*Angew. Chem. Int. Ed., 2013, 125, 7349-7353*]. (b) Schematic diagram of the synthesis of Au/Pt NR and its motion in a H_2O_2 bath [*J. Am. Chem. Soc. 2007, 129, 14870-14871*].

By integrating two influential factors, we posit that the propulsion of nanoparticles is attributed to the size effect-induced reduction in melting point, and concurrently, the gas produced from the surface carbothermal reaction contributes to the driving force. We revised the migration driving force of nanoparticles in the manuscript and cited relevant literature. Thanks again for the problems pointed out by the reviewer.

"The migration of nanoparticles is primarily driven by the size-dependent reduction in their melting point[29] and the gas production from carbothermal reactions occurring on their surfaces[30, 31]."

3. Regarding to the suggestions for the rational design of Fe/N/C catalysts, NH_4Cl and NH_4Br treatment (Line 289) have been reported in the previous literature and served a postal treatment to the raw catalyst. The suggestion does not match with the findings reported in this paper and undermines the significance of the paper.

Response 3:

Thanks for the value comment. As the Reviewer said, NH_4Cl treatment has been reported in the literature, NH_4Br treatment related reports are less, but the two treatment principles are similar. The following may be the two related work mentioned by the reviewer:

1) Xinliang Feng *et al.* firstly reported a low-temperature NH_4Cl-treatment to etch out graphene-encapsulated nanoparticles from metal-N-C catalysts without destruction of co-existing atomically dispersed metal-N_x sites [*Angew. Chem. Int. Ed., 2020, 59, 1627-1633*]. As shown in **Figure R4a**, Feng *et al.* suggested that under such low-temperature condition (350 ^oC), the metal atoms of encapsulated M-NPs became unstable and reactive and could diffuse slowly across the graphitic shells through the pores and defects and react with outer NH_4Cl to form water-soluble ammonium metallic chloride compounds. In other words, NH_4Cl can redisperse the nanoparticles into smaller clusters or even single atoms at 350 ^oC.

2) Gang Wu et al. reported a Fe-N-C catalyst with highly dispersed Fe species (Fe-AC) [*Nat. Energy, 2022, 7, 652-663*]. As shown in **Figure R4b**, Fe_2O_3 nanoparticles with a size of ~5 nm were added during the growth of ZIF-8 to form Fe_2O_3@ZIF-8 composite precursor. Then the composite precursors were subjected to a heating treatment at 800 °C for 30 min under flowing Ar gas to obtain a sample denoted as Fe–N–C-800. Finally, the Fe–N–C-800 were ground with NH_4Cl salts thoroughly, followed by a heating treatment at 1,100 °C under Ar flow for 1 h to obtain the Fe-AC catalyst. Wu et al. suggested that NH_4Cl decomposes to NH_3 and HCl gas at high temperatures, which produce substantial internal stress and etch the carbon, thus creating a multitude of micropores and carbon defects [*J. Phys. Chem. C, 2007, 111, 19033–19042*], similar to previously reported Fe–N–C catalysts with NH_3 gas treatments [*Science, 2009, 324, 71–74*]. The HCl probably further dissociates at high temperatures to form H_2 and Cl_2 gases, and according to Gewirth et al. [*Nat. Commun., 2016, 7, 12582*], the Cl_2 gas reacts with residual Fe aggregates in catalysts and facilitates the formation of atomically dispersed Fe sites.

Both works were completed by post-treating the original Fe-N-C catalyst with NH_4Cl. The ORR performance was enhanced to various extents. We propose the treatment with NH_4Cl and NH_4Br during the synthesis of Fe-N-C rather than the secondary treatment of Fe-N-C materials, although the treatment procedures differ from the two works. However, we believe that these works can help substantiate our suggestion. Firstly, Feng's work confirms that NH_4Cl can indeed decompose nanoparticles at low treatment temperatures, which aligns with our desired outcome. The difference is that we consider NH_4Cl to decompose at low temperatures to form the acidic gas HCl, and use HCl to react with the formed FeO_x, decomposing it. Secondly, Wu's work used 5 nm Fe_2O_3 as the iron source to achieve high dispersion from the first step of synthesizing Fe-N-C, which is consistent with our work. When the size of FeO_x nanoparticles is less than 7 nm, they are prone to complete decomposition at high temperatures. Once nanoparticles exceed 10 nm, they tend to agglomerate and even reduce to metallic Fe. Finally, Wu et al. also noted in their work that "HCl may further decompose into H_2 and Cl_2 gases at high temperatures, and the Cl_2 gas reacts with the residual Fe

aggregates in the catalyst, promoting the formation of atomically dispersed Fe sites." This also demonstrates that the acidic gas HCl can indeed assist in the formation of atomically dispersed Fe species.

Incidentally, we have also verified the validity of our proposed methodologies through experimental trials. In the pyrolysis of Fe-N-C, we employed NH_4Cl and NH_4Br to successfully decompose FeO_x. This approach enhanced the dispersion of Fe, facilitated the formation of additional Fe-N_4 active sites, and consequently, substantially elevated the performance of the fuel cells. For details on the specific procedure, please refer to the response to ***Reviewer #2, Answer iv)***.

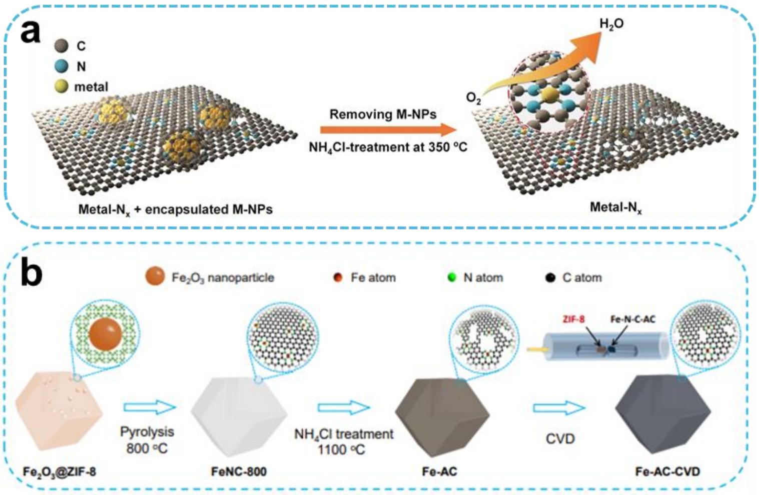

Figure R4. (a) Schematic illustration of the removal of graphene-encapsulated M-NPs from M-N-C ORR catalysts by a low-temperature NH_4Cl-treatment process [*Angew. Chem. Int. Ed., 2020, 59, 1627-1633*]. (b) Schematic synthesis of the highly stable Fe-N-C catalyst via a two-stage approach, consisting of (i) the Fe_2O_3@ZIF-8 derived Fe-N-C catalyst was treated with NH_4Cl salts at high temperatures, denoted as Fe-AC and (ii) a CVD process using ZIF-8 as the carbon and nitrogen sources to deposit a thin layer of N-doped carbon onto the NH_4Cl treated Fe-N-C catalysts [*Nat. Energy, 2022, 7, 652-663*].

In order to improve the rationality of our proposal, we cited the corresponding literature, and added some explanation in the Manuscript.

"NH_4Cl has been shown to remove nanoparticles at high temperatures, contributing to the formation of atomically dispersed Fe sites[23, 34]. Besides, FeO_x can be produced and their development inhibited or even stopped by reacting with acidic gasses."

4. Some typos in the paper. In Line 30, does the metrics mean "matrix"? Line 123, a comma before "at 350 °C" could be deleted.

Response 4:

We sincerely thank the reviewers for their careful review. We have carefully examined the full manuscript. The corresponding typos have been corrected in the revised manuscript.

5. In Supplementary Fig.1, please specify what species refers to the domain peak at 35.1 from 600Co to 900Co.

Response 5:

Thank you for the valuable comment. The peak at 35.1° observed in Supplementary Figure 1 corresponds to the feature identified in Figure 1a of the manuscript (lines 109-117). As depicted in Figure R5, the hexagonal Fe_2O_3 structure (R-3c) becomes evident at temperatures reaching 350 °C. With the temperature increase to 500 °C, this hexagonal Fe_2O_3 structure initiates a transition into a cubic Fe_3O_4 structure (Fd-3m), as evidenced by the disappearance of the hexagonal $(104)_R$ peak at 33.1° and the shift of the $(110)_R$ peak at 35.6° towards the cubic $(311)_R$ peak at 35.4° in the lower angle regime. Upon further elevating the thermal activation temperature to 800 °C, the cubic Fe_3O_4 phase undergoes a transformation into the cubic FeO phase (Fm-3m), with the primary peak shifting to 35.8°.

To aid readers in comprehension, we have indicated in the manuscript that "original data and the characteristic peak have been identified in Supplementary Fig. 1.".

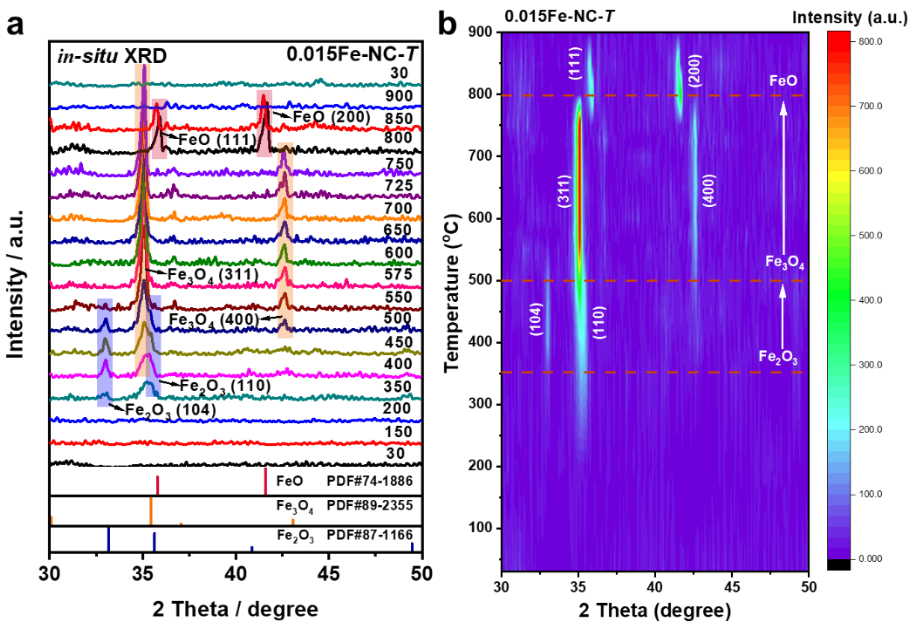

Figure R5. (a) The XRD patterns and the corresponding contour maps of 0.015Fe-NC-*T* during *in-situ* heating XRD measurement.

6. In this work, the only possible FeN4 site observed by the TEM is the images in Supplementary Fig.22, which are shown as bright dots. However, the bright spots are not the strong evidence of the FeN_4 site. The EELS spectroscopy must be employed to demonstrate the universal existence of the FeN_4 site.

Response 6:

Thanks for the value comment. We supplemented the EELS spectrum, as shown in **Figure R6**. The Fe and N are uniformly distributed on the carbon support (**Figure R6a**). EELS spectra simultaneously detected Fe and N (**Figure R6b**), suggesting the formation of Fe-N_x sites.

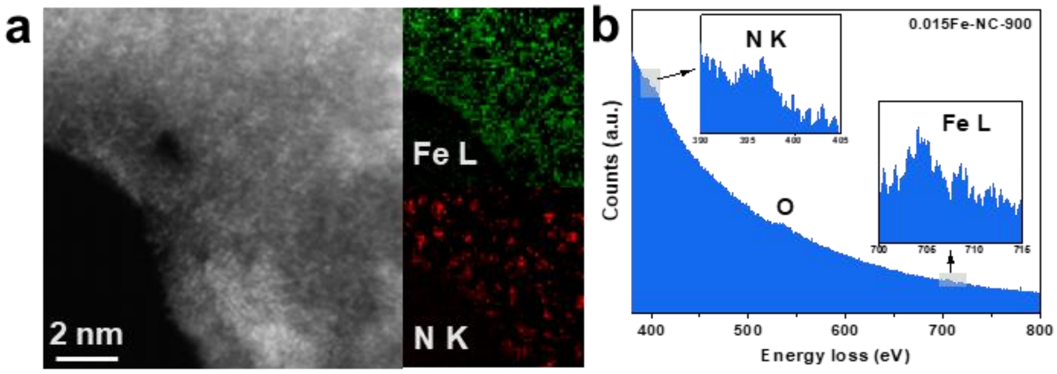

Figure R6. (a) HAADF-STEM image and EDS elemental maps; (b) corresponding EELS spectra of the 0.015Fe-NC-900.

In order to improve the quality of our manuscript, we added the EELS spectrum to **Supplementary Fig.25** and added descriptive words to the manuscript.

"Furthermore, Fe single atoms embedded in the ordered carbon layer were visible in the aberration corrected high-angle annular dark field scanning transmission electron microscopy (AC-HAADF-STEM) image of 0.015Fe-NC-900, and electron energy-loss spectra (EELS) simultaneously detected Fe and N, suggesting the formation of Fe-N_x sites (Supplementary Fig. 25)"

7. As the author mentioned in Figure 5b, only 44% of Fe contents are converted into FeN4 site. The other Fe species are Fe or FeOx. However, the Fe and FeOx are unstable during the Fuel cell operation in the acid media. The durability result of the fuel cell based on Fe-N-C catalysts is necessary to illustrate the benefits or negatives of the existence of FeOx in the catalysts for ORR reaction.

Response 7:

We appreciate the insightful feedback. To ascertain the durability of Fe-N-C materials embedded with Fe and FeO_x nanoparticles in fuel cell applications, we have expanded upon the stability testing for the 0.015Fe-NC-900 catalyst. The findings and the corresponding test parameters are depicted in **Figure R7** and its legend. A rapid degradation was observed in the 0.015Fe-NC-900 cathode, with a 67% loss after 23 h. This aligns with the instability commonly reported for Fe-N-C catalysts in existing literature, suggesting that the inclusion of Fe and FeO_x nanoparticles does not enhance the durability of Fe-N-C catalysts in fuel cells. Conversely, under fuel cell operational conditions, FeO_x may dissolve and release cationic Fe^{3+}. This dissolution can lead to Fe^{3+} deposition on the anode, which undermines fuel cell performance. Additionally, the Fenton reaction may occur in the presence of the by-product H_2O_2, generating reactive oxygen species that accelerate carbon corrosion and degrade the proton exchange membrane [*Chem. Rev. 2023, 123, 9265−9326*; *Chem. Soc. Rev., 2020, 49, 3484--3524*].

For ease of understanding, we have included the additional experimental data in the Supplementary Information (**Supplementary Fig. 46, Note 7**).

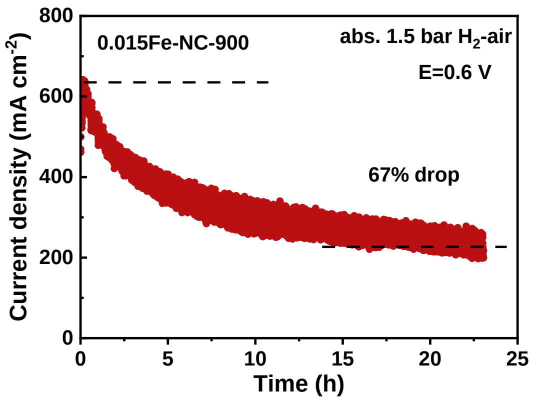

Figure R7. The fuel cell durability of 0.015Fe-NC-900 catalyst under H2-air conditions at a constant potential of 0.6 V (1.5 bar absolute air pressure and flow rates of air 0.3 L min^{-1} and H_2 0.3 L min^{-1}. Anodes: Pt/C, 0.40 mg_{Pt} cm^{-2}, cathodes: Fe-N-C, 3.5 mg cm^{-2}, 100% RH, and 1.5 bar absolute H_2 pressure. Membrane: Nafion 211. Temperature: 80 °C. MEA area: 4.41 cm^2.

Reviewer #2 (Remarks to the Author):

This manuscript delves into the in-situ observation and mechanistic exploration of the structural transformation occurring during the pyrolysis of Fe/N/C electrocatalysts. Such insights are crucial for advancing the efficacy of oxygen reduction reaction catalysts. With the potential to supplant platinum-based alternatives, research on Fe/N/C catalysts is important. The integration of machine learning with theoretical computations in this work sheds light on the development of Fe species and the genesis of Fe–N_4 sites. This methodological innovation bestows a fresh perspective and a novel toolset for probing Fe/N/C catalysts, thereby enriching the depth and breadth of the research. The authors articulate a strategy to modulate the size of iron oxide particles during pyrolysis, aiming to augment the conversion rate to Fe–N_4 sites. This inventive approach holds guiding relevance for fabricating high-performance Fe/N/C catalysts.

The manuscript boasts a coherent structure, sound experimental design, meticulous data analysis, and comprehensive discussion, which collectively buttress conclusions of the manuscript. It is recommended for publication after minor revisions.

Response:

We are thankful to Reviewer #2 for their positive evaluation of our manuscript and thoughtful suggestions that have helped us improve our work.

i) The authors should address the changes in specific surface area, pore structure, and carbon defects through the pyrolysis process.

Response i:

Thanks for the value comment. According to the suggestions of Reviewer, we supplemented the following experiments:

1) The evolution of specific surface area and pore structure during pyrolysis

We investigated the evolution of the specific surface area and pore structure of 0.015Fe-NC-*T* materials during pyrolysis using nitrogen adsorption-desorption isotherms. **Figure R8** presents the statistical analysis of the specific surface area and pore size distribution derived from N_2 isothermal adsorption-desorption tests. Compared with the control sample NC, the specific surface area of 0.015Fe-NC-RT decreased from 811.44 $m^2 g^{-1}$ to 487.95 $m^2 g^{-1}$, as shown in **Figure R8a** and **Table R2**. At ambient temperature, there is a reduction in the volume of micropores in the precursor, while the mesoporous volume remains unchanged (**Figure R8b** and **c**). This suggests that $FeCl_2 \cdot 4H_2O$ may occupy the micropores, undergoing oxidative hydrolysis to yield $Fe(OH)_3$. Upon heating beyond 400 °C, dehydration of $Fe(OH)_3$ leads to the formation of FeO_x nanoparticles, which frees the micropores, thereby restoring the pore volume to levels comparable with the NC substrate. As the temperature further increases to 800 °C, the nanoparticles diminish in size, liberating additional micropores and mesopores and consequently enhancing the specific surface area. Above 900 °C, the majority of nanoparticles decompose into isolated atomic sites and clusters. Concurrently, the etching effect of mobile nanoparticles and carbon consumption through carbothermal reactions lead to an augmentation in the proportion of micropores and mesopores within carbon supports, with the specific surface area increasing to 949.60 $m^2 g^{-1}$.

In conclusion, the changes in the specific surface area and pore size distribution of the material as a function of pyrolysis temperature align with the findings reported in our manuscript.

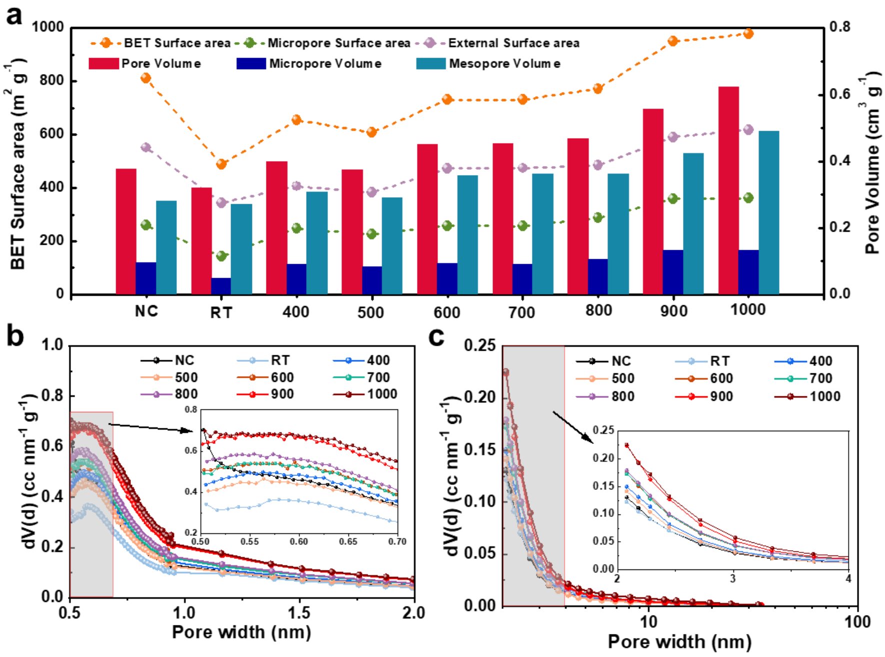

Figure R8. Structure evolution of 0.015Fe-NC-*T* carbon hosts during thermal activation at different temperatures. (a) A comprehensive comparison of BET surface areas and porosity; (b) Micropore size and (c) mesopore size distributions.

Table R2. Porosity of 0.015Fe-NC-*T* samples.

Samples	S_{total} / $m^2\ g^{-1}$	S_{Micro} / $m^2\ g^{-1}$	$S_{External}$ / $m^2\ g^{-1}$	Pore Volume / $cm^3\ g^{-1}$	Micropore volume / $cm^3\ g^{-1}$
NC	811.44	260.13	551.31	0.378	0.097
0.015Fe-NC-RT	487.95	143.42	344.53	0.320	0.048
0.015Fe-NC-400	655.02	248.41	406.60	0.399	0.091
0.015Fe-NC-500	607.77	225.41	382.37	0.374	0.082
0.015Fe-NC-600	731.49	258.06	473.43	0.451	0.093
0.015Fe-NC-700	731.05	256.63	474.42	0.454	0.092
0.015Fe-NC-800	772.76	287.86	484.91	0.468	0.106
0.015Fe-NC-900	949.60	358.79	590.82	0.557	0.133
0.015Fe-NC-1000	979.17	360.90	618.27	0.623	0.133

2) The evolution of carbon defects during pyrolysis

The enhancement of mesoporosity and microporosity typically results in an increased occurrence of defects on the carbon support. Raman spectroscopy serves as a reliable technique for examining the structural attributes of carbon-based materials. In this study, we employed Raman spectroscopy to investigate the evolution of carbon defects during the pyrolysis of 0.015Fe-NC-T materials. The experimental findings are depicted in **Figure R9**. Peak deconvolution of the Raman spectra was conducted, where the I peak signifies the sp^2 hybridized domains of graphene, the D peak represents carbon defects, the D’’ peaks indicate distortions due to C5 rings or heteroatoms, and the G peak corresponds to sp^2 hybridized graphene [*Angew. Chem. Int. Ed., 2021, 60, 9516-9526*]. Typically, the ratio of the D peak area to the G peak area serves as an indicator of the degree of carbon defectiveness. A higher ratio suggests a greater degree of defectiveness. The deconvoluted spectra and the aggregated data are presented in **Figures R10** and **Table R3**, respectively. It is observed that the degree of carbon defects increases progressively with rising pyrolysis temperatures, attributable to the formation of an increased number of micropores and mesopores, corroborating with the results from BET analysis.

In conclusion, with escalating pyrolysis temperatures, nanoparticles undergo a gradual transformation from formation to dissolution, concomitantly enhancing the specific surface area of 0.015Fe-NC-*T* material. Furthermore, the etching effect of mobile nanoparticles and carbon consumption through carbothermal reactions lead to an augmentation in the proportion of micropores and mesopores within carbon supports, thereby amplifying the degree of carbon defects.

In order to enhance the quality of our manuscripts, we have included the experimental data in the Supplementary Information (**Supplementary Fig. 43-45, Table 11-12, Note 6**).

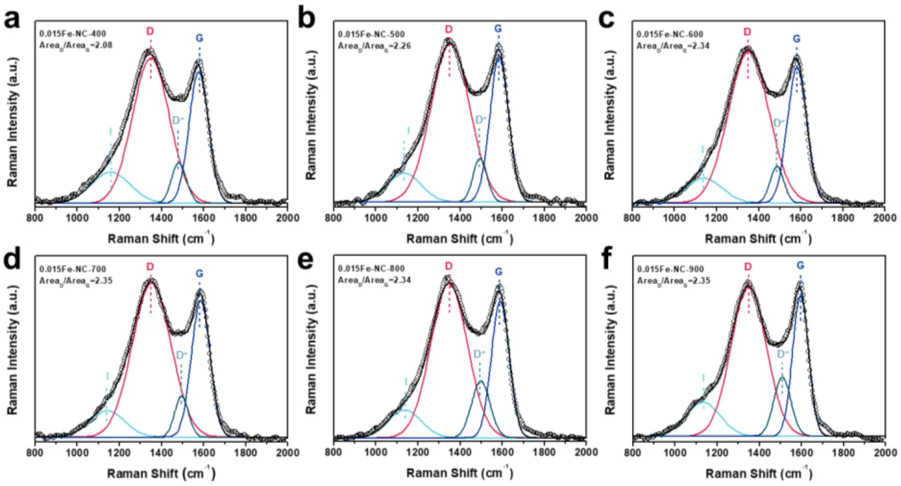

Figure R9. Raman spectra of 0.015Fe-NC-T samples obtained after thermal activation at different temperatures.

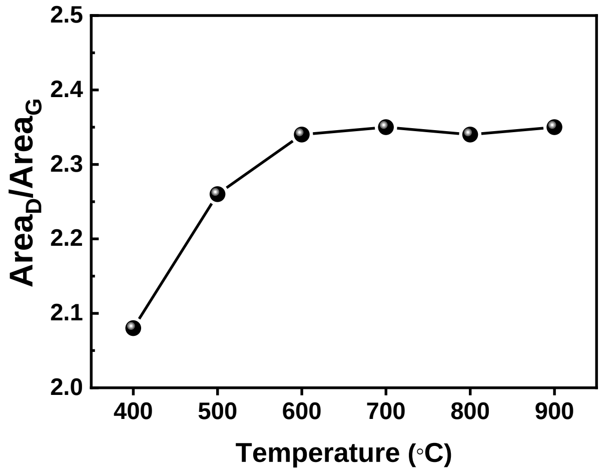

Figure R10. $Area_D/Area_G$ of 0.015Fe-NC-*T* samples obtained after thermal activation at different temperatures.

Table R3. Raman fitting results of 0.015Fe-NC-T samples obtained after thermal activation at different temperatures.

Samples	I peak%	D peak%	D" peak%	G peak%	$Area_D/Area_G$
0.015Fe-NC-400	12.7%	54.9%	6.2%	26.3%	2.08
0.015Fe-NC-500	9.3%	58.8%	5.8%	26.0%	2.26
0.015Fe-NC-600	9.0%	60.3%	4.9%	25.7%	2.35
0.015Fe-NC-700	9.3%	59.7%	5.6%	25.4%	2.35
0.015Fe-NC-800	9.2%	56.3%	10.4%	24.1%	2.34
0.015Fe-NC-900	12.0%	54.4%	10.5%	23.1%	2.35

ii) The influence of nitrogen in the carbon matrix warrants consideration; for instance, is it feasible to synthesize single-atom catalysts with carbon supports devoid of nitrogen doping?

Response ii:

Thanks for the value comment. To further analyze the effect of nitrogen element, we used porous carbon (PC) without nitrogen (S_{BET} = ~1000 m^2 g^{-1}) containing the same amount of Fe element. The $FeCl_2{\cdot}4H_2O$ also hydrolyzes on porous carbon and forms FeO_x during pyrolysis. However, due to the lack of anchoring effect of nitrogen on Fe atoms, FeO_x is completely reduced to Fe and serious agglomeration occurs (**Figure R11**). Therefore, The N sites help to anchor the single Fe atom produced by the decomposition of FeO_x to form FeN_x sites.

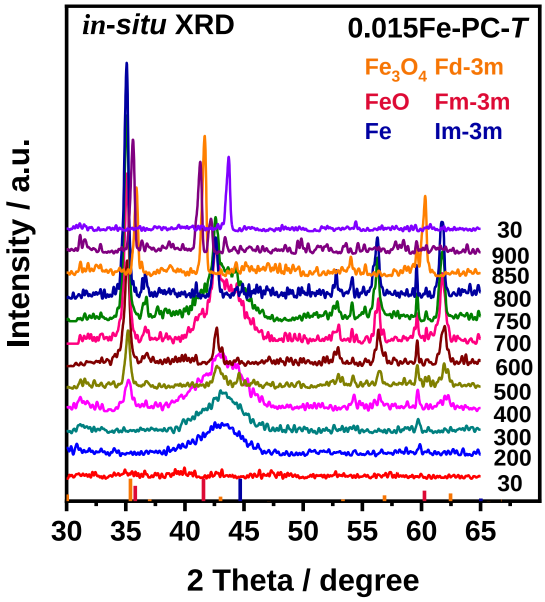

Figure R11. The *in-situ* heating XRD experiment of 0.015Fe-PC-*T*.

iii) The authors need to provide 57Fe Mössbauer spectroscopy characterization to demonstrate the conversion trajectory of iron oxide nanoparticles.

Response iii:

We are deeply appreciative the valuable suggestion of reviewer. We supplemented the ^{57}Fe Mössbauer spectroscopy characterization at 23 K, with the results presented in **Figure R12**.

The designation of D1 and D2 is based on the work of Frédéric Jaouen *et al.* [*Nat. Catal., 2021, 4, 10-19*]. From *in-situ* ^{57}Fe Mössbauer spectroscopy in inert gas coupled to calculations of the Mössbauer signature of FeN_x moieties in different electronic states, they identify D1 to be a high-spin $Fe^{III}N_4C_{12}$ moiety with axial oxygen ligand (doublet with quadrupole splitting (QS) values of 0.9-1.2mms^{-1}) and D2 a low- or intermediate-spin $Fe^{II}N_4C_{10}$ moiety (QS=1.8-2.8mms^{-1}), both having a similar isomer shift. Limited by the different test instruments, and the two bimodal QS values we obtained are low, but we believe that the FeN_x site structure is similar to the structure they identified. Therefore, we refer to D1 and D2 as high-spin $Fe^{III}N_4$ moiety and low-spin $Fe^{II}N_4$ moiety, respectively.

Figure R12 clearly demonstrates that with decreasing nanoparticle size, the magnetic splitting spectrum of FeO_x transitions into a superparamagnetic spectrum and doublet. At room temperature, the 0.015Fe-NC-RT precursor consists of 90.2% FeOOH and 9.8% $FeCl_2 \cdot 4H_2O$. This aligns with the analysis reported in our manuscript, suggesting that $FeCl_2 \cdot 4H_2O$ undergoes oxidative hydrolysis at room temperature to yield $Fe(OH)_3$. At 400 °C, the FeO_x content in the product is 84.6%, with the remaining 15.4% attributed to the D2 sites, which correspond to the low-spin FeN_4 sites, corroborating our analytical results. Consequently, the oxidative hydrolysis occurring at room temperature is implicated in the high-temperature formation of FeO_x. Upon exceeding 600 °C, the FeO_x quantity diminishes to 73.4%. However, at temperatures above 700 °C, the sext signal is markedly reduced, leaving only the doublets associated with D1 and D2 peaks. D1 peak is presumed to represent a high-spin Fe^{III} species, potentially comprising high-spin $Fe^{III}N_4$ sites and superparamagnetic FeO_x nanoclusters. At 900 °C, the spectrum retains the two doublets, which are ascribed to high-spin FeN_4 and low-spin FeN_4 sites, respectively. In conclusion, our observations confirm the formation of FeO_x at lower temperatures (< 600 °C) and their subsequent transition to FeN_x sites at elevated temperatures.

To enhance quality of our manuscript, we have enriched the Supplementary Information with additional experimental data and included a detailed description (**Supplementary Fig. 42, Table 10, Note 5**).

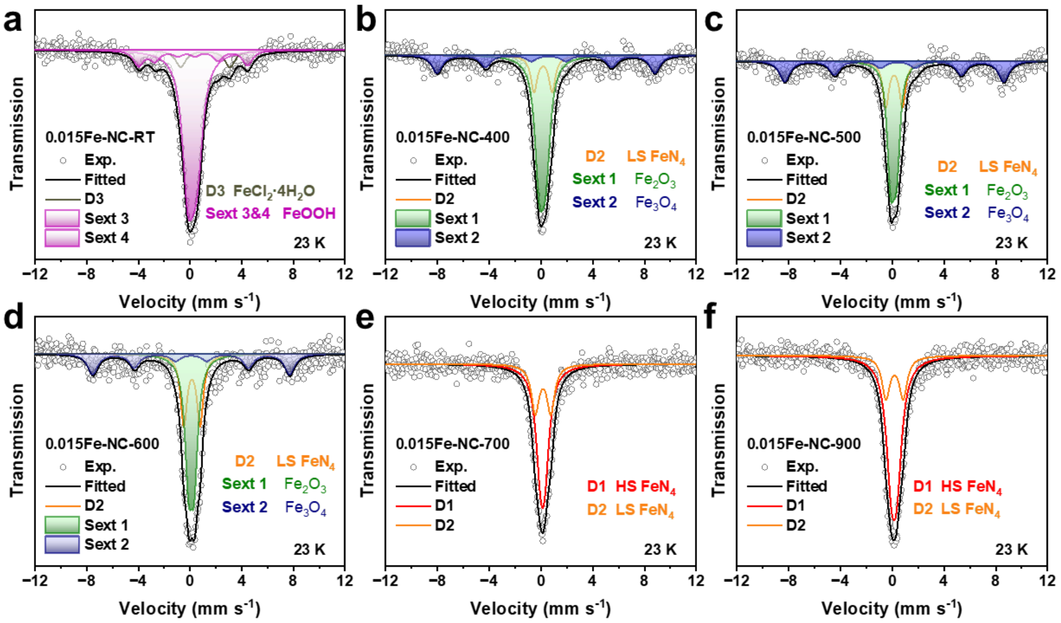

Figure R12. (a-f) The ^{57}Fe Mössbauer spectra of 0.015Fe-NC-*T* at 23 K.

Table R4. Average Mössbauer parameters of different samples and assignments for the iron species.

Samples	Comp.	RA%	IS mm s^{-1}	QS mm s^{-1}	LW mm s^{-1}	H Tesla	Assignment
0.015Fe-NC-RT	D3	9.8	1.1750	3.8090	0.9582	/	$FeCl_2 \cdot 4H_2O$
	Sext 3	71.2	0.1869	0.0898	0.9582	2.88	FeOOH
	Sext 4	19.0	0.0092	0.5023	0.9582	26.18	FeOOH
0.015Fe-NC-400	D2	15.4	0.1643	1.4009	0.6001	/	LS $Fe^{II}N_4$
	Sext 1	59.8	0.1445	0.0448	0.7460	2.13	Fe_2O_3
	Sext 2	24.8	0.5202	-0.2046	0.9582	52.21	Fe_3O_4
0.015Fe-NC-500	D2	26.6	0.1416	1.2593	0.6034	/	LS $Fe^{II}N_4$
	Sext 1	48.8	0.1274	0.0284	0.5512	1.93	Fe_2O_3
	Sext 2	31.7	0.3273	-0.2939	0.9582	52.53	Fe_3O_4
0.015Fe-NC-600	D2	26.6	0.1416	1.2593	0.6034	/	LS $Fe^{II}N_4$
	Sext 1	47.8	0.1405	0.0448	0.6760	2.08	Fe_2O_3
	Sext 2	25.6	0.1199	-0.0130	0.9582	47.34	Fe_3O_4
0.015Fe-NC-700	D1	67.9	0.1276	0.3223	0.8878	/	HS $Fe^{III}N_4$
	D2	32.1	0.1587	1.1987	0.7215	/	LS $Fe^{II}N_4$
0.015Fe-NC-900	D1	55.4	0.1383	0.2968	0.8959	/	HS $Fe^{III}N_4$
	D2	55.6	0.1704	1.1702	0.8925	/	LS $Fe^{II}N_4$

iv) To substantiate the synthesis proposition of the manuscript, the fabrication of Fe/N/C catalysts via acid gas-assisted pyrolysis should be attempted, to validate the study's conclusions.

Response iv:

Thanks for the value suggestion. To verify our conclusions, we introduced NH_4Cl and NH_4Br to the original synthetic base, which involved co-grinding the NC carrier, $FeCl_2 \cdot 4H_2O$, NH_4Cl, and NH_4Br uniformly (**Figure R13**). At around 300 ^oC, NH_4Cl and NH_4Br decompose to produce acidic gases HCl and HBr, which react with the formed FeO_x, causing the FeO_x nanoparticles to disintegrate, thus achieving the goal of reducing or even inhibiting the formation of FeO_x nanoparticles. As shown in **Figure R14**, it is not difficult to see that when NH_4Cl and NH_4Br are added, the characteristic peaks of FeO_x nanoparticles disappear, indicating that acidic gases effectively inhibit the formation of FeO_x, which is consistent with our conclusion. In addition, we also verified the specific excellent fuel cell performance of the treated Fe/N/C catalyst (**Figure R15**). Relevant work has also been submitted to Nature Communications (Manuscript ID: NCOMMS-23-61683-T), which is currently in Revision.

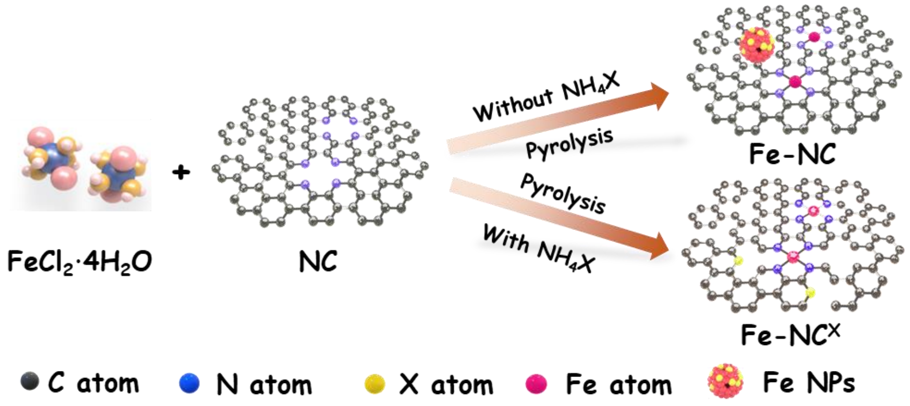

Figure R13. Schematic diagram of acid gas-assisted pyrolysis.

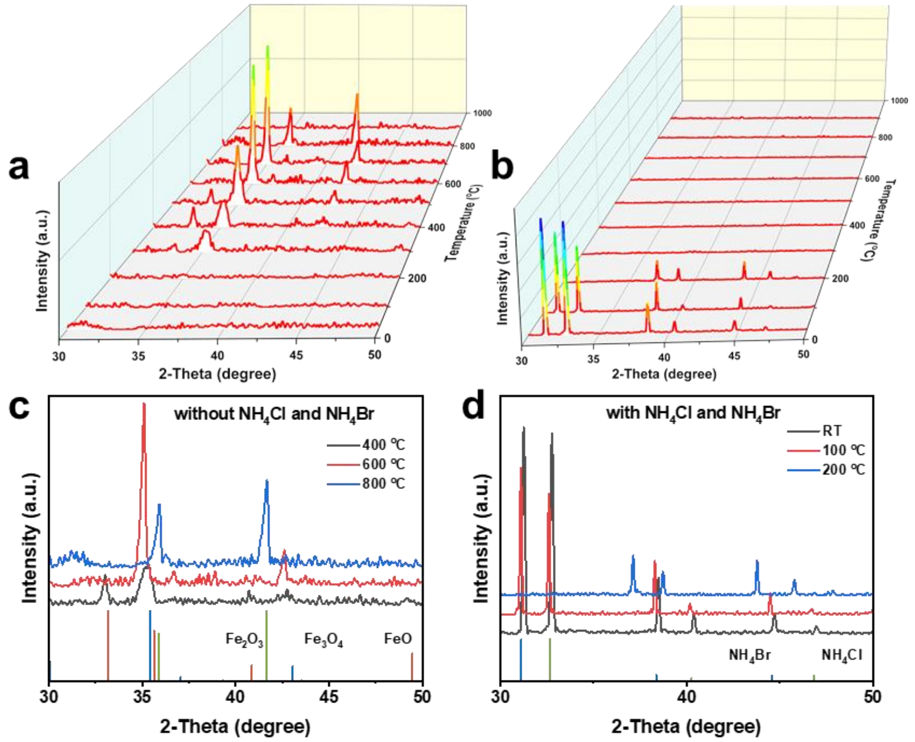

Figure R14. The *in-situ* heating XRD pattern of Fe-NC without (a, c) and with (b, d) NH_4Cl and NH_4Br-assisted pyrolysis.

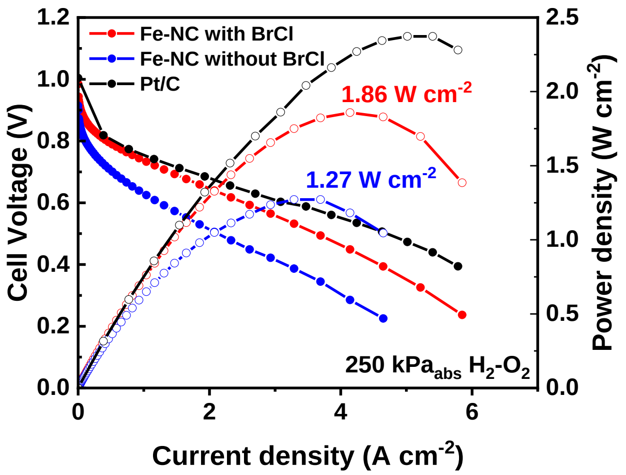

Figure R15. H_2-O_2 fuel cell *i-V* polarization and powder density plots record under absolute 2.5 bar of O_2 pressure. Anodes: Pt/C, 0.40 mg_{Pt} cm^{-2}, cathodes: Fe-N-C, 3.5 mg cm^{-2}, 100% RH, and 2.5 bar absolute H_2 pressure. Membrane: Nafion 211. Temperature: 80 °C. MEA area: 1.21 cm^2.

Reviewer #3 (Remarks to the Author):

This work of Yin et al. presents interesting observations regarding the formation of iron single atom catalysts supported on carbon. Although numerous types of SACs have been demonstrated, their formation mechanisms have been elusive. More recently, there have been attempts to better understand the process during pyrolysis and the reviewer finds that this work performing in-situ TEM nicely complements the knowledge that has been collected so far in the field. Thus, the reviewer recommends the publication of this work after the following questions and concerns have been addressed.

<u>**Response**</u>:

We are thankful to Reviewer #3 for their positive evaluation of our manuscript and thoughtful suggestions that have helped us improve our work.

1. LCF of XANES is misleading as it suggests that Fe-N_4 sites are formed even as low as 500 C (24%) and increased minorly up to 900C (44%). It does question whether this method is accurate enough to be valid. This also means that Fe-N4 sites are formed during the fusion and growth stage, but the discussion around the first half of this manuscript based on TEM data analysis suggests otherwise. The paper should present a consistent picture of the data that is collected by various means.

<u>**Response 1**</u>:

1) Reliability of Linear Combination Fitting (LCF) of XANES

Thanks for the value comment. The fitting details of Linear Combination Fitting (LCF) are given in Supplementary Note 3. LCF is a method used to analyze XANES spectra by decomposing the spectrum of an unknown sample into a linear combination of reference spectra from known compounds. This method assumes that the XANES spectrum of a mixture (or an unknown

compound) can be represented as a sum of the spectra of its constituent phases, each multiplied by a weighting factor corresponding to its concentration. Reference spectra was selected by combining XRD and TEM characterization. Due to the dehydration of FeOOH above 500 °C, which resulted in the formation of FeO_x. Thus, the samples treated at 500 °C, 700 °C, 800 °C, and 900 °C were fitted with Fe_2O_3, Fe_3O_4, FeO, $FeCl_2$, Fe foil and FePc until the best fitting effect was achieved, with all *R* factors being less than 1% (**Figure R16**, **Table R5**). The square-planar Fe–N_4 configuration of FePc is consistent with the Fe–N_4 configuration of pyrolytic Fe/N/C. It is assumed that the FePc component is the Fe–N_4 sites component that is generated during pyrolysis. The content of Fe_2O_3 decreased with increasing temperature, reaching a maximum at 700 °C; the content of Fe_3O_4 increased initially, peaked at 700 °C, and then vanished completely at 900 °C; the content of FeO and FePc increased gradually. The results are consistent with those of *in-situ* XRD and *in-situ* XPS experiments. Therefore, we believe that the results of LCF fitting are reliable.

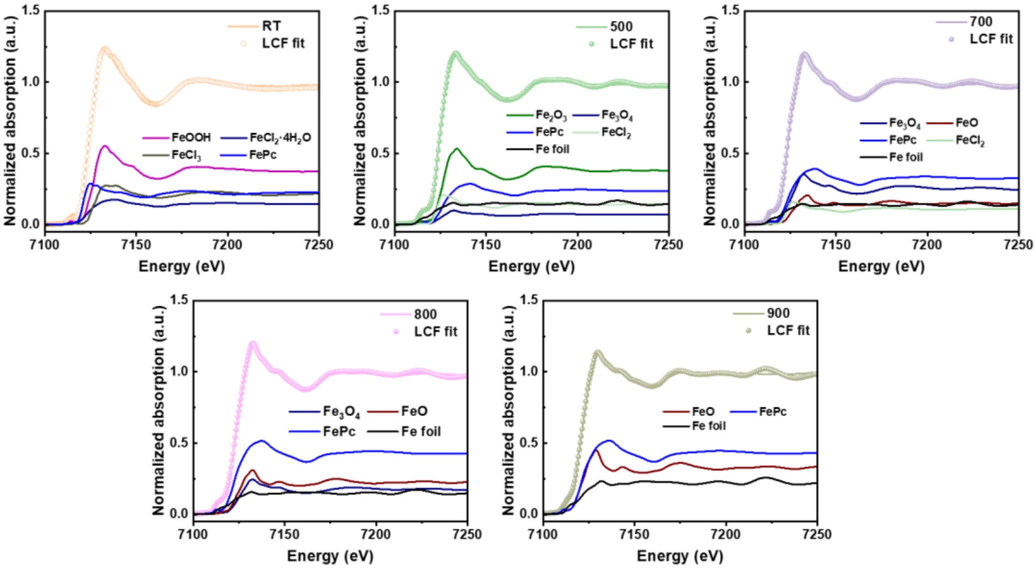

Figure R16. LCF fitting results of 0.015Fe-NC-*T*.

Table R5. LCF result of 0.015Fe-NC-*T*.

Content / Samples	FeO (%)	Fe_3O_4 (%)	Fe_2O_3 (%)	FePc (%)	Fe foil (%)	$FeCl_2$ (%)	*R*-factor
0.015FeNC-500	0	7.3	38.7	24.5	15.1	14.5	0.0003
0.015FeNC -700	15.1	25.9	0	33.4	14.5	11.0	0.0002
0.015FeNC -800	22.7	17.9	0	43.9	15.4	0	0.0005
0.015FeNC -900	33.0	0	0	44.1	22.9	0	0.0006

2) The formation temperature of FeN_x sites

We first analyze the formation temperature of FeN_x sites through the ORR performance of the material and the density of electrochemical active sites (determined by nitrite poisoning). As shown

in **Figure R17a-b**, when the pyrolysis temperature is 500 °C, we observed that the half-wave potential ($E_{1/2}$) of the material increased from 0.45 V to 0.63 V, indicating the formation of some active sites at 500 °C. The $E_{1/2}$ increased from 0.63 V to 0.75 V at 600 °C. As the pyrolysis temperature gradually rose to 900 °C, the $E_{1/2}$ of the material slowly increased from 0.75 V to 0.84 V. However, we observed that at 500 °C, the kinetic current density (j_k) of the material at 0.85 V and the TOF (obtained by nitrite poisoning) were low (**Figure R17 c**). We associated this with the electrochemical active sites density and found that the site density quantified by nitrite poisoning at 500 °C was only 0.08×10^{19} sites g^{-1}, while at 600 °C, the site density was 0.36×10^{19} sites g^{-1}, which is four times higher. Combined with the Mössbauer spectrum in **Figure R12**, we can analyze that the material treated at 500 °C contains 26.6% of D2 sites. According to Frédéric Jaouen et al., who identified the active sites of Fe-N-C catalysts by Mössbauer spectroscopy and XAS as shown in **Figure R18** [*Nat. Mater.*, *2021*, *4*, *10-19*], they think that there are mainly two types of sites in the material: the highly active but lowly stable Site S1 (D1) and the lowly active but highly stable Site S2 (D2). Therefore, we consider that the lowly active Site S2 (D2) might be a class of active sites that are insensitive to nitrite, and we discussed the drawbacks of the nitrite poisoning method in detail in ***Reviewer #3 Answer 2***. Peter Strasser et al. found that nitrite adsorption could lead to a 70-80% reduction in ORR activity and they suggested that nitrite poisoning mainly affects the sites that contribute the most to the ORR current, namely the highly active Site S1 [*Energy Environ. Sci.*, *2020*, *13*, *2480*]. This is consistent with our experimental results; we found that when the density of active $Fe-N_4$ sites significantly increased (at a pyrolysis temperature of 600 °C), the TOF and mass activity at 0.85 V showed a significant increase (**Figure R17 c-d**). Therefore, we believe that at 500 °C, a small amount of the less active Site S2 (D2) is formed, quantified by Mössbauer spectroscopy as approximately 26.6% (**Table R4**), which is similar to the results quantified by LCF (24%). At 600 °C, the formation of the highly active Site S1 (D1) begins.

Secondly, let's revisit the TEM (Transmission Electron Microscopy) section. Due to the resolution limits of the instrument, which prevent the direct observation of individual atoms, we think that during the pyrolysis process, the formation of nanoparticles, their coalescence growth, and decomposition occur. We also observed the migration of nanoparticles, which is believed to contribute to the ordering of the carbon layers. However, it is important to emphasize that we do not claim that FeN_x sites are formed only during the decomposition stage of nanoparticles, as the observed decomposition stage of nanoparticles occurs above 900 °C. In fact, as shown in **Figure R19a** (Supplementary Fig.21 in Supporting Information), we can see that some nanoparticles within the red dashed frame begin to decompose when the temperature is increased from 500 °C to 800 °C. Furthermore, through the aberration-corrected TEM images of the material at 500 °C, we observed FeO_x clusters (with a lattice spacing of 0.22 nm, which is larger than the 0.20 nm spacing of Fe(110), likely due to the inclusion of oxygen atoms in the lattice) and a small number of Fe single atoms. This demonstrates that at lower temperatures, some clusters that are not observable decompose to form a small number of single atomic FeN_x sites.

Finally, we can also deduce the quantitative results of LCF from the coordination number of Fe-N/O, that is, the average coordination number is determined by the six-coordination of $Fe-O_6$, the four-coordination of $Fe-N_4$, and the weight obtained from LCF. For example, at 500 °C, the $Fe-N_4$ sites account for 24%, and the rest are FeO_x (76%), thus, the average coordination number is 4*24%

+ 6*76%, which is 5.5, closely matching the fitted result of 5.7. This further illustrates that at 500 °C, there is a coexistence of a small amount of FeN_x sites and a large number of FeO_x species.

In summary, combined with TEM, XAS and other characterization results, we believe that some low-activity FeN_x sites begin to form at 500 °C, while high-activity FeN_x sites (nitrite poisoning sites) begin to form at 600 °C.

In order to improve the quality of our manuscripts, we have supplemented the corresponding parts.

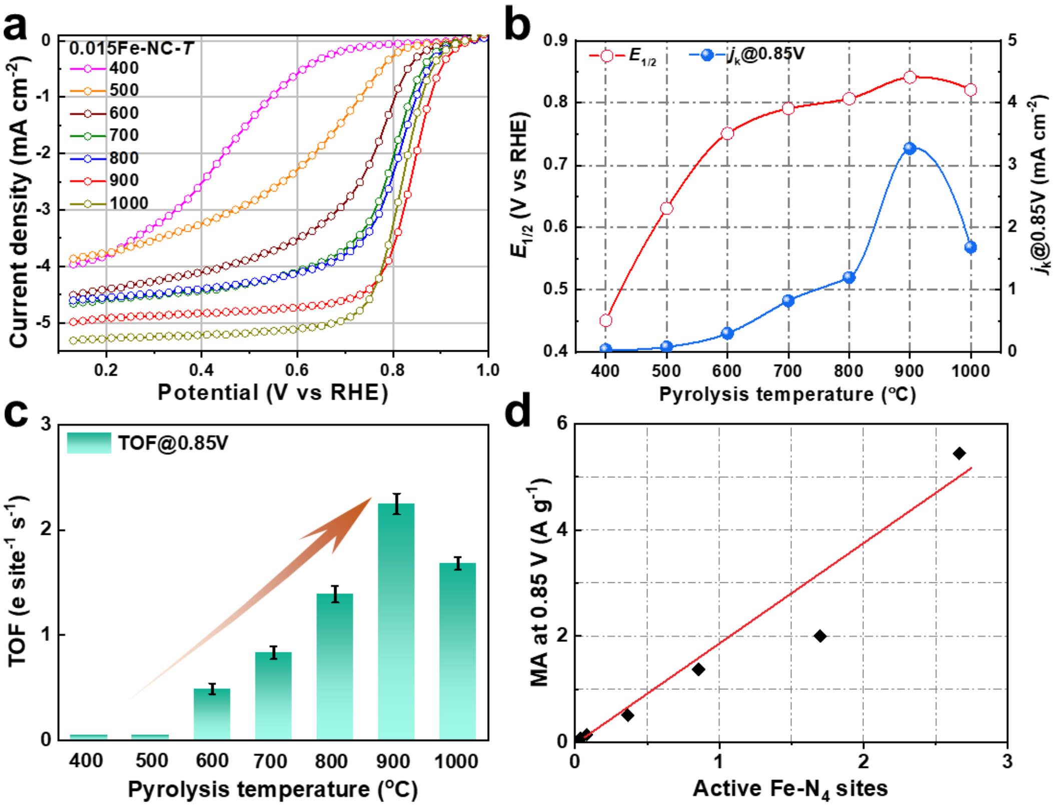

Figure R17. (a) ORR polarization curves; (b) $E_{1/2}$ and j_k@0.85V; (c) TOF at 0.85 V of 0.015Fe-NC-*T* catalysts; (d) Correlation between mass activity at 0.85 V and active $Fe\text{-}N_4$ sites.

Table R6. Summary of active $Fe–N_4$ sites by nitrite poisoning experiment.

Samples	active $Fe–N_4$ (sites g^{-1})	$TOF_{5.2}$@0.85V (e site $^{-1}$ s^{-1})
0.015Fe-NC-400	$0.04±0.005×10^{19}$	/
0.015Fe-NC-500	$0.08±0.01×10^{19}$	/
0.015Fe-NC-600	$0.36±0.08×10^{19}$	0.49±0.05
0.015Fe-NC-700	$0.86±0.10×10^{19}$	0.83±0.06
0.015Fe-NC-800	$1.70±0.15×10^{19}$	1.39±0.08
0.015Fe-NC-900	$2.66±0.20×10^{19}$	2.25±0.1
0.015Fe-NC-1000	$2.20±0.13×10^{19}$	1.68±0.06

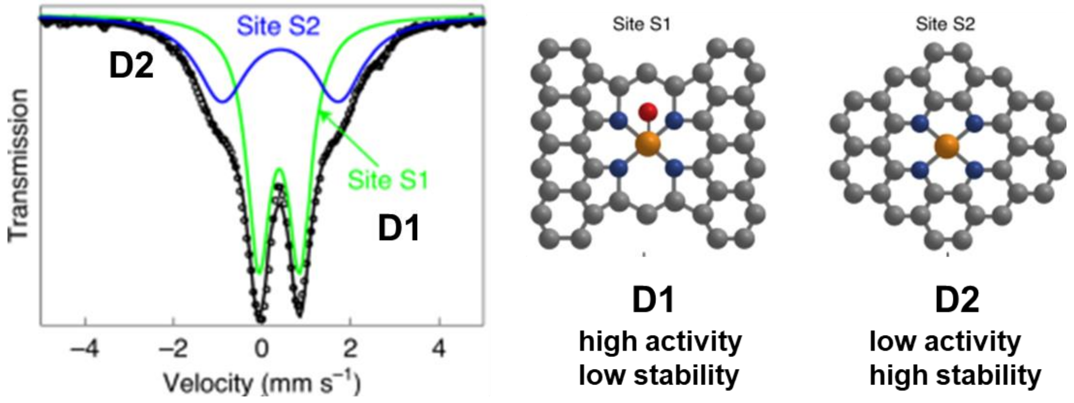

Figure R18. Site S1 (D1) and Site S2 (D2) identinated by Jouaen et al.

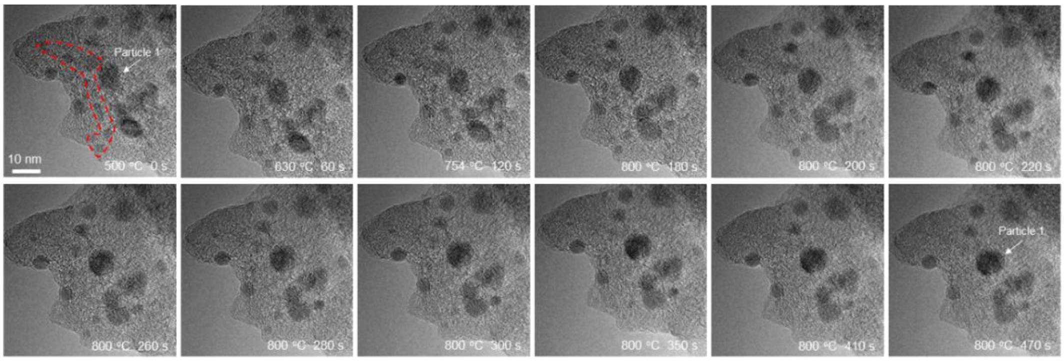

Figure R19. *In-situ* heating TEM experiments between 500 ^{o}C to 800 ^{o}C.

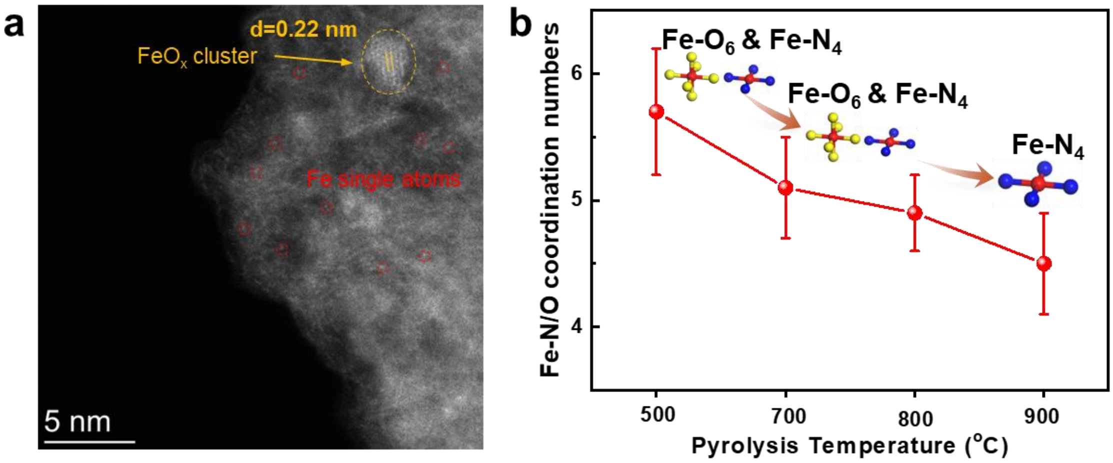

Figure R20. (a) The AC-HAADF-STEM image of 0.015Fe-NC-500; (b) The Fe-N/O coordination numbers as a function of the pyrolysis temperature.

2. How are the data related to Fig. 5b collected? FeN4 active sites are I assume by nitrite adsorption. But what about the inactive FeN4 and the inactive Fe species? How are they quantified?

Response 2:

Thanks for the value comment. The data of Fig.5b comes from Supplementary Table 8, and we explain the process of data processing below the table. In simple terms, the number of active Fe-N_4 sites is derived from the nitrite poisoning experiment (**Supplementary Table 7**), because the active sites obtained by nitrite poisoning are electrochemically accessible, so we believe that the active sites calculated by this method are the real active sites involved in the ORR reaction. The number of total Fe-N_4 sites is obtained by LCF-fitting results. Because XANES results are bulk results, the content of different components of the sample can be distinguished by LCF fitting, so it can be considered that LCF-fitting can obtain the total Fe-N_4 sites. Combining the number of total Fe-N_4 sites and the number of active Fe-N_4 sites, we can calculate the number of inactive Fe-N_4 sites. The number of total Fe atoms is obtained from ICP-MS data (**Supplementary Table 8**). Further combining the number of total Fe atoms and the number of total Fe-N_4 sites, we can calculate the number of inactive Fe species.

We list the calculation formula below **Supplementary Table 9**. [a]The data from Supplementary Table 6 nitrite poisoning method; [b]$N_{\mathrm{inactive\ Fe-N_4}} = \frac{x_{\mathrm{ICP}}}{M_{\mathrm{Fe}}} \times N_{\mathrm{A}} \times x_{\mathrm{LCF}} - N_{\mathrm{aciive\ Fe-N_4}}$, x_{icp} is the Fe weight ratio by ICP-MS from Supplementary Table 7, M_{Fe} is the molar mass of Fe, N_{A} is the Avogadro constant, x_{LCF} is the FePc content by LCF data from Supplementary Table 4; [c]$N_{\mathrm{inactive\ Fe\ species}} = N_{\mathrm{total\ Fe}} - N_{\mathrm{aciive\ Fe-N_4}} - N_{\mathrm{inaciive\ Fe-N_4}}$; [d]$N_{\mathrm{total\ Fe}} = \frac{x_{\mathrm{ICP}}}{M_{\mathrm{Fe}}} \times N_{\mathrm{A}}$.

Supplementary Table 7. Summary of $Q_{\mathrm{stripping}}$ and active Fe–N_4 sites.

Samples	$Q_{\mathrm{stripping}}$ (C g^{-1})	Δj_{m}@0.85V (A g^{-1})	active Fe–N_4 (sites g^{-1})	$TOF_{5.2}$@0.85V (e $site^{-1}$ s^{-1})
0.015Fe-NC-400	0.28	/	$0.04\pm0.005\times10^{19}$	/
0.015Fe-NC-500	0.63	/	$0.08\pm0.01\times10^{19}$	/
0.015Fe-NC-600	2.92	0.282	$0.36\pm0.08\times10^{19}$	0.49 ± 0.05
0.015Fe-NC-700	6.85	1.147	$0.86\pm0.10\times10^{19}$	0.83 ± 0.06
0.015Fe-NC-800	13.61	3.778	$1.70\pm0.15\times10^{19}$	1.39 ± 0.08
0.015Fe-NC-900	21.34	9.577	$2.66\pm0.20\times10^{19}$	2.25 ± 0.1
0.015Fe-NC-1000	17.63	5.688	$2.20\pm0.13\times10^{19}$	1.68 ± 0.06

Supplementary Table 8. ICP-MS data of 0.015Fe-NC-*T* samples.

Samples	Fe (wt%)	Fe ($\times10^{19}$ sites g^{-1})
0.015Fe-NC-500	1.52	16.39
0.015Fe-NC-700	1.51	16.28
0.015Fe-NC-800	1.50	16.18
0.015Fe-NC-900	1.52	16.39

Supplementary Table 9. The Fe atoms number of 0.015Fe-NC-T. (unit: $\times10^{19}$ sites g^{-1})

**Samples**	$N_{\text{active Fe–N4}}$[a]	$N_{\text{inactive Fe–N4}}$[b]	$N_{\text{inactive Fe species}}$[c]	$N_{\text{total Fe}}$[d]
0.015Fe-NC-500	0.08	3.94	12.37	16.39
0.015Fe-NC-700	0.85	4.58	10.85	16.28
0.015Fe-NC-800	1.70	5.40	9.08	16.18
0.015Fe-NC-900	2.66	4.56	9.17	16.39

It should be noted that the method for quantifying the active site density in the context of nitrite poisoning does not account for all active sites. This method, introduced by Kucernak *et al.* [*Nat. Commun., 2016, 7, 13285*], is depicted in **Figure R21a**. It is premised on the assumption that NO_2^- adsorbs onto the FeN_x site in a 1:1 ratio, forming NO* species (*denotes adsorption). At a low potential, each NO* can undergo a 5-electron reduction process and desorb, allowing for the quantification of NO_2^- adsorption from the reduction charge. Consequently, the site density (SD) of the FeN_x site can be calculated. The comprehensive testing procedure is outlined in **Figure R21b**. The activity of the non-metallic N/C catalyst remains unchanged pre- and post-poisoning, with no observable reduction peak signal in the low potential range for NO_2^- reduction. Therefore, this method does not specifically adsorb N/C and C. Yet, the Fe-N/C catalyst exhibits a marked decrease in activity after NO_2^- adsorption, with a corresponding reduction peak emerging in the low potential range. The adsorbed NO is subsequently reduced electrochemically. Following this reduction, the activity reverts to its initial non-toxic state, indicative of reversible adsorption-desorption of NO. By assessing the reduction charge within the low potential zone (the potential range is about -0.1 to 0.3 V vs. RHE), one can calculate the specific adsorption value of NO_2^- and determine the SD of the FeN_x site.

Nonetheless, NO_2^- poisoning does not lead to complete catalyst deactivation. Despite the poisoning, Fe-N/C maintains a superior performance compared to N/C, as evidenced in **Figure R21b**. Moreover, the mass activity of the 0.015Fe-NC-900 catalyst diminishes by 85% following nitrite adsorption, yet it sustains a robust ORR performance (**Figure R22**). This implies that nitrite may only target a subset of exposed sites, yielding a lower quantification result. Peter Strasser *et al.* [*Energy Environ. Sci., 2020, 13, 2480*] have also highlighted this aspect, suggesting that nitrite adsorption significantly impairs the catalyst's ORR capability but does not entirely inhibit ORR activity. This results in a 70-80% reduction in ORR activity across the pertinent potential range (0.8-0.9 V), indicating a variety of sites involved in ORR activity, with nitrite poisoning primarily affecting those contributing most to the ORR current.

Due to experimental constraints, the implementation of CO-cryo adsorption experiments was not feasible. Nitrite assay has emerged as a widely accepted surrogate for determining the active site density in Fe-N-C catalysts. While the resulting quantitative measures may be conservative, they nonetheless capture the majority of active sites. These data provide a reliable reference point for comparing the density of catalytic sites.

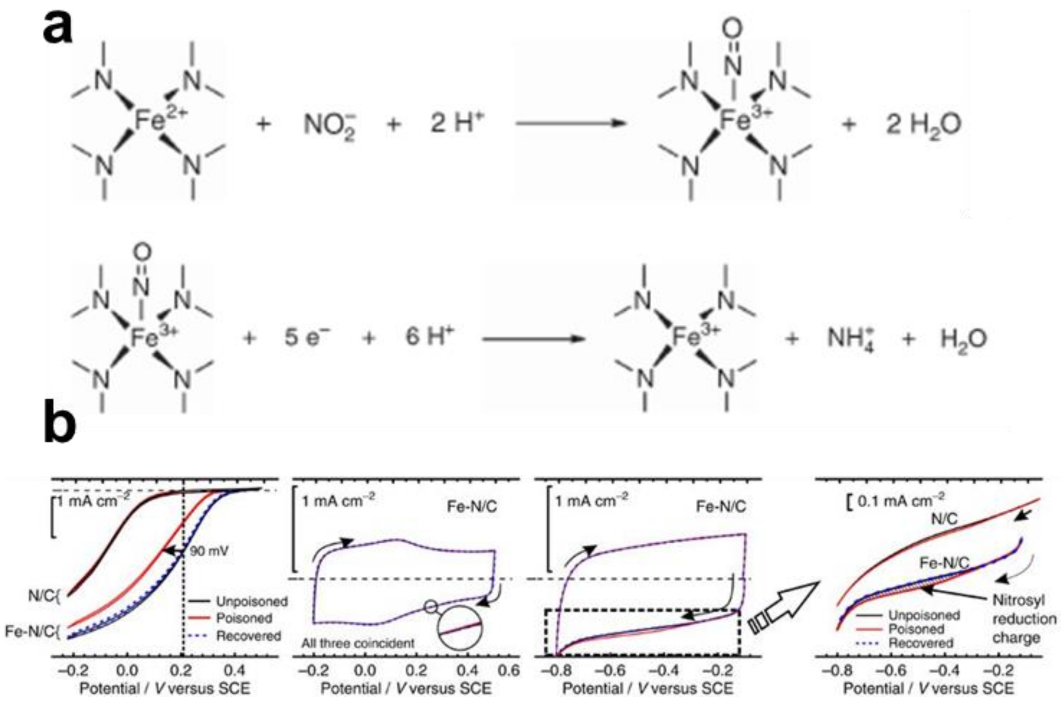

Figure R21. Principle of nitrite poisoning method. (a) Reaction mechanism; (b) ORR performance and cycle voltammetry of catalyst before, during and after nitrite adsorption.

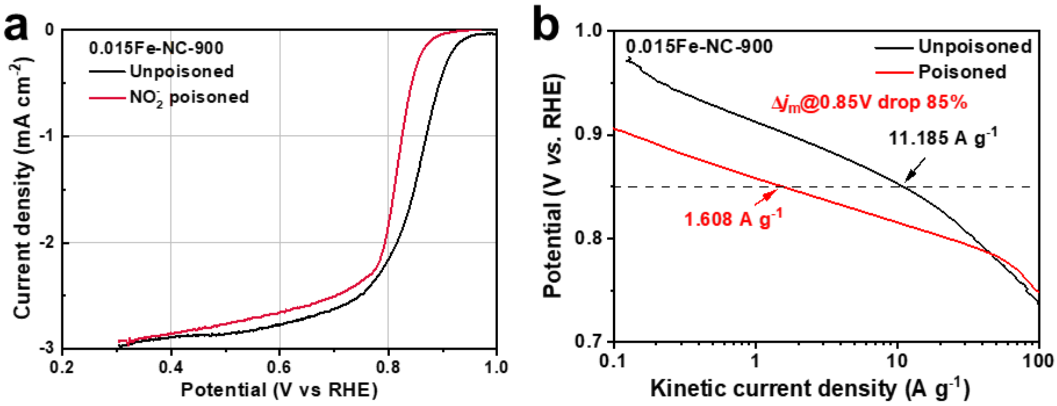

Figure R22. (a) ORR performance of 0.015Fe-NC-900 catalyst before and after nitrite adsorption; (b) ORR activity decay rate.

3. Figure 3c – shows the incorporation of iron atoms into the nitrogenated carbon structure leading to more ordered graphite sheets. How can we tell this is the right sequence of events? What if the ordered carbon is formed first by the catalytic action of iron (known to pyrolyze hydrocarbons to make graphites, carbon nanotubes, etc.) and then the iron single atoms become anchored by the N atoms?

Response 3:

Thank you for the insightful comment. According to the reviewers, the catalysis of graphene and carbon nanotubes by metal nanoparticles is a widely observed phenomenon. Renu Sharma and colleagues have elucidated the growth characteristics of carbon nanotubes on catalyst nanoparticles

using environmental transmission electron microscopy (ETEM) and reactive molecular dynamics (RMD) simulations [*J. Catal., 2017, 349, 149-155*]. As depicted in **Figure R23a**, they posited that the carbon precursor decomposes on the catalyst surface, with some of the resulting carbon diffusing into the particles, forming metal carbides. With the formation of carbon nanotubes and their partial coverage of the particle surface, carbon diffusion into the particles may be hindered, rendering the carbide phase unstable. Consequently, a carbon-deficient region is formed beneath the nanotube edge during growth. This carbon gradient serves as a driving force, causing carbon atoms on the particle to migrate to the surface and contribute to nanotube formation. In another study, Yan Li and colleagues detected partial melting of the catalyst via aberration-corrected ETEM [*CCS Chem., 2021, 3, 154-167*], consisting of crystalline (solid) and disordered (molten) regions. The nanotube cap was nucleated at the liquid domain within a partially molten catalyst particle, as illustrated in **Figure R23b**. Both studies corroborate the mechanism by which Co nanoparticles facilitate the formation of carbon nanotubes from hydrocarbons (e.g., CH_4, C_2H_2), whereby carbon infiltration results in two distinct regions: carbon-rich and carbon-poor. This gradient propels the diffusion of carbon atoms on the particle to the surface, culminating in carbon nanotube synthesis.

Our work differs in that the carbon source is nitrogen-doped carbon, and the nanoparticles are iron oxides. We have also analyzed the potential mechanism underlying the formation of carbon layers during nanoparticle decomposition. **Figure R24** showcases the evolution of the adjacent carbon layer within the orange virtual frame as the nanoparticles decompose. We note that initially, a single carbon layer encases the nanoparticles. After 30 seconds of treatment, a bilayer forms, which then becomes a trilayer after 60 seconds, while the nanoparticle size diminishes, indicative of decomposition. It is conceivable that the FeO_x nanoparticle surface may be reduced to metallic Fe atoms. The amalgamation of these Fe atoms with carbon atoms on the surface results in the formation of carbon-rich and carbon-poor areas, prompting carbon migration toward the surface and the generation of an ordered carbon layer. Concurrently, Fe atoms diffuse outward, likely driven by local pressure disparities caused by the pyrolysis temperature and gases produced during the carbothermal reaction. During migration on the carbon substrate, these iron atoms readily bind to nitrogen sites, forming FeN_x sites.

In response to the reviewer's query regarding the ordering of carbon and the formation of FeN_x sites, we observe in **Figure R24** that nanoparticle decomposition coincides with an increase in carbon layering. We think that these processes occur concurrently, making it challenging to discern a sequential order.

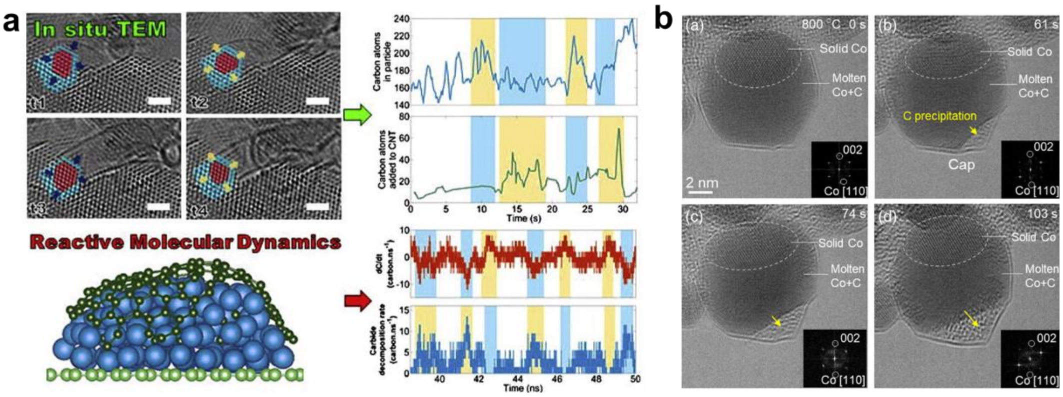

Figure R23. (a) *In-situ* growth of carbon nanotubes on Co nanoparticles, Acetylene (C_2H_2) and Co_xMo_{1-x}/MgO were used as carbon precursors and a catalyst-support system, respectively; (b) Time-sequenced ETEM images of a partial molten Co nanoparticle during the growth of carbon cap at 800 °C, Co nanoparticles and CH_4 were used as catalyst and carbon precursors, respectively.

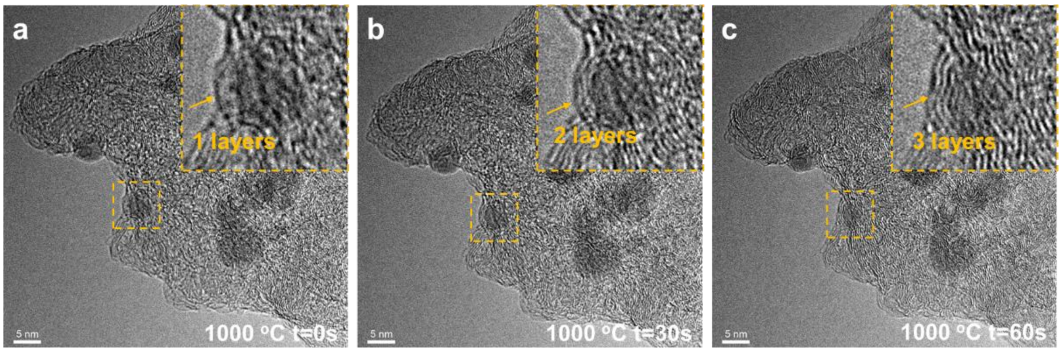

Figure R24. The change of nanoparticles adjacent to the carbon layer.

4. Why are the XRD reflections lacking at 1000C when metallic Fe nanoparticles are formed?

Response 4:

Thank you for your valuable feedback. We have analyzed the correlation between temperature, the quantity of active sites, and ORR performance, as depicted in **Figure R25**. Our findings indicate that the number of active sites and their corresponding activity escalate with increasing temperature, reaching an apex at 900 °C. However, a subsequent elevation to 1000 °C results in a decline in both the number of active sites and their activity. This pattern is consistent with the typical temperature range for synthesizing Fe-N-C catalysts reported in the literature, deemed optimal between 900 and 1000 °C [*Nano Energy, 2022, 100, 107440*; Nat Catal, 2022, 5, 311-323; *Nat Catal, 2019, 2, 259-268*; *Angew Chem Int Ed, 2023, 62, e202306166, etc.*]. Furthermore, due to the instrumental limitation of 900 °C for temperature, we set this as the maximal threshold for *in-situ* XRD and XAS experiments. Therefore, our investigations were conducted at this upper limit.

We have included additional XRD data for the 0.015Fe-NC-1000 material synthesized at 1000 °C. **Figure R26** reveals that upon elevating the temperature to 1000 °C, the characteristic peak of Fe_3O_4 emerges, signifying the oxidation of metallic Fe to Fe_3O_4 upon exposure to air. This observation

also implies that an increase in temperature beyond 900 °C promotes nanoparticle aggregation, which adversely affects ORR performance.

The nanoparticle aggregation at 1000 °C stems from the depletion of N sites. As illustrated in **Figure R27a**, the graphitic N content in the 0.015Fe-NC-1000 material prepared at 1000 °C is markedly higher than in other materials, as referenced in **Table R7**. Additionally, the N content diminishes from approximately 7.5 wt% to around 4.0 wt%, as shown in **Figure R27b** and **Table R8**. This suggests that the N component is lost at 1000 °C. Since FeN_x sites require an abundance of N sites to anchor Fe atom formation, the loss of N content leads to a weakened ability of the carbon support to secure Fe atoms, culminating in their aggregation into nanoparticles.

To enrich the manuscript's content, we have supplemented the relevant data in the Supplementary Information (Supplementary Fig. 5, 14, 15, Table 3, and the corresponding description).

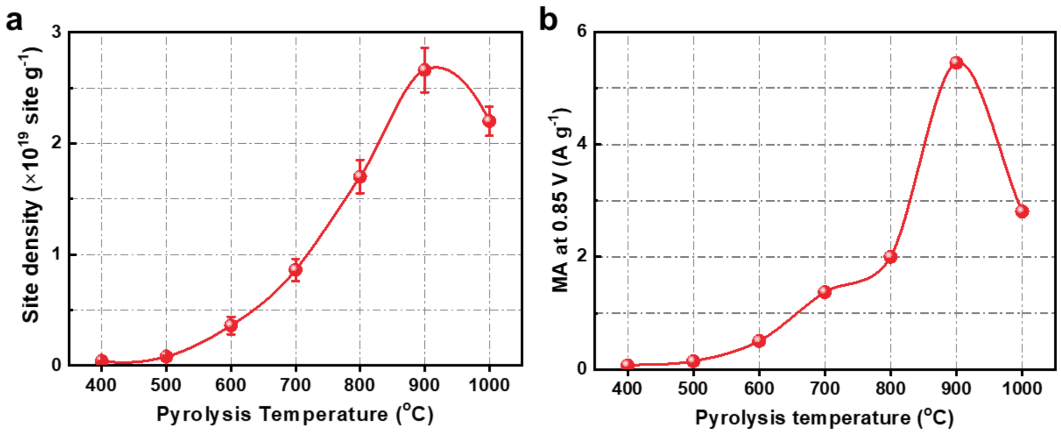

Figure R25. (a) The site density as a function of the pyrolysis temperature; the MA at 0.85 V as a function of the pyrolysis temperature.

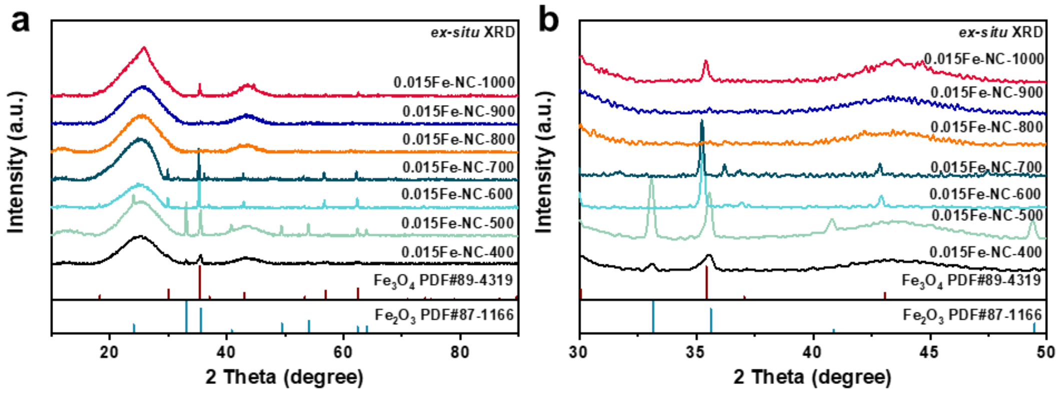

Figure R26. *Ex-situ* XRD patterns of 0.015Fe-NC-*T* samples obtained after thermal activation at different temperatures.

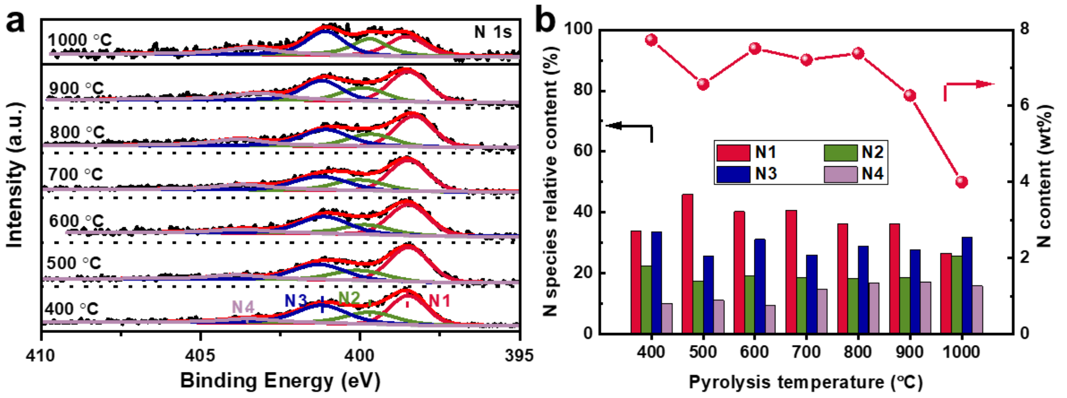

Figure R27. XPS analysis of 0.015Fe-NC-*T* samples. (a) XPS N 1s spectra; (b) Fraction of various types of nitrogen and total N content.

Table R7. XPS data for the surface N species of 0.015Fe-NC-*T* samples.

Samples	*N1 / %	*N2 / %	*N3 / %	*N4 / %
	398.48 eV	399.68 eV	401.21 eV	403.48 eV
0.015Fe-NC-400	33.95	22.46	33.60	9.99
0.015Fe-NC-500	45.87	17.33	25.72	11.08
0.015Fe-NC-600	40.26	19.14	31.11	9.49
0.015Fe-NC-700	40.77	18.58	26.00	14.66
0.015Fe-NC-800	36.27	18.21	28.79	16.74
0.015Fe-NC-900	36.31	18.64	27.83	17.21
0.015Fe-NC-1000	26.51	25.65	31.94	15.90

* N1: Pyridinic N; N2: Pyrrolic N; N3: Graphitic N; N4: Oxidic N.

Table R8. XPS data for the surface species for the bulk species of 0.015Fe-NC-T samples.

Samples	C / wt%	N / wt%	O / wt%	Fe / wt%	Zn / wt%
0.015Fe-NC-400	78.87	7.73	5.45	1.06	6.89
0.015Fe-NC-500	77.54	6.57	8.09	1.77	6.03
0.015Fe-NC-600	78.00	7.51	5.32	1.51	7.66
0.015Fe-NC-700	79.56	7.21	5.74	1.36	6.13
0.015Fe-NC-800	80.16	7.38	6.00	1.54	4.92
0.015Fe-NC-900	83.34	6.27	6.08	1.75	2.56
0.015Fe-NC-1000	87.43	4.00	6.59	1.57	0.42

The atomic percentage (at%) measured by XPS to the weight percentage (wt%) by following equation:

$$x\ \mathrm{wt\%} = \frac{\mathrm{M_x} \times x\ (\mathrm{at\%})}{12.01 \times \mathrm{C}\ (\mathrm{at\%}) + 14.01 \times \mathrm{N}\ (\mathrm{at\%}) + 16.00 \times \mathrm{O}\ (\mathrm{at\%}) + 65.38 \times \mathrm{Zn}\ (\mathrm{at\%}) + 55.85 \times \mathrm{Fe}\ (\mathrm{at\%})}$$

Where 12.01, 14.01, 16.00, 65.38 and 55.85 are the atomic mass of Fe, O, N, and C, respectively.

5. What is NC? It is just mentioned at the start of the discussion without a clear explanation as to what it is.

Response 5:

Thanks for the value comment. NC refers to nitrogen-doped carbon substrate. We have modified NC to nitrogen-doped carbon substrate (NC) in line 102 of the manuscript.

"$FeCl_2 \cdot 4H_2O$ (with a Fe loading of 1.5 wt%) and nitrogen-doped carbon substrate (NC) were pulverized and combined,"

6. In the introduction, the manuscript states that Fe site loadings demonstrated so far remain at a very low level, < 3 wt%. However, there have been demonstrations, such as Nature Communications volume 10, Article number: 1278 (2019), that go above such loading. There are probably more articles that demonstrate Fe loading > 3 wt% than any other element. The authors should revise this statement or clarify what their original intentions are.

Response 6:

Thank you for the insightful comment. We have analyzed the performance of H_2-air proton-exchange membrane fuel cells (PEMFCs) and the iron (Fe) content in high-performance Fe-N-C catalysts over recent years, as detailed in **Table R9**. Upon examination, it is evident that the air performance of the Fe-N-C catalysts with an Fe content exceeding 3 wt% (highlighted by the red dotted box) is moderate. In contrast, the Fe content in high-performance Fe-N-C catalysts typically remains below 3 wt%. It is noteworthy that numerous studies have reported on high-Fe-loading single-atom catalysts, as presented in **Table R10**. Despite these catalysts boasting significant single-atom Fe loadings, the publications provide only the oxygen reduction reaction (ORR) performance under alkaline conditions, without data on H_2-air PEMFCs performance.

In response to the reviewer's recommendations, we have revised the relevant text to read: "The majority of Fe/N/C catalysts that exhibit superior performance in PEMFCs generally contain less than 3 wt% Fe[8, 9, 20-23]. While there are reports of catalysts with Fe loadings in excess of 3 wt%, these have not demonstrated outstanding performance in PEMFCs applications[24-26]."

Table R9. H_2-air PEMFCs performance and Fe loading of Fe-N-C catalysts reported in recent years.

Catalysts	H_2-air PEMFCs / W cm^{-2}	Fe loading / wt%	Refs
Fe-NC$^{\Delta\text{-DCDA}}$	~0.42	7.00	*Nat. Catal., 2022, 5, 311-323*
Fe/N/C(4mIm)-OAc	0.47	3.49	*Adv. Funct. Mater.* 2021, 31, 2009645
TPI@Z8(SiO_2)-650-C	0.42	2.8	*Nat. Catal.* 2019, 2, 259
SA-FeNx-ZIF8-PCM	0.60	2.11	*Nano Energy* 2021, 83, 105734
Fe_g-NC/Phen	0.71	1.84	Energy Environ. Sci., 2022, 15, 3033-3040
Fe-N-C-FG	~0.72	0.73	*Nat. Catal.*, 2023, 6, 1215-1227
Fe-AC	~0.60	1.75	*Nat. Energy*, 2022, 7, 652-663
FeNC-CVD-750	~0.37	2.00	*Nat. Mater.*, 2021, 20, 1385-1391

Table R10. High-loading Fe-N-C catalysts and their performances in alkaline ORR.

**Catalysts**	**$E_{1/2}$ in alkaline / V**	**Fe loading / wt%**	**Refs**
Fe-NC SAC	0.90	8.9	*Nat. Commun., 2019, 10, 1278*
Fe_{SA}-N-C	0.90	3.46	*Nat. Commun., 2020, 11, 2831*
Fe-SA-NSFC	0.91	15.3	*Nat. Commun., 2020, 11, 5892*
FeSA-SeNC	0.90	18.3	*Small, 2023, 2308530*
FeSA-G	0.80 (Acid)	7.7	*Adv. Sci., 2019, 6, 1802066*

REVIEWER COMMENTS

Reviewer #1 (Remarks to the Author):

The revised manuscript aims to elucidate the single Fe site formation derived from FeOx as precursors using in-situ TEM imaging and other relevant characterization. The experimental results are interesting and present the dynamic transition of FeOx nanoparticles to single Fe sites. As a new reviewer to read the manuscript for the first time, the following suggestions are provided to the authors, which could be essential for the benefit of the field.

(1) The conversion of Fe2O2 to a single Fe site had been successfully demonstrated. The authors provide a deep understanding to elucidate the critical process in this work. However, the theoretical knowledge concerning the thermodynamic and kinetic process of the conversion is insufficient in this work. For example, what is the primary driving force, and what is the critical factor to limit the transition? Therefore, the relevant computational studies and simulations are necessary to provide meaningful understanding during the active site formation. In particular, the authors indicated that 7 nm is the critical value to decompose FeOx particles for subsequent active formation. What are the influences from other factors, such as heating temperature, atmosphere, and local environments.

(2) Frédéric Jaouen et al reported there are two types of FeN4 sites in Fe/N/C catalysts denoted as S1 and S2 sites (Nature Catalysis volume 4, pages 10–19 ,2021). The authors may further elucidate how the experimental conditions affect the formation of these two active sites during the FeOx conversion to single metal sites.

(3) Currently, Fe/N/C catalysts face grand stability challenges. The authors may explain the possible degradation mechanisms of the FeOx-derived catalysts and the possible strategies to enhance catalytic stability under ORR conditions.

Reviewer #2 (Remarks to the Author):

The authors have addressed this reviewer's concern. The paper can be accepted for publication.

Reviewer #3 (Remarks to the Author):

All concerns have been addressed in their communication with the reviewers and reflected in the revised manuscript.

Reviewers’ comments:

Reviewer #1 (Remarks to the Author):

The revised manuscript aims to elucidate the single Fe site formation derived from FeOx as precursors using in-situ TEM imaging and other relevant characterization. The experimental results are interesting and present the dynamic transition of FeOx nanoparticles to single Fe sites. As a new reviewer to read the manuscript for the first time, the following suggestions are provided to the authors, which could be essential for the benefit of the field.

(1) The conversion of Fe2O2 to a single Fe site had been successfully demonstrated. The authors provide a deep understanding to elucidate the critical process in this work. However, the theoretical knowledge concerning the thermodynamic and kinetic process of the conversion is insufficient in this work. For example, what is the primary driving force, and what is the critical factor to limit the transition? Therefore, the relevant computational studies and simulations are necessary to provide meaningful understanding during the active site formation. In particular, the authors indicated that 7 nm is the critical value to decompose FeOx particles for subsequent active formation. What are the influences from other factors, such as heating temperature, atmosphere, and local environments.

(2) Frédéric Jaouen et al reported there are two types of FeN4 sites in Fe/N/C catalysts denoted as S1 and S2 sites (Nature Catalysis volume 4, pages 10–19 ,2021). The authors may further elucidate how the experimental conditions affect the formation of these two active sites during the FeOx conversion to single metal sites.

(3) Currently, Fe/N/C catalysts face grand stability challenges. The authors may explain the possible degradation mechanisms of the FeOx-derived catalysts and the possible strategies to enhance catalytic stability under ORR conditions.

Reviewer #2 (Remarks to the Author):

The authors have addressed this reviewer's concern. The paper can be accepted for publication.

Reviewer #3 (Remarks to the Author):

All concerns have been addressed in their communication with the reviewers and reflected in the revised manuscript.

Responses to Reviewers' Comments

We sincerely appreciate the reviewers for the expert and valued comments and constructive suggestions which greatly help us to improve the quality of this submission (NCOMMS-23-59721B). In the following, we carefully respond the reviewers' comments point by point (texts in blue), and describe the corresponding revisions made on the manuscript and Supplementary Information (orange font). The figures in this Response Letter are named as Figure R**.

Reviewer #1 (Remarks to the Author):

The revised manuscript aims to elucidate the single Fe site formation derived from FeO_x as precursors using in-situ TEM imaging and other relevant characterization. The experimental results are interesting and present the dynamic transition of FeO_x nanoparticles to single Fe sites. As a new reviewer to read the manuscript for the first time, the following suggestions are provided to the authors, which could be essential for the benefit of the field.

Response:

We sincerely thank you for taking the time to evaluate our manuscript and for your positive and constructive comments.

(1) The conversion of Fe_2O_2 to a single Fe site had been successfully demonstrated. The authors provide a deep understanding to elucidate the critical process in this work. However, the theoretical knowledge concerning the thermodynamic and kinetic process of the conversion is insufficient in this work. For example, what is the primary driving force, and what is the critical factor to limit the transition? Therefore, the relevant computational studies and simulations are necessary to provide meaningful understanding during the active site formation. In particular, the authors indicated that 7 nm is the critical value to decompose FeO_x particles for subsequent active formation. What are the influences from other factors, such as heating temperature, atmosphere, and local environments.

Response 1:

Thank you for your insightful comments and suggestions. We appreciate the opportunity to address these important aspects of our study. Here is a detailed response:

We acknowledge the need for a deeper exploration of the thermodynamic and kinetic processes governing the conversion of FeO_x to single Fe sites. The primary driving force for this conversion lies in the thermodynamic stability of the single Fe sites compared to the bulk FeO_x particles. This stability is influenced by factors such as Gibbs free energy changes during the conversion process and the interaction of Fe atoms with the support material.

The critical factor limiting the transition is the energy barrier associated with breaking down the FeO_x lattice to form isolated Fe sites. This involves overcoming the kinetic barriers related to atom diffusion and rearrangement on the support surface. We think that the key factors to break the energy barrier are the pyrolysis temperature and the size of the FeO_x nanoparticles.

To provide a meaningful understanding of these processes, we agree that computational studies and simulations are essential. Density functional theory (DFT) calculations can help elucidate the energy landscape of Fe site formation, while molecular dynamics (MD) simulations can provide insights into the kinetics of the transformation process. In our previous manuscript, we have proved that $Fe_{13}O_{13}$ clusters could release a Fe atom into the N_4-C defect under 1180 K by MD simulation **(Figure R1**, Supplementary Dynamic Figure-Reaction). Besides, we have also proved that the removal of oxygen atoms from $Fe_{13}O_{13}$ clusters will significantly increase the thermodynamic trend of the conversion reaction from FeO_x to Fe-N_4 sites (**Figure R2**).

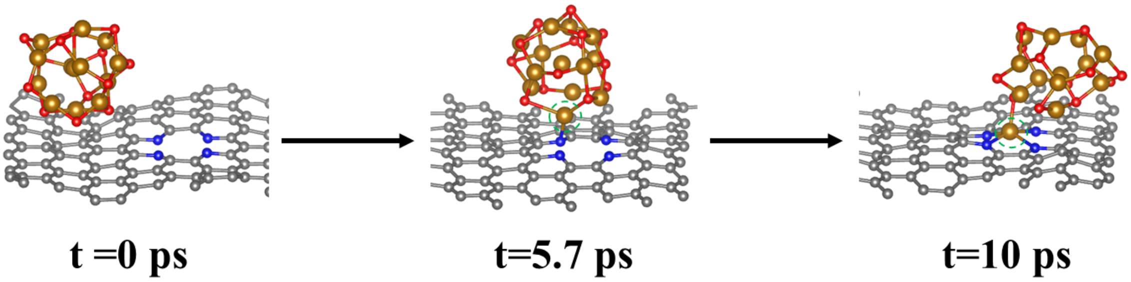

Figure R1. Time-series images during $Fe_{13}O_{13}$ to Fe-N_4 site.

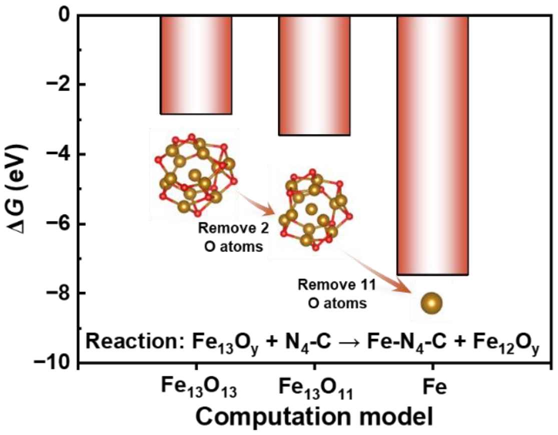

Figure R2. The ΔG of different transformation reactions.

Based on our experimental results, we have demonstrated that the removal of oxygen atoms from the surface of $Fe_{13}O_{13}$ is due to the carbothermal reaction. Therefore, we calculated the thermodynamic Gibbs free energy change for the reaction between Fe_nO_n with varying atomic numbers and carbon to form CO at different temperatures. As shown in **Figure R3**, we fixed n at 4 and 13 to simulate different sizes of FeO_x nanoparticles. The carbon atom at the defect site reacts with an oxygen atom from Fe_nO_n, breaking the Fe-O bond and generating CO and Fe_nO_{n-1}. Interestingly, the reaction involving Fe_4O_4 clusters requires a higher Gibbs free energy change (ΔG),

likely because the small number of atoms makes it difficult to break the Fe-O bond (**Figure R4**). Moreover, the Δ*G* values for the reaction at different temperatures are essentially the same. This indicates that the pyrolysis temperature does not overcome the thermodynamic barrier of the conversion reaction but rather accelerates the reaction kinetics, thereby increasing the conversion rate of FeO_x to $Fe\text{-}N_4$ sites.

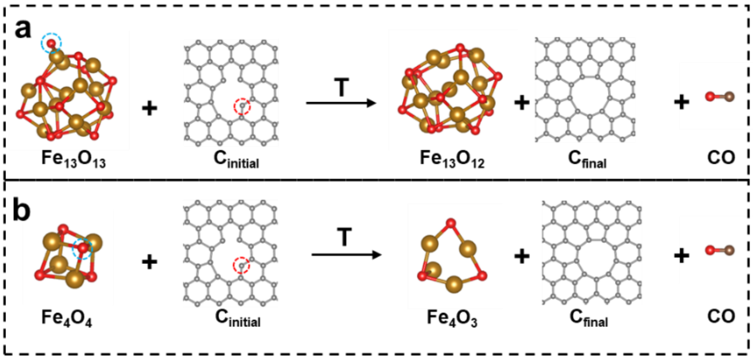

Figure R3. A schematic diagram of the reaction of Fe_nO_n with different atomic numbers with C to form CO, the equation: $Fe_nO_n + C_{initial} \rightarrow Fe_nO_{n-1} + C_{final} + CO$. (a) $Fe_{13}O_{13}$, (b) Fe_4O_4.

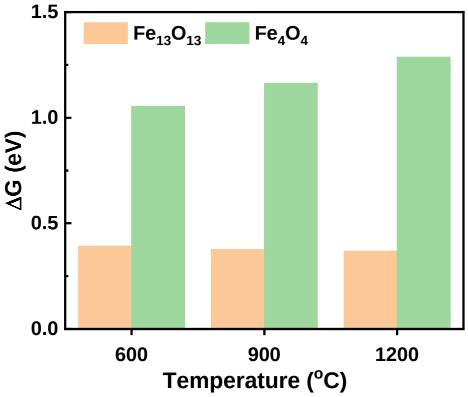

Figure R4. The Gibbs free energy changes (ΔG) of two reactions at different temperatures.

The release of a Fe atom from the nanoparticles involves the structural stability of the nanoparticles themselves. A very important metric for stability of nanoparticle is the cohesive energy (E_{coh}), which can be written as

$$E_{coh} = |E_{FenOn}/n - (E_{Fe} + E_O)| \qquad (1)$$

$$E_{coh} = |E_{Fen}/n - E_{Fe}| \qquad (2)$$

where E_{FenOn} and E_{Fen} are the electronic energies of the Fe_nO_n and Fe_n nanoparticles, respectively, E_{Fe} and E_O are energies of free Fe and O atoms, respectively. In this convention, a lower cohesive energy indicates a weaker cohesion of the nanoparticle, i.e. thermal decomposition. Obvious, whether Fe_nO_n or Fe_n models (n denotes the number of atoms), the E_{coh} increases with the increase of n, indicating that the separation of nanoparticles into single atoms requires greater energy (**Figure R5**). This is also consistent with our experimental results, that is, we observed that nanoparticles less than 7 nm were completely decomposed, forming $Fe\text{-}N_x$ sites or sintered into larger

nanoparticles by Ostwald ripening. The nanoparticles larger than 10 nm are not completely decomposed and tend to be sintered to form larger nanoparticles.

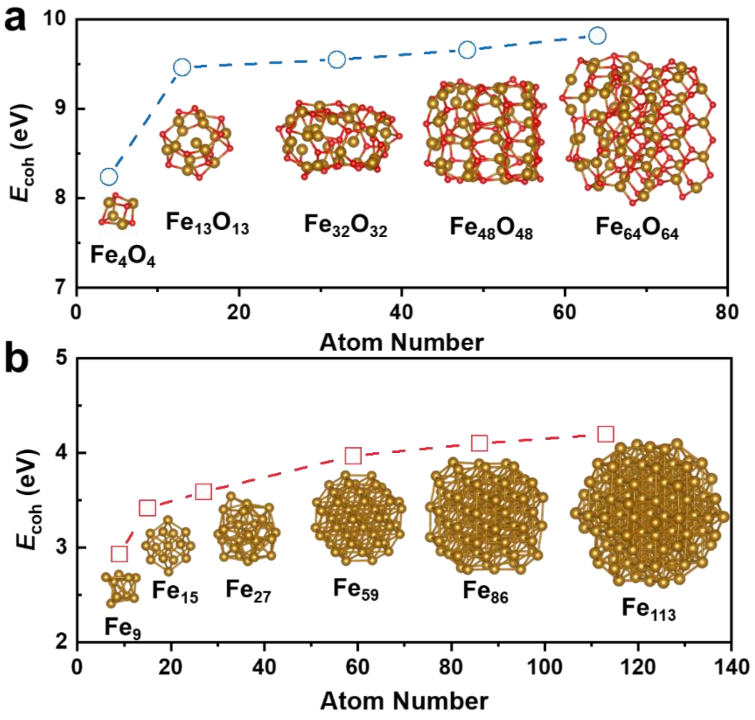

Figure R5. The cohesive energy of model molecules with different atomic numbers. (a) Fe_nO_n models, (b) Fe_n models.

Regarding the critical 7 nm value for the decomposition of FeO_x particles, we recognize that other factors such as heating temperature, atmosphere, and local environments play significant roles. For example:

i. Heating Temperature: Higher temperatures can accelerate the decomposition of FeO_x particles by providing the necessary thermal energy to overcome kinetic barriers. However, excessively high temperatures might lead to sintering, which could adversely affect the dispersion of single Fe sites.

ii. Atmosphere: The presence of reducing or oxidizing gases can significantly influence the conversion process. For instance, a reducing atmosphere (e.g., H_2) can facilitate the reduction of FeO_x to metallic Fe, while an oxidizing atmosphere (e.g., O_2) might stabilize the oxide form, hindering the formation of single Fe sites. Our recent work reported the conversion of high-density Fe-N_x sites at as low as 360 °C in a hydrogen pyrolysis atmosphere (**Figure R6**) [*Angew. Chem. Int. Ed. 2024, e202404766*].

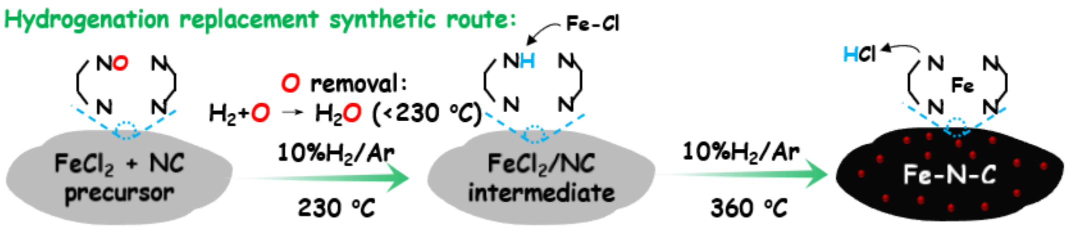

Figure R6. The schematic diagram of the low-temperature hydrogen replacement synthetic route [*Angew. Chem. Int. Ed. 2024, e202404766*].

iii. Local Environments: The local chemical environment, including the presence of support material and any potential doping elements, can affect the stability and distribution of single Fe sites. The interaction between Fe atoms and the support matrix can dictate the final configuration and activity of the catalytic sites.

We have conducted comprehensive computational studies to investigate these factors in detail. The results from these simulations have been integrated into our revised manuscript to provide a more robust understanding of the active site formation process (Supplementary Figs. 36-38).

Thank you once again for your valuable feedback. We believe that addressing these theoretical aspects will significantly enhance the scientific rigor and impact of our work.

(2) Frédéric Jaouen et al reported there are two types of FeN_4 sites in Fe/N/C catalysts denoted as S1 and S2 sites (Nature Catalysis volume 4, pages 10–19 ,2021). The authors may further elucidate how the experimental conditions affect the formation of these two active sites during the FeO_x conversion to single metal sites.

Response 2:

Thanks for the value comment. According to Frédéric Jaouen *et al.*, who identified the active sites of Fe-N-C catalysts by Mössbauer spectroscopy and XAS as shown in **Figure R7** [*Nat. Mater., 2021, 4, 10-19*], they think that there are mainly two types of sites in the Fe-N-C material: the highly active but lowly stable Site S1 (D1) and the lowly active but highly stable Site S2 (D2). From *in-situ* ^{57}Fe Mössbauer spectroscopy in inert gas coupled to calculations of the Mössbauer signature of FeN_x moieties in different electronic states, they identify D1 to be a high-spin $Fe^{III}N_4C_{12}$ moiety with axial oxygen ligand (doublet with quadrupole splitting (QS) values of 0.9-1.2 mms^{-1}) and D2 a low- or intermediate-spin $Fe^{II}N_4C_{10}$ moiety (QS=1.8-2.8 mms^{-1}), both having a similar isomer shift.

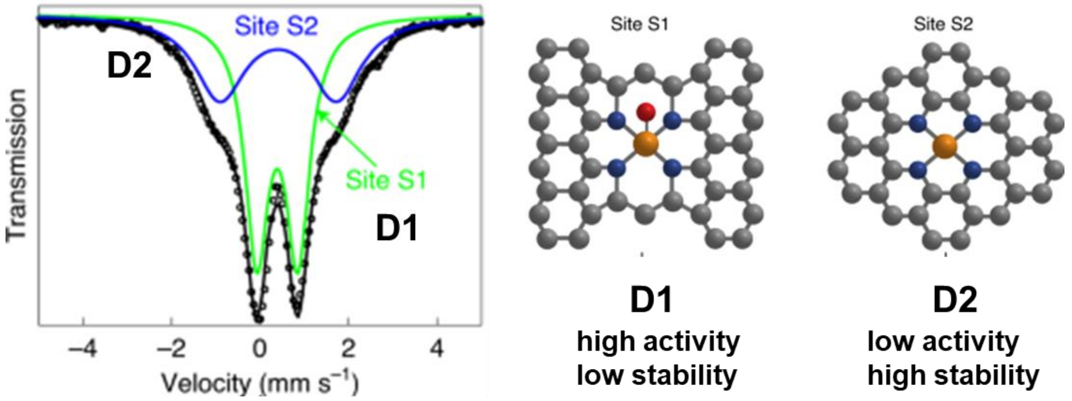

Figure R7. Site S1 (D1) and Site S2 (D2) identified by Jaouen *et al.*

Upon careful examination of the configurations of the two sites, it becomes evident that the S1 site features a FeN_4C_{12} configuration, coordinated by four pyrrolic nitrogen and containing a pair of carbon defects. In contrast, the S2 site is characterized by a FeN_4C_{10} configuration, coordinated by four pyridinic nitrogen and embedded within the complete graphene structure. Therefore, in the conversion process of FeO_x to FeN_x, the carbon thermal reaction would naturally consume part of the carbon and produce a large number of carbon defects, which we confirmed by Raman spectroscopy (**Figure R8**), so the conversion process tends to preferentially form a highly active S1 site. To compensate for the carbon atoms consumed during the carbon thermal reaction in the conversion process, a chemical vapor deposition (CVD) strategy can be employed to supplement additional carbon atoms. For instance, Wu *et al.* [*Nat. Energy 2022*, *7*, *652-663*] and Xing *et al.* [*Nat. Commun. 2024*, *15*, *4219*] used ZIF-8 and cyanamide as supplementary carbon sources, respectively, and further deposited them onto Fe-N-C catalysts to facilitate the conversion of S1 sites to S2 sites, particularly those on the outer surface. Their experimental results confirm the effectiveness of the CVD strategy, demonstrating that Fe-N-C-CVD catalysts can operate stably for over 200 hours at 0.67 V (**Figure R9**). Hence, supplementing Fe-N-C with additional carbon sources is essential for promoting the conversion of S1 sites to S2 sites on the outer surface.

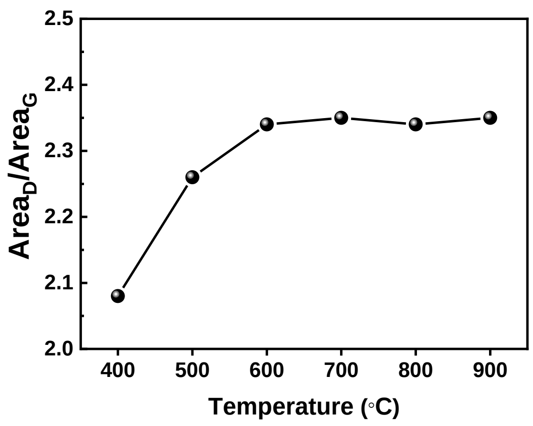

Figure R8. $Area_D/Area_G$ of 0.015Fe-NC-*T* samples obtained after thermal activation at different temperatures.

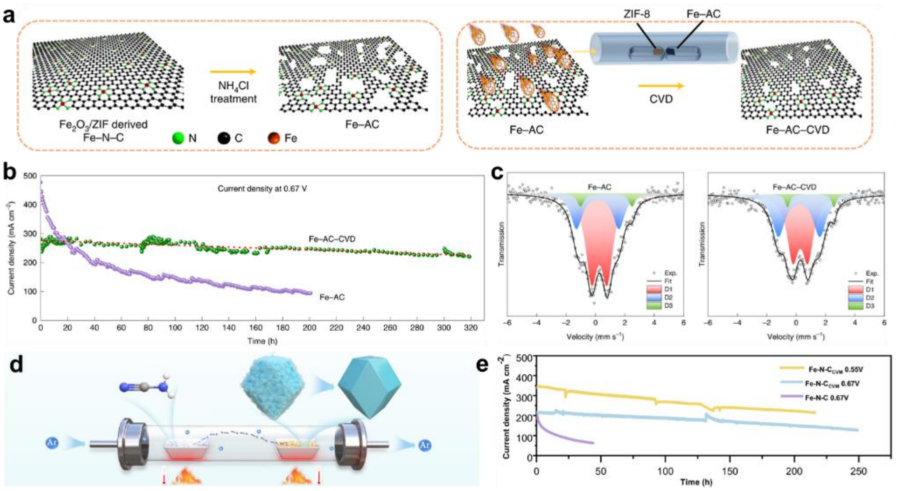

Figure R9. (a) Schematic diagram of the synthesis, (b) long-term fuel cell life tests under H_2-air conditions at a constant potential of 0.67 V and (c) the ^{57}Fe Mössbauer spectra of Fe-AC and Fe-AC-CVD catalysts [*Nat. Energy 2022, 7, 652-663*]. (d, e) Schematic depiction of the synthesis and long-term fuel cell tests for Fe-N-C_{CVM} and Fe-N-C electrocatalysts under H_2-Air conditions at a constant potential of 0.67 V [*Nat. Commun. 2024, 15, 4219*].

(3) Currently, Fe/N/C catalysts face grand stability challenges. The authors may explain the possible degradation mechanisms of the FeO_x-derived catalysts and the possible strategies to enhance catalytic stability under ORR conditions.

Response 3:

Thanks for the value comment. The catalytic stability of the FeO_x-derived catalysts has always been a concern, we have the following discussion:

1) The negative relationship between activity and durability of Fe-N-C electrocatalysts

While significant progress has been made in the past decade in terms of the initial activity and power performance of Fe-N-C catalysts, there has been limited advancement in their stability and durability. Additionally, a negative relationship has been observed between the initial ORR activity of Fe-N-C catalysts and their durability, especially in the fuel cell application [*Chem. Rev. 2016, 116, 6, 3594–3657*; *ACS Catal. 2021, 11, 2, 484–494*]. As shown in **Table R**1, we have studied the stability of highly active Fe-N-C catalysts that have been published in recent years. Fe-N-C catalysts with a high microporous area and high single-atom loading have shown accelerated performance degradation over time, which is particularly prominent for those that were initially more active. Only a few Fe-N-C materials with low initial activity have demonstrated promising durability in PEMFCs, although their performance still falls significantly below that of ultralow Pt cathodes (blue dotted frame). While some Fe-N-C cathodes with favorable ORR mass activity have shown limited performance degradation during operation in the PEMFCs for several hundred hours, durability tests

were typically conducted under low-potentiostatic conditions (orange dotted frame), indicating a mixed control by ORR kinetics and cathode mass transport.

Table R1. The recently reported highly active M-N-C catalyst and its stability.

Catalysts	P_{max} in air ($W\ cm^{-2}$)	*J*@0.8V in air ($mA\ cm^{-2}$)	Stability in PEMFCs	Ref
Fe_g-NC/Phen	0.71	120.8	> 80% loss after 25 h at 0.6 V (H_2-air)	*Energy Environ. Sci., 2022, 15, 3033-3040*
Fe-AC	0.60	151	> 80% loss after 200 h at 0.67 V (H_2-air)	*Nat. Energy, 2022, 7, 652-663*
Fe-MOF catalyst	0.61	50	37% loss after 40 h at 0.7 V (H_2-air)	*ACS Appl. Mater. Interfaces. 2020, 12, 2216*
SA-FeN_x-ZIF8-PCM	0.60	100	34% loss after 50 h at 0.4 V (H_2-air)	*Nano Energy 2021, 83, 105734*
Fe/N/C(4mIm)-OAc	0.47	40	> 80% loss after 35 h at 0.5 V (H_2-O_2)	*Adv. Funct. Mater. 2021, 31, 2009645*
TPI@Z8(SiO_2)-650-C	0.42	105	> 80% loss after 50 h at 0.5 V (H_2-O_2)	*Nat. Catal. 2019, 2, 259-268*
Fe–$NC^{\Delta\text{-}DCDA}$	~0.45	~90	60% loss after 10 h at 0.7 V (H_2-air)	*Nat. Catal. 2022, 5, 311-323*
PANI-FeCo-C	< 0.3	340@0.4V	No loss after 700 h at 0.4 V (H_2-air)	*Science 2011, 332, 443-447*
Fe/N/CF	< 0.3	340@0.5V	~30% after 100 h at 0.5 V (H_2-air)	*PNAS 2015, 112, 10629-10634*
SA-FeN_x-ZIF8-PCM	0.60	100	34% loss after 50 h at 0.4 V (H_2-air)	*Nano Energy 2021, 83, 105734*
Fe-AC-CVD	0.52	85	13% loss after 319 h at 0.67 V (H_2-air)	*Nat. Energy, 2022, 7, 652-663*
Fe-N-C-FG	0.72	191	50% loss after 200 h at 0.67 V (H_2-air)	*Nat. Catal., 2023, 6, 1215-1227*

2) The possible degradation mechanisms of Fe-N-C electrocatalysts

According to the review of Jaouen [*Chemical Reviews, 2023, 123, 9265-9326*], the four degradation mechanisms of Fe-N-C oxygen reduction electrocatalysts in acid medium.

i) De-metallization of the Fe-N-C Catalyst

In RDE conditions, Fe site dissolution mainly reduces the intrinsic activity of Fe-N-C catalysts. However, in fuel cell operation, dissolved Fe ions not only can weaken the ORR but also affect the catalyst layer's conductivity by exchanging with H^+ ions in the Nafion electrolyte. This can dramatically deteriorate the current-voltage output in the PEMFCs when the proton diffusion cannot be fast enough. By contrast, the abundance of aqueous supporting electrolyte (H_2SO_4) during RDE testing minimizes the impact of Fe ions on proton transport and catalyst layer's conductivity.

ii) Water flooding in the micropores

In the RDE test, O_2 is present in a dissolved state, and O_2 saturated water environment equilibrates in both micro- and mesopores, allowing constant diffusion rates of O_2 and proton. The mass diffusion of O_2 and proton across the solid-liquid interface can be maintained well throughout electrochemical cell by rotating electrode. Therefore, the transport of O_2 and proton from bulk solution to the active sites at the exterior surface and pores of the catalyst cannot be an issue in the RDE test. In the PEMFCs operation, O_2 is introduced in a gaseous state. At the beginning, the active sites in the micropores can be completely available to O_2 and solvated proton and an appreciable ORR rate can be obtained. However, the micropores have to come into a new equilibrium on the reactant distributions and solvation environment as the fuel cell runs. When a large amount of water produced from the ORR cannot be discharged from the catalyst in time, gaseous O_2 needs to dissolve and migrate to the active sites. That water flooding can severely hinder O_2 gas transport to the active site, leading to a substantial decrease in current density. Besides, the high catalyst loading is required in the PEMFCs test, so that a thick catalyst layer is compressed to prepare the MEA, probably causing a high resistance to the water discharge in the MEA. Moreover, when the pore networks are not homogeneously distributed in the MEA, the flooding can become evolve very severely as the fuel cell runs. We have performed the MEA preparation with a conventional method and in a commercial standard fuel cell setup, which is similar to the relevant research from other groups. However, a special design of hydrophobic property in the MEA preparation can significantly alleviate the flooding in the micropore and relatively improve durability performance in the fuel cell test, which needs to be focused on in the fuel cell research.

iii) Oxidation of the carbon matrix

Carbon is the major element in M-N-C catalysts, representing typically 90 at% of the material's composition. The carbon matrix covalently hosts the M-N_x sites, serving as the long-range electronic conductor for both types of active sites [Adv. Mater. 2019, 31, e1805126]. Hence, surface or bulk carbon oxidation reaction (COR) during operation of M-N-C catalysts can affect the TOF, selectivity, stability/durability of active sites, and also the electronic conductivity of the entire active layer as well as its mechanical integrity in the case of extended bulk COR. A clear distinction between surface-restricted COR (eq 1, eq 2, and eq 3 restricted to a monolayer, with oxygen functional groups formed on the top surface) and bulk COR leads to the irreversible formation of gaseous CO2 and CO (eq 4 and eq 5).

$$\mathrm{C\ (s) + H_2O\ (l) \rightarrow C\text{-}OH\ (s) + H^+ + e^-} \qquad \text{eq (1)}$$

$$\mathrm{C\text{-}OH\ (s) \rightarrow C{=}O\ (s) + H^+ + e^-} \qquad \text{eq (2)}$$

$$\mathrm{C{=}O\ (s) \rightarrow C\text{-}OOH\ (s) + H^+ + e^-} \qquad \text{eq (3)}$$

$$\mathrm{C\ (s) + 2H_2O\ (l) \rightarrow CO_2\ (g) + 4H^+ + 4e^-} \qquad E^o = 0.207\ \mathrm{V}\ vs.\ \mathrm{SHE} \qquad \text{eq (4)}$$

$$\mathrm{C\ (s) + H_2O\ (l) \rightarrow CO\ (g) + 2H^+ + 2e^-} \qquad E^o = 0.512\ \mathrm{V}\ vs.\ \mathrm{SHE} \qquad \text{eq (5)}$$

Mild corrosion of the carbon matrix yields formation of oxygen-containing surface groups, depletion of the delocalized π electrons, and the drop of the TOF of the M-N_x moieties [*Energy Environ. Sci. 2018, 11, 3176-3182*]. More severe carbon corrosion leads to a decrease of the pore volume and the pore diameters, increasing the hydrophilic character and the destruction of the M-

N_xC_y active sites. The COR is thermodynamically favorable from 0.2 V *vs.* SHE but kinetically slow [*ACS Catal. 2014, 4, 2258-2267*], hence the COR is a severe problem during start/stop periods, in which the local cathode potential can reach as high as 1.5 V *vs.* RHE. Previous studies on Pt/C catalysts have shown that graphitic carbon supports are the most robust toward carbon corrosion due to lower content of structural defects, at which corrosion initiates [*ACS Catal. 2015, 5, 2184-2194*].

iv) H_2O_2 and oxygen radical species concentration

In addition, we assume that the kinetic aspect of the ORR at the catalyst can in principle be equal work in the MEA as in the RED measurement. However, the slow transport of proton (or locally very low proton concentration) in fuel cell can lead to a relatively long lifetime of the ORR intermediates, such as oxygen radical species and H_2O_2, which can substantially accumulate locally around the triple-phase boundary (TPB). That can result in the side reaction with the carbon skeleton of the catalyst and then the dissolution of active site, which is detrimental to the catalyst stability and contributes to the overall performance decaying in the fuel cell test. That degradation tendency can be magnified in the MEA when a relatively high catalyst loading is applied, as the aforementioned high resistance to the mass transport in the compressed MEA. It should be noted that the transport of proton and O_2 is in a perfect condition in the RDE test and the catalyst loading is much lower than in the fuel cell test. Therefore, the oxygen radical species and H_2O_2 that are once generated can be largely transferred to the active site for a further ORR reaction to H_2O in the RED test. Correspondingly, the carbon skeleton decomposition and the active site dissolution can be alleviated to some extent. Nonetheless, it is inevitable that the oxygen radical species and H_2O_2 have retention time to cause the decomposition of carbon skeleton and the dissolution of active site during the ORR in the long term. A good mass transport condition can significantly alleviate those issues as discovered in the RDE test.

3) The possible strategies to enhance catalytic stability

i) Mitigation of surface and bulk carbon corrosion

Increasing the degree of graphitization of M-N-C materials is therefore a promising solution to improve their durability [*Adv. Energy Mater. 2018, 8, 1801226*]. As more graphitic materials are also more hydrophobic, this approach also reduces the probability of flooding the cathode active layer. Nevertheless, increased degree of graphitization requires temperatures between 1700 and 3000 °C, which is detrimental to the surface concentration of pyridinic- and pyrrolic-N (eventually leading to a drop of the TOF), and to the atomic dispersion (aggregation of the transition metal atoms leading to a drop in the SD value). The right trade-off between durability (more graphitic structure, i.e., low specific surface area) and mass activity for the ORR (high SD + high TOF) must therefore be found. Wu et al. prepared different PANI-based Fe-N-C catalysts, with different carbon supports, such as Vulcan XC-72, BP-2000, KJ-300J, and multiwall CNTs, and reported that the CNTs-based MEAs were the most stable after 500 h at 0.4 V under O_2 environment (80 °C) (**Figure R10**) [*ECS Trans. 2009, 25, 1299-1311*].

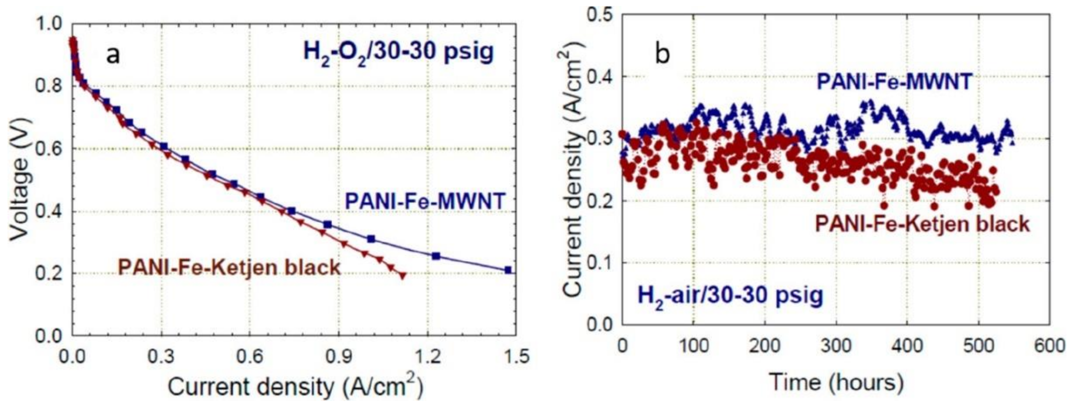

Figure R10. Fuel cell performance of PANI-Fe catalyst obtained using MWNTs and Ketjenblack as supports: (a) initial polarization plots, (b) life tests [*ECS Trans. 2009, 25, 1299-1311*].

ii) Selective formation of durable Fe-N$_4$ sites

After Jaouen identified the more durable Site 2 and the less stable Site 1 in a PEMFCs using ^{57}Fe Mössbauer spectroscopy [*Nat. Mater., 2021, 4, 10-19*], the subsequent strategy focused on tailoring the synthesis of Fe-N-C materials to preferentially generate the robust Site 2, which offers a graphene-like extended environment for Fe-N$_4$ in contrast to the porphyrin-like Site 1. This appealing concept appears to have been realized in Wu *et al.*'s recent work [*Nat. Energy 2022, 7, 652-663*], which documents a significant advancement in Fe-N-C durability. Their work independently corroborated the stabilization effect of an N-C overlayer, although it highlighted a trade-off between ORR performance and stability for this method. The Fe-N-C was synthesized from a Fe_2O_3@ZIF-8 catalyst precursor, undergoing an initial pyrolysis in argon followed by a secondary pyrolysis with ammonium chloride salt. Subsequently, the catalyst (Fe-AC) was further modified by positioning nanometric ZIF-8 at upstream of the Fe-AC, under inert gas flow at 1100 °C. ZIF-8 decomposition at elevated temperatures generated volatile compounds that partially redeposited on the Fe-AC surface, resembling a chemical vapor deposition process (Fe-AC-CVD) (**Figure R11a**). PEMFCs tests confirmed the enhanced durability during a potential hold at 0.67 V (**Figure R11b**). Mössbauer spectral analysis with three spectral components indicated an increased proportion of D2 (site S2) in Fe-AC-CVD (**Figure R11c**), implying that the ZIF-8 CVD altered the coordination environment of the surface Fe-N$_4$ sites in the Fe-AC. Despite the changes in relative fractions of D2 against D1 do not appear as pronounced as one might anticipate, given the notable improvement in material durability. It is important to remember that Mössbauer spectroscopy is a bulk probing technique encompassing all Fe atoms in the catalysts. Therefore, the shifts in D1 and D2 quadrupole doublets on the surface of Fe-AC and Fe-AC-CVD might be more substantial than what are experimentally detected. Further advanced characterizations revealed that Fe-AC-CVD possesses a higher proportion of sp^2 carbon, with additional thin graphitic layers detected on its surface. Wu *et al.* inferred that the CVD process transformed some S1 sites into the more resilient S2 sites and also produced a carbon surface that is better ordered and more resistant to electrochemical oxidation. Disentangling these two impacts is challenging since the CVD process simultaneously modifies the coordination of surface Fe-N$_4$ sites and the carbon surface's morphology. The observed high durability could stem from the combined influence of these factors,

or it is possible that one effect predominates, significantly enhancing the durability. Recently, Xing *et al.* employ the chemical vapor modification method to alter the configuration of active sites, along with enhancing the degree of graphitization in the carbon substrate. When subjected to operational conditions representative of fuel cell systems, the electrocatalyst displayed remarkable durability, sustaining stable performance for a duration exceeding 248 h (**Figure R11d**, **e**).

Recently, Wu *et al.* have refined the synthetic pathway by incorporating a measured quantity of hydrogen into the conventional inert atmosphere, resulting in the creation of an Fe-N-C catalyst (denoted as Fe-N-C-FG) [*Nat. Catal. 2023, 6, 1215-1227*]. The study reveals that hydrogen not only markedly elevates the overall density of Fe-N_4 sites but also suppresses the formation of the less stable pyrrole nitrogen coordination at Site 1, favoring the more stable pyridinic nitrogen coordination at Site 2, which is characterized by a reduced Fe-N bond length (as depicted in **Figure R12a**). Consequently, the Fe-N-C-FG catalyst exhibits not just a notable enhancement in activity but also improved stability, as illustrated in **Figure R12b**.

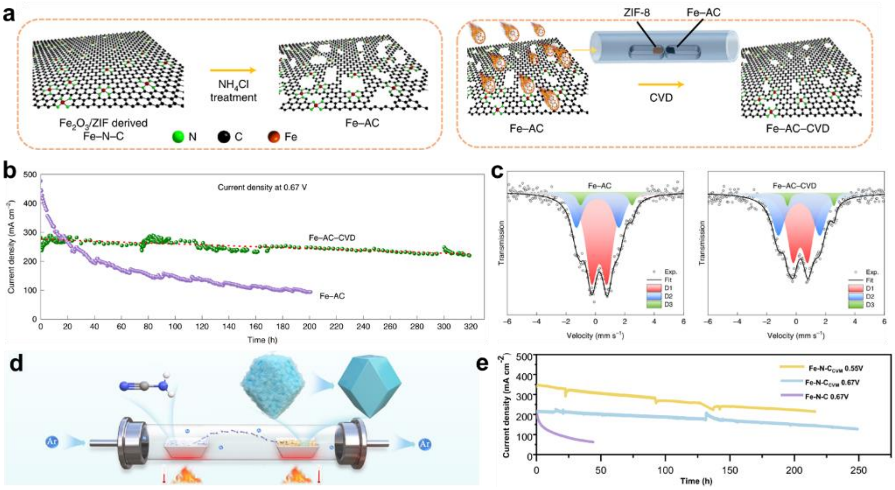

Figure R11. (a) Schematic diagram of the synthesis, (b) long-term fuel cell life tests under H_2-air conditions at a constant potential of 0.67 V and (c) the ^{57}Fe Mössbauer spectra of Fe-AC and Fe-AC-CVD catalysts [*Nat. Energy 2022, 7, 652-663*]. (d, e) Schematic depiction of the synthesis and long-term fuel cell tests for Fe-N-C_{CVM} and Fe-N-C electrocatalysts under H_2-Air conditions at a constant potential of 0.67 V [*Nat. Commun. 2024, 15, 4219*].

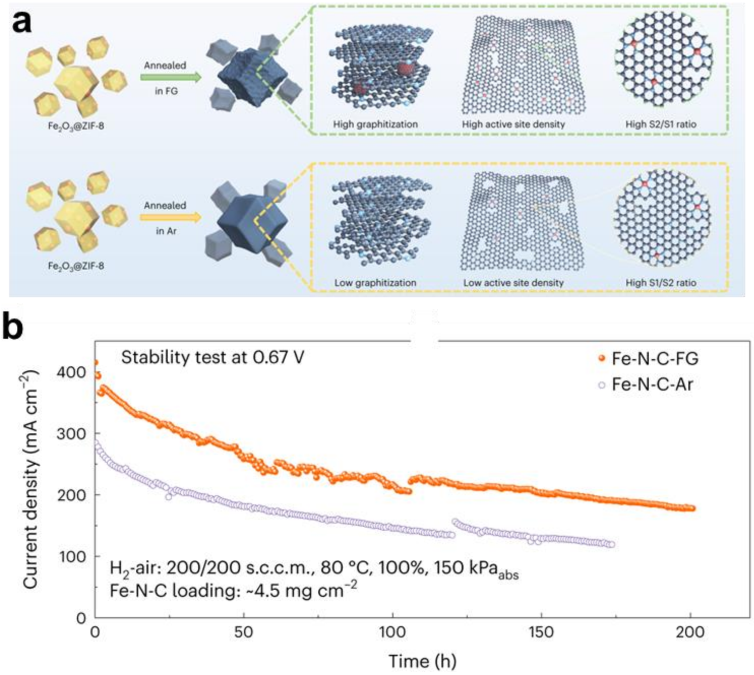

Figure R12. (a) Schematic of the construction of S1 and S2 sites via control of the pyrolysis atmosphere; (b) Long-term fuel cell life tests under H_2-air conditions at a constant potential of 0.67 V [*Nat. Catal. 2023, 6, 1215-1227*].

Reviewer #2 (Remarks to the Author):

The authors have addressed this reviewer's concern. The paper can be accepted for publication.

Response:

We sincerely thank you for the positive feedback and support the publication of our work.

Reviewer #3 (Remarks to the Author):

All concerns have been addressed in their communication with the reviewers and reflected in the revised manuscript.

Response:

We sincerely thank you for the positive feedback and encouraging of publication of our study.

REVIEWERS' COMMENTS

Reviewer #1 (Remarks to the Author):

Thank the authors for the great effort and time in replying to all previous comments. The revised manuscript is now suitable for publication in the journal.
